# Supplementary material for: Female cyclists perceived effects and experiences of the menstrual cycle on training and performance
Source: PLoS One. 2026 Jun 2;21(6):e0343892. doi: 10.1371/journal.pone.0343892 (PMC13229329; doi:10.1371/journal.pone.0343892)
Supplement: S1 Text — S2 File. Interview guide. S3 File. Interview transcripts. (ZIP) [file pone.0343892.s001.zip › Supporting information/S1 File - pre-interview questionnaire.docx]

# General

1. What is your date of birth?
2. How many years have you been cycling?
3. What events do you compete in?
4. Approximately how many times a year do you race?
5. How many training sessions do you complete in a typical week? What length are these sessions?
6. What is your average total training time per week (hrs)?
7. Do you have a male or female coach, and how long have they coached you?
8. What bicycles (make and model) do you ride (for training, racing and different events)? Are they women’s specific?
9. What saddles (make and model) do you use? Are they women’s specific? Do you use different saddles on different bikes, if so, specify which saddles you use on which bikes?

# Menstrual cycle

1. How old were you when you had your first period?
2. Do you currently use any hormonal contraceptives (e.g., oral contraceptive (pill), contraceptive injection, contraceptive patch, vaginal ring, contraceptive implant, or intrauterine system (IUS))?
3. If you answered **NO** to question 2, have you previously used any hormonal contraceptives?

If yes, what type of hormonal contraceptive and reason you did not continue using it?

**If YES to question 2 please go to question 10:**

1. How often do you get your period (i.e., days from first day of period to the first day of the next)?
2. How long do your periods (bleeding) last (e.g., number of days)?
3. Are your periods regular (i.e., more than 9 a year / period every 21-35 days)?

If your periods are irregular, typically how many do you have a year?

Have you missed a period consistently for the last 3 months?

1. Do you track your menstrual cycle (i.e., counting the days between your bleeds), and if yes for how long and using what method?
2. What date did you start your last period?
3. Do you typically suffer with any of the following symptoms related to your menstrual cycle? (Tick, highlight or circle relevant symptoms)

Changes to /difficulties breathing

Nausea, sickness, and vomiting

Constipation

Dizziness/ light headiness/ reduced co-ordination

Poor concentration/ memory

Joint pain/ muscle aches and cramps

Temperature fluctuations

Disturbed sleep

Diarrhoea

Headaches/ migraines

Lower back pain

Water retention

Bloating/ increased gas

Period cramps / pain and pelvic / uterine / ovarian pain

Tiredness/ fatigue

Breast pain/ tenderness

Cravings/ changes in appetite

Mood changes/ irritability/ anxiety

**For hormonal contraceptive users only**

1. What hormonal contraceptive do you use?
2. When did you start using hormonal contraceptives?
3. Do you experience withdraw bleeds (light blood flow longer than a day which requires the use of menstrual products)?
4. What is the reason for using this contraceptive, e.g., as a contraceptive, to manipulate bleeds around training, reduce period related symptoms?
5. Have you had any associated perceived side-effects to taking this method of hormonal contraception on your general health and on your cycling performance?
6. Have you used any other contraceptives and when/ why did you change, did you discuss this with a medical professional?
7. Are you aware of different contraceptive options that are available to use?
