# Supplementary material for: Female cyclists perceived effects and experiences of the menstrual cycle on training and performance
Source: PLoS One. 2026 Jun 2;21(6):e0343892. doi: 10.1371/journal.pone.0343892 (PMC13229329; doi:10.1371/journal.pone.0343892)
Supplement: S1 Text — S2 File. Interview guide. S3 File. Interview transcripts. (ZIP) [file pone.0343892.s001.zip › Supporting information/S3 File - Interview transcripts.docx]

**P1 – interview transcript**

0:0:0.0 --> 0:0:15.150
*Interviewer*
So just a bit of a background about cycling. So, what events do you currently competing? I know you did the bit of the pre interview questionnaire, and have you always been a cyclist, or did you do other sports before you started cycling?

0:0:15.920 --> 0:2:24.550
*P1*
No, not always been a cyclist, so I used to run quite a competitive level, so I was with a Harriers for a few years running with them and I ended up with a sort of severe ankle injury from running, so came down very badly on my ankle and had chronic nerve damage and kind of used cycling as a bit of active recovery actually to get back into things like aerobic wise without any sort of real, sort of pressure, injury or anything like that.

I just got into it as a complete novice and signed up with a club and just started doing rides from scratch and building it up, just literally pulled training plans off with British Cycling's website and stuff, like 8 week training plans and that and then just started doing local time trials, local hill climbs and sort of found road races to be where I was happiest. So, crits, road races and stuff riding in a bunch, tactics. You know a lot more involved than just sheer numbers, and its constant pace, like in time trials and stuff that hill climbs.

So, I've only really been riding competitively for about five years now, so not too long in the grand scheme of things, pretty fresh still. And I mean I'm 33 now, so you sometimes find with the age thing it's difficult to get into certain teams or you know the kind of the structure that teams build their focus around with young riders and junior riders and bring them through the rankings and stuff. But at the I currently say I am pretty experienced, but still inexperienced in hindsight. And so yeah, I've only been at, say, competitively racing for about five years now. Training wise, I'm probably near enough full time with that. I do six days a week in terms of riding on the roads and then S&C work gets dragged into that as well, two to three times a week with that on top of it. So, yeah, relatively new, but still got a fair bit of experience in the bag, you know…..

0:5:14.220 --> 0:5:29.510

*Interviewer*
Ok, I'll start, I'll move on to some of the questions around the menstrual cycle. So, when you first got your period, how old were you? Were you playing sport then, did it have any effect?

0:5:30.540 --> 0:6:14.10
*P1*
I was 12, so I think that's probably around average time really at that time 12/13. I've always been pretty sporty. I wouldn't necessarily know if I was playing sport as such, but you know sports day and athletics and everything was always something that I did from a young age and I've always been like quite naturally gifted with sports, like a bit of an all-rounder. I couldn't say if I remembered it affecting anything at the time, it probably was an inconvenience, I would say, but I wouldn't be to tell whether affected performance or anything like that at such a young age. You know, I wasn't really that committed to sports then to really care that much about it, I don't think.

0:6:14.920 --> 0:6:22.270
*Interviewer*
Did you get any advice at that time around periods/menstrual cycle? Was it from school or elsewhere?

0:6:23.600 --> 0:6:46.940
*P1*
Yeah not, I wouldn't say it was really talked about that much, like even with me mam and stuff. I don't even think it was a subject that was actually sat down and spoken, like I was aware of it and stuff, but I'd say probably school and that not even friends, though I don't even think it was spoken about then, I think it's more when you have like you know, your sex education classes and stuff at school which will always a right hoot. You know, there were. You were there for a laugh. Really, weren't you? But yeah, just stuff, you know, even like, still learning things, up until you know mid-20s, late 20s, like the actual ovulation cycle and stuff like, it's happened. I'm aware it happens, but I've never really looked into the phases and what's exactly happening and what goes on with the body like it's, you know, it's a very unspoken about subject and the effects it has. And I'd say more recently than ever, I've learned more about it than just sort of it happening and knowing it happens and then you just cracking on with things you know?

0:7:23.670 --> 0:7:31.960
*Interviewer*
Yes. As you've got older, has your menstrual cycle stayed relatively similar or has it or have you experienced any changes?

0:7:33.670 --> 0:8:23.170
*P1*
So for the past few years, it's been pretty consistent. I mean, I was on the depo for a long, long time, so I didn't actually have periods for ages. You know, it was, it was actually very convenient. I was fine, you know? But then don't really. Kind of get to a part with point where you think like it makes you feel a little bit more normal being a bit more in sync actually having the cycles and stuff. So, I'd say for the past, about the past. When was the pandemic and stuff 2021/2020, so since then? So yeah, four years or so, I'd say it's been pretty consistent. Regular, I don't think there's been anything that's really alarmed us, in it being anything out of the normal, you know. So yeah, pretty, pretty normal. I would say as far it, whatever normal is.

0:8:23.240 --> 0:8:36.620
*Interviewer*
Yeah, yeah, obviously coming off the depo has that had having a more regular menstrual cycle, normal mental cycle, has that had any effect on you as an athlete or have you had to make any changes around that?

0:8:37.900 --> 0:10:10.40
*P1*
I wouldn't say changes as such. Obviously, it does affect performance in a way in which obviously symptoms that come with it and stuff, I mean there was I was speaking to someone who was doing a master's degree in a similar type of thing, and that's what made me more aware of it. Like I started tracking it and started seeing if the I started looking into it more, so there's obviously phases in which if you do more strength work it can benefit you, the type, the amount of estrogen and your system and stuff like that. So, I started looking into a bit more and I think there's times where you do training, and you think I'm absolutely flying, and you just think it's I mean, there's a lot of control factors in the depend on hydration, how much you're sleeping, your fuelling, calorie deficit and stuff, but there's times where you're training and you're feeling really, really good and you think nothing more of it, then there's times when you feel rubbish and you just you know it's, it happens to everyone. You know, you're not always gonna feel great, but there is that link with it that I don't think you put two and two together. You just you feel that way because you do that day for whatever reason. But I'd say that is probably the more you track it, the more obvious it becomes at a certain part of your phases where you actually feel like you're flying and you’re going really well or times where you feeling quite sluggish and stuff and you're looking for answers elsewhere where really I think it's, you know, it’s part of your cycle where you're feeling drained for a reason, you know, you know, you're not everything's in it.

0:10:11.170 --> 0:10:16.610
*Interviewer*
And based on the tracking and the different symptoms, have you modified your training to accommodate those?

0:10:17.260 --> 0:11:18.910
*P1*
I haven't. I've always wanted to, so I had a coach a few years ago and he was a male coach and I remember speaking to him about that then. I wanted to introduce it like I as a bit of a trial run and he I was his only female athlete, and I found a lot of the training and was very, it wasn't tailored. It was very generic, the training, so I think, I don't feel like coaches really take it into context like and use it to benefit the athlete because and I know they definitely, it definitely can come into that. And I did want to do it with him, and it would just never really. It never really took off, and then I've had female coaches since then two, and it's just never been brought up. It's never been thought about using it, so I don't know if they know much about the research into it or whether they think it's really that beneficial or whether they just stick to training towards your target and go with the aerobic system and fatigue levels. And you know, just stick to that side of it.

0:11:20.80 --> 0:11:30.410
*Interviewer*
Yeah. In terms of these, you mentioned some symptoms. So, what kind of symptoms do you get? Are they in different phases or they all around kind of menstruation, premenstrual?

0:11:31.800 --> 0:12:35.380
*P1*
Yeah. So, I get quite bad cramps. So that's like sometimes it's intermittent - like some cycles it can be like absolutely crippling and other ones not so bad, like I can't get on with things like couple of painkillers, and were sometimes literally just need like half an hour for a painkiller kick in and I'm just curled up, you know, I just, literally walking around just too sore, like, I couldn't even imagine riding around on a bike and stuff, so that would be the only time in which it would really get in the way. Sometimes ovulation, that's that phase can cramp a little bit with that and it's quite obvious when it's around ovulation date type of thing. But I wouldn't say it really affects anything. I'd say more the, day one would probably be the most symptomatic and probably the most restrictive in terms of just pulling myself together a bit and getting out and about, you know, because it's you just wanna lie, lie down, don't you? And curl up in a ball really. Sometimes when they're that bad, you don't make it feel sick. Somethings, doesn't it, the pain.

0:12:36.490 --> 0:12:42.70
*Interviewer*
So obviously, you've mentioned you take painkillers, so would it be, would it take them for a few days typically or?

0:12:43.0 --> 0:13:3.110
*P1*
Yeah, I'd say probably nearly every four hours on the day one. And then the second day probably be a couple of times as the day goes on, it would start to then ease, normally day three and four I don't really have any cramp and stuff. It's the first day and half maybes and so yeah, just paracetamol every four hours with that.

0:13:4.320 --> 0:13:10.630
*Interviewer*
If that happens on a race day and how do you get round that? Or do you just take as many paracetamol, as you can and get on with it?

0:13:10.530 --> 0:13:39.940
*P1*
Haven't actually had it happen yet, which seems absolutely bizarre. I don't know how it's always missed the first day. There's been times where I've thought that was close, like if I finished a race and stuff and I've started spotting on a little bit, I think like that could have been close that. But I think it would just, yeah, genuinely be painkillers and just have to get on with it. I guess it does start with the kick in within half an hour and ease a little bit, but I can just imagine to be unpleasant like a training ride would be.

0:13:40.850 --> 0:13:57.790
*Interviewer*

In terms of that as well, if you're on your period, how do you manage that with long rides? Do you do any particularly long rides? Do you have to kind of t plan that around toilet stops or whatever if you're on your period or does are not really isn't all factor, or you're not that heavy bleeding.

0:13:58.740 --> 0:14:32.350
*P1*
Not that heavy, but it does definitely become like a bit of an itinerary for the day. You know, I've got to think about, like, leaving it as late as possible and before going out and then just timing it, so that I've got like maybes. So, my long rides aren't really more than 4.5hours, so that tends to if I literally before I go out and then and get back within the 4.5hours tends to be OK. I haven't had any sort of mishaps with that. So, you I do have to change timings around. Yeah, just to fit in with that, yeah.

0:14:33.300 --> 0:14:45.900
*Interviewer*
OK. Umm do you get any? Obviously, we've talked about some of the physical symptoms, but does it have any effect on your mood or emotionally and do you kind of change in various points in the cycle or is that not a symptom for you?

0:14:46.850 --> 0:15:42.490
*P1*
Yeah, I'd say there's a symptom. You are probably better off asking me boyfriend with that. He well knows. Uh, yeah, very, very irritability. Straight away, like everything, just annoys us. Let's yeah. You it's just sort of. Yeah. My mean mood changes. I think you tend to feel. Yeah, just agitated, irritable, and I've also find, like, very, I don't know if this is even a symptom, but I find sort of premenstrual going. just before ovulation stage to be really like chilled out. Like, really mellow and lax and stuff. And I actually quite noticed them peaks and troughs with the mood and stuff. It goes literally from one extreme to the other, you know, uh on either side. So, I'd say, yeah, irritability, probably low mood, a little bit that comes into. And yeah, I'd say that's probably it. In terms of emotional symptoms.

0:15:43.250 --> 0:16:2.490
*Interviewer*
Yeah. And again, in terms of racing and you said you obviously haven't had, you've managed to be lucky avoid the very first few days of period when racing? But do they have any effect on racing or your prep for racing? Getting mentally prepared for racing. Obviously, if you chilled is probably quite good, but if you are in a more irritable and stuff does that have any effect?

0:16:3.460 --> 0:16:48.20
*P1*
Yeah, it does. I'm sort of I'm a bit of a perfectionist with things anyways, so I even have like as mad as it sounds. I have a bit of a spreadsheet for timings and stuff and I'm very strict with sticking to what time me alarm goes off, what time I'll eat a certain food, what time I'll then eat again and that sort of thing. So, I kind of try to alleviate stress in that way. So, whatever what's in my control. In a way, you know and timings and stuff to kind of not have as much stress with how irritable I am feeling on the day, but yeah it definitely sort of gets in the way. It's just sort of. I just need to be left to myself and sort things out, but I try my best to have as much as I can organise to take away any extra stress when I'm already feeling pretty stressed with things.

0:16:48.880 --> 0:16:56.430
*Interviewer*
Do you mention that to your team manager or with your teammates when you're feeling a bit like that, you just need to be left alone? Or do you just get on with it.

0:16:57.310 --> 0:17:35.40
*P1*
I think they can probably tell. I think I can. Uh, I'm. I'm pretty much straight at the point with things. I think I can be read quite well and if they just sort of ask, I would, just say it outright. Like, yeah, I think they would just sort of leave you leave it to get on with it really or kind of know how you feel and just know not to be winding you up or anything, you know? But yeah, I think they'll be happy for you to verbally tell them how you feel. And I think the prefer that to be honest. But I think I can be read quite well with, if you know how I'm feeling on the day off and I think they just leave you to it. Really everyone reacts different. Everyone prepares differently and you get sussed out quite quickly with things.

0:17:35.820 --> 0:17:49.720
*Interviewer*
Yes. And this kind of quite specific question, do you experience any changes I suppose in your core strength, coordination, mobility, or flexibility throughout the menstrual cycle? And if you do, do you make any modifications for that?

0:17:51.50 --> 0:19:27.680
*P1*
Probably something I've not really noted in terms of mobility and stuff throughout the cycle, or strength is probably, again, there's probably times where some sessions will feel harder than others. And I've just not really put two and two together. I think I'd I think if there is a phase in which I was in the menstrual phase and doing core work, I'd probably bring the reps down a little bit more, bring the weight down a little bit more just genuinely because I just don't feel like it. You know, I wouldn't really push past that. I think you can be more prone to injury, if you already know if you dialled in with how you feel with your body. And I think if you're pushed beyond that, I think some people can go a little bit too far with like, oh, no pain, no gain and stuff. But I think if you actually listen to your body, you become a lot less prone to injury. I don't know if there's any sort of connection with being more susceptible to niggles and stuff at certain types of the cycle. Just I wouldn't, probably wouldn't push beyond what I was feeling on the day and flexibility wise. Dunno again, haven't really tracked flexibility in different cycles, and I wouldn't say I notice it very well. I'm. I'm hypermobile, so I don't really have much problem with flexibility and the years today is when I feel quite stiff in that, or some muscles are got a little bit of a spasm in them, and I cannot really stretch them out very well. But again, it's not something I've previously tracked, so it's quite hard to comment on whether it would affect core strength or mobility, flexibility, and stuff.

0:19:28.420 --> 0:19:56.340
*Interviewer*
Yeah. Know that's fine. And you mentioned, I think you said that you track your cycle and, but you said you maybe don't mention any of this with your coach even though you've got a female coach. So, you don't necessarily share that to get any kind of training modified around it. Do you ever make? I don't know how you record your training with your coach. Do you ever make notes on training peaks if you have had symptoms or anything around that for your coach to see?

0:19:57.10 --> 0:21:28.50
*P1*
Uh, so on training peaks at I'm pretty the verbal with things I do comment a lot. I think communication in absolute key factor with the relationship between a coach and athlete. So, I tend to moan about absolutely everything else. I wouldn't specifically say in terms of that, like I think if I was just feeling rubbish, my comment would start off with just feeling a bit grim today, but there's no real questions as to why, she's obviously would just pick up on that a little bit. And you know, if I've got a virus, I'll always see if I've got a virus. But if I'm feeling poorly for whatever other reason, I think I would just feel, say, like, yeah, just not feeling, it really. And she doesn't really question it in a sense. I think she knows. I would say if it was anything worse than that, whether it was an injury. No, it's just, not really anything that's really been discussed. If she wanted to know about and asked, I think I'd feel a bit more open, but it's never been a topic of discussion, so I just don't. I've never felt the need to comment on it. I just feel it's a bit more the norm, you know, just probably very aware that these things happen, she’s a female herself, an athlete herself, a professional cyclist, so she'll have her. She'll be very aware of how it affects people and stuff, but it's never been the topic of conversation, for no real reason. It's not been avoided as such. It's just never actually been brought up, you know.

0:21:28.600 --> 0:21:47.750
*Interviewer*
Yeah, yeah, yeah. I think I mentioned in the pre interview question, and you mentioned it previously in terms of getting advice and learning more about the menstrual cycle. That's mainly been down to you doing research on the Internet and stuff you've not had any kind of guidance as an athlete from your team or anything like that.

0:21:48.940 --> 0:23:2.330
*P1*
No, no, never. I mean, people aren't. We've had riders turn up. They've travelled from Cornwall up to Northumberland to do a race and it ended up being their first day of their period and they couldn't even start. You know, they've done all that traveling to go up there and I guess everyone symptoms different and they're pretty happy to talk about it. And you know, your team manager can't force you to do anything. Like if you if it's that bad, it's that bad. You know it's, but I think our team manager is male as well and I don't think it would. It's really, I don't know. I think it's a generational thing. I don't know if this sounds weird, but with men in a generational thing, I think they feel it's a bit of a taboo or it's an embarrassing thing to talk about. Where's within females? I don't think they're really that bothered. You know, it's like or some people might be. Some people might be embarrassed, but less bothered, I guess. but it's not something that will be spoken about within the team. I think you would have to speak up and say if something I don't think they would ever ask you where you're at with things or if you feel a rubbish, then questions. Where are you at? With your cycle you know is. Could it be anything to do with that? Or again, male team managers and male DS’s. It's just not really topic of conversation.

0:23:3.430 --> 0:23:11.700
*Interviewer*
Yeah, if you had to. Would you feel comfortable talking to your DS or manager about it? If you say couldn't start, or race.

0:23:11.540 --> 0:24:0.310
*P1*
And I think I'm. I wouldn't be embarrassed to talk about it like I'm pretty happy to talk about it, but I probably wouldn't say that's why it was. I don't know why now that you've asked us that I've not thought about that. But if there was a reason, I don't know if I was on the spot, if I would just think of another reason not to, or whether I would just do it because I don't know if I would not start because of it, unless it was that bad. I don't know. It's a hard one. I think unless it happened, and I think I'd probably go along with it and just maybe just say I wasn’t feeling well. I don't know. It's funny, isn't it? It's a weird one like I'm. I'm not. It's. I don't know why. It's just I don't know if I would just maybe say it was something another reason, I don’t know.

0:24:1.300 --> 0:24:20.270
*Interviewer*
Obviously you said over kind of more recently you've done more research about menstrual cycle. Has that been prompted by - there's been a lot more discussion in the media, in some elite athletes like Dina Asher-Smith, Eilish McColgan talked about that. Has that prompted you to look or have other reasons prompted you to start doing more research?

0:24:20.670 --> 0:26:39.770
*P1*
But then I think I started looking into it. Umm, I don't know where the first time I saw it, but I did see the different phases being beneficial to different types of training and I thought well, you know we're getting, you've got your hormones there like, why am I not utilising and reaping rewards of just such a natural cycle? You know, hormones. You know it's. It seems silly not to and I started looking into a bit. Then I wanted to do it with the coach and stuff, and I have seen things and there's a, there's a book that I've read athlete called Lauren Fleshman. She's an American track runner and she sort of; it's called, that's what I think of the name of it. I can't remember the name of it. It's basically about females, sort of being brought up from a young age pre pubic and they're sort of like being sucked into, because if you do like anaerobic training before you hit puberty, you can actually reap rewards of it. And I think there's a real slippery slope with coaches and young girls trying to prevent them starting their periods because they can get so much more from their athletes at a younger age, and it's a bit about that. And it's a bit about how once the women started, then start their periods and become women, they would become worse of an athlete. And when she was younger, she was frightened to make for that transition to happen. Stuff shouldn't want to get slow as she wanted to win races and stuff and it's just been like I often read things like that. Like what women bring out? Because I think it's, it's often overshadowed a lot and I think it's a cycling especially is very male dominated sport, and you don't often hear about the female side of it. A lot of female cycling is not even televised and stuff, so I kind of make a point of looking into things and reading people's blogs and stuff. So, I think have been prompted by just looking at jus an interest myself, I don't think anyone specifically told us to look at it or I've heard anyone talking about it in particularly, I think I've just genuinely shown an interest and you know if there's a way to benefit your training or your performance legally. You know, I want to hear about it. You know, it's still. It's silly not to know about it.

0:26:40.480 --> 0:27:6.940
*Interviewer*
Yeah. Yeah, I think often this is where it's been approached from because I used to be a runner as well. Is around kind of what they used to call the female athlete triad or what they call REDS now? And yeah, that particularly endurance sport that it was seen as preferential not to have any periods and you would be a better endurance runner if that happens. And obviously there's a lot been a lot more talk about the negative impact that has on your body long term. So yeah.

0:27:7.490 --> 0:27:29.780
*P1*
Yeah, it is now. It's sort of coming out now because there were, there were doing it back then, seeing the rewards of it, whereas now you've got women that have went down the line of RED’S and stuff or eating disorders because they've been trying to not have their menstrual cycle and stuff and then the detrimental effects it’s had on their bones and their bodies and everything like that is it. They're at that age now where they can talk about it, you know.

0:27:30.50 --> 0:27:41.430
*Interviewer*
Yeah, I'm going to move on to the second part of the interview, but before I do, do you have anything else you'd like to add our own kind of periods menstrual cycle before we move on that you think you would like to mention that you haven't?

0:27:42.120 --> 0:27:43.140
*P1*
No, I don't think so.

0:27:44.360 --> 0:28:7.780
*Interviewer*
OK, so yeah, but total change tack. I think so this is more around kind of cycling, bike set up and yeah, kind of cycling injuries. I think from your pre interview questionnaire you just ride kind of male/unisex bikes. You haven't used any modification to kind of female specific bikes or saddles. Is that right?

0:28:8.520 --> 0:28:9.190
*P1*
That's correct, yeah.

0:28:9.640 --> 0:28:17.120
*Interviewer*
And when you bought those, did you make any modifications to suit your position? You know, like stems, crank lengths, handlebars, all that kind of.

0:28:16.880 --> 0:29:34.30
*P1*
Yeah, all of them. Yeah, yeah, all of them. So, I'm very pedantic about. I notice millimetres out on things and fits not being quite right and stuff, and I can spend hundreds of pounds on bike fits, and I can completely undo everything again, because it’s just not right. And so yeah, we sort of, we normally have a team bike. So we've went through different bikes, we've had Lapierre’s, Bianchi and stuff at the minute now and Trek. So, it's sort of you get given a frame, you don't really have much say in it. Luckily, I think the frames have been pretty good, but I always go for a very, very small frame and then I can make the modifications myself in terms of reach with stem length. But yeah, changed literally all of them for fit. So, I've sort of crank arm length. There's been a big one for me, especially with knee injury at the moment, so I've reduced that, and saddles something that I've struggled to get right, especially from a female perspective. I think you've got a really, really can get through hundreds of saddles and you know, it's still it's you'd probably know yourself. It's very difficult to find the right one in terms of comfort, mainly.

0:29:33.290 --> 0:29:37.620
*Interviewer*
So have you done that just on kind of trial-and-error basis in terms of choosing your saddle?

0:29:38.90 --> 0:30:40.200
*P1*
Yeah, sort of. Asked around before, I've getting recommendations from other female cyclists and seeing what they're using and yeah just buying some second-hand saddles, trying them, if they're no good, sell them on again. But yeah, tried different ones, even on a bike fit you get put on a couple of different saddles. I just know straight away. I'm literally like absolutely not. And then just female specific saddles that I have tried and in the kind of like have a little area in the saddle where it dips down for comfort reasons rather than the cutter wave, so it dips down, but I felt like I was being forced into that position. And again, I'm quite specific, I sit quite far on the nose of the saddle. And as you do in an aero position, you know, and it's the tilt, it's about getting the tilt right, sort of being in line with your pelvic tilt and being comfortable in aero position and stuff. And it's all minor tweaks to be comfortable and avoid injury or you know, sores and stuff, you know.

0:30:41.150 --> 0:30:44.450
*Interviewer*
So when you talk about tilt, do you have the nose tilted slightly down?

0:30:44.940 --> 0:30:47.180
*P1*
Yeah, quite a bit. Yeah, quite significantly.

0:30:48.130 --> 0:31:4.530
*Interviewer*
Yeah, because obviously the UCI, I think it's probably gonna be, it’s quite a while ago now - they changed their regs on saddle tilt to enable. Yeah, basically it was particularly around female riders to allow that offload on the front. In terms of, you mentioned crank length. So, what crank length do you ride? What have you changed it to?

0:31:6.330 --> 0:31:35.390
*P1*
Yeah so, I'm 165’s now and I was 170 and again that was just mainly for and the Patella mal-tracking. So obviously the longer the crank length was the more sort of movement there was my kneecap as it was coming back around, and stuff and it was just very uncomfortable. I've got quite a high cadence as well, so, the 165 sort of fitted me quite well, but I'm relatively new to them. I've had them for about six months.

0:31:35.850 --> 0:31:46.260
*Interviewer*
Yeah. And are you mainly? Do you pretty much always just ride a road bike? Do you have the same setup on that, or do you also ride TT bikes and other types of bike.

0:31:45.730 --> 0:32:7.730
*P1*
No, just road bikes. We used to have, uh, when we rode Lapierre, you'd have two of the same bikes. You'd have one that you'd race on and one as a training bike, but you try to keep things as similar as possible, so one of the bikes would go on the top of the car and you would have. Obviously, you want that to be as similar as possible.

0:32:10.680 --> 0:32:23.600
*Interviewer*
And so in terms of injuries you've suffered and then more about overuse, obviously you've had big problems with your knee. Have you had any other overuse injuries at lower back pain, hand numbness or tingling?

0:32:22.90 --> 0:33:37.340
*P1*
Yeah, currently got a bit. Currently got a bit of a niggle with the QL muscle at the moment and that is overuse. So yeah, down, I sort of had a bit of issues down my left-hand side. I don't know if it's just muscle recruitment or I'm still overcompensating slightly from the knee injury on the right-hand side, but my QL muscle tends to be too tight. Alot of the time, and I get a lot of issues sort of around the SI joint, my glute doesn't always fire up as well as I'd like to on that side. So, I fatigue quite easily down that side. And so yeah, there's a lot of tweaking with cleat position for that type of thing. And a lot of exercises, I'm doing off the bike just merely for stabilising., really, as opposed to strength work. And so, yeah, if injuries, I'd say that's an overuse injury, the QL muscles, tends to get quite a few aches and pains and it spasms and that. The tendonitis was the oven overuse injury. Umm, I don't think I've really got anything else other than tight down the left-hand side as an overuse.

0:33:38.80 --> 0:33:55.910
*Interviewer*
Yeah. And so have you. Have you suffered from saddle sores in the past and if you have where have you suffered them. If you would like, I've got a diagram to aid discussion. Or if you wanna just tell me, that's fine.

0:33:57.320 --> 0:34:31.300
*P1*
Yeah. So, I had one just before a race last year, so that was in September. So, before racing, I had to have my saddle nose really tilted down, training wise I couldn't even train on the bike I was having to swim and walk. Uh, just for it to settle down, but I went into that race with a saddle sore and with literally I was., if I was to do take my hands off the bars, I would have been sliding far too much down the saddle, but I had to have a tilted to alleviate any pressure.

0:34:34.80 --> 0:34:40.620
*Interviewer*
So how long did you have to have off the bike before the race to try and help it settle?

0:34:41.220 --> 0:35:7.850
*P1*
And I had a week off, I'd say 2 weeks before race I had a full week off and I'd say I had to swim and just in walk just to keep up sort of the aerobic base a little bit and then I had to ride again leading up to it a little bit. But like I say, the nose, the week leading up to it. I was riding but had to make a fair few changes to my saddle just to be able to sit down, you know.

0:35:9.390 --> 0:35:17.250
*Interviewer*
Apart from rest, was there anything else you did to try and treat it? Did you have to take painkillers, or did it get infected or anything like that?

0:35:15.790 --> 0:35:44.140
*P1*
I used a steroid cream on it. I didn't specifically go to the doctors for this sore, but my boyfriend has previously had sores before and it's the same cream and it's just sort of it. It gets rid of the inflammation, and if it's if there's any infection in it that it would sort of help that disperse. But I just use the steroid cream that we'd already had in the house.

0:35:45.540 --> 0:36:2.220
*Interviewer*
In terms of saddle sore and probably easy if I should be if I share my screen, I'll show you a picture. It’s a bit graphic.

0:36:0.920 --> 0:36:4.100
*P1*
So it's not just loading them.

0:36:2.490 --> 0:36:5.660
*Interviewer*
So it's on question 11,

0:36:9.720 --> 0:36:11.540
*P1*
Looking at that is graphic, isn't it?

0:36:12.280 --> 0:36:14.330
*Interviewer*
I just thought I would warn you.

0:36:13.970 --> 0:36:18.440
*P1*
I'm actually like zooming in because it's not little, but I'm like.

0:36:17.460 --> 0:36:23.890
*Interviewer*
So is it kind of like labia or upper thigh?

0:36:25.400 --> 0:36:29.110
*P1*
It was more like on the outside. Yeah. So, it was more like.

0:36:30.370 --> 0:36:31.370
*Interviewer*
The outer labia?

0:36:31.840 --> 0:36:34.970
*P1*
Further out than that, like. Yeah, like even more towards me.

0:36:31.380 --> 0:36:43.140
*Interviewer*

So more like top of the thigh. And was it the front? Was at the front end. Or was it more towards the sit bones?

0:36:45.310 --> 0:36:46.870
*P1*
Probably in the middle to be honest.

0:36:48.250 --> 0:36:53.570
*Interviewer*
And do you think that was do you think that was caused by chafing or just constant pressure?

0:36:55.620 --> 0:37:29.470
*P1*
It could possibly be chafing with where it was, so it would be like where your chammie pad would probably chafe slightly if it wasn't a very good pair of shorts or something. So, I think it's been a combination of both really. I think it's been pressure. So, a lot of hours in the saddle. Maybe’s not getting up out the saddle enough and stuff and just being uncomfortable. Maybe my shorts haven't been sat right or haven't put enough chammie cream on that day. So, I think it's been a combination of pressure and chafing and yeah, we're thinking of where it was location wise.

0:37:30.120 --> 0:37:34.580
*Interviewer*
Have you had similar saddle sores before, or was that the first time you have had a bad one?

0:37:35.210 --> 0:37:46.800
*P1*
Uh, I've had sort of small on sets of them, but nothing that's really restricted as much as that like that. That was probably the worst I've had. Like I couldn't, like I couldn't physically sit on the bike with that.

0:37:48.220 --> 0:37:59.270
*Interviewer*
Yeah. In terms of likelihood of developing saddle sores, I think you talked about there is it, do you think for you, it's like time in the saddle, and maybe combination of shorts and saddle in terms of where the chammie sits.

0:38:0.510 --> 0:38:43.870
*P1*
Yeah. I mean, I wear the same shorts, so it's, I don't tend to change different kit, like we've got our team kit and just it's pretty good. We've been lucky to get good kit and good chammie pads in them, and I think it's just been a combination of it. Just. Yeah, just, long hours in the saddle and maybe it's not been sat right on it on the properly. Maybe it's been a little bit a bit skewed me, me kit, or I say not used enough chammie cream that day and it's just been a combination of friction and pressure and or whether there's been a small sore there that previously wouldn't really affect us, but I've aggravated it significantly and it's ended up you know to the point where it was that bad.

0:38:46.310 --> 0:38:53.220
*Interviewer*
And when you suffered from the saddle sore, did you discuss this with your coach? I presume you maybe had to if you had to have time off the bike or.

0:38:54.650 --> 0:40:1.340
*P1*
So yeah, it was sort of a period in which I wasn't really with the coach. I just left a coach in May. I think it was, so this race was in September, so it was a bit of no man's land in between. So, it was mainly just discussing things with my boyfriend. So, he's actually helps used to help us quite a lot with train and stuff is very dialled in on that type of stuff. So, it was mainly a conversation weighing up what would be the benefits. So, I had to be the point where I could sit on a saddle for the race when needed to rest it and let it settle down. But I still needed to be doing something, so it was just a bit of a happy medium. So yeah, it was. It was up for discussion for sure, like if I got one now, I'd literally just have to say, like I cannot. I cannot ride my bike. I know she, my current coach has suffered with saddle sores previously, so it's not something you know it's a pretty well-known and injury, as such, within cycling, you know it's something that can, it can ruin people's races. It can ruin people's training. You know, it's quite a big deal really.

0:40:2.820 --> 0:40:7.760
*Interviewer*
Yeah. And after the race, did that make it worse? Did you have to take some time off following the race?

0:40:9.480 --> 0:40:27.640
*P1*
It was the last race of the season anyways, so it was, it was a week, at least a week off the bike completely, and anyway, so at least I had time for it to completely go. And it was just kind of persevering a little bit to get through a race with it. But yeah, had to make alterations to the saddle and stuff for that.

0:40:29.630 --> 0:40:41.660
*Interviewer*

I suppose since that happened, have you made any changes to try and avoid it happening again in terms of like bicycle set up, like type of shorts, chammie cream, physio if it's an imbalance type issue.

0:40:42.510 --> 0:40:57.450
*P1*
Yeah. So yeah, another bike fit and a different saddle. I've changed saddles again since and so yeah, both of those things really.

0:40:56.700 --> 0:41:1.630
*Interviewer*
OK, I'm pretty much everything on that. I've just got one last question. Are there any areas you would like research in regard to female cycling, menstrual cycle, optimizing training, bicycle setup, saddle choice, injuries? Anything around that you think we should be doing some research in?

0:41:18.780 --> 0:42:39.120
*P1*

I think I think the one that you currently doing sort of around the menstrual cycle in terms of the different phases is the one that is educational to both male and female athletes and coaches and team managers. So I think it's something that that definitely sorting needs to be looked into more and just to see how it affects people because like, I say, you could be doing something and you don't have the answers to why you feel rubbish and you start to look into things more and you kick yourself a bit about things like wondering why you're feeling that way. Whereas, I think if it was normalised that it you should be feeling that way, I think it it's a bit more than accepting thing and I think mentally that helps athletes because people do get a little bit too, you know, caught up with not being their best and performing their best all the time, especially young athletes as well. And also the phases and how they can benefit you. So, times in which you're producing more testosterone or estrogen or whatever and stuff. And what type of training you should be doing around that? I think people would be very, very keen to know that can benefit from a natural cycle. And so although it's research that already happens, I don't think enough of it happens and or it's sort of like.

0:42:39.120 --> 0:42:42.760
*Interviewer*
And there's not enough education as I suppose, yeah.

0:42:42.760--> 0:43:50.540
*P1*
Yeah, definitely. But yeah, I think I think in terms of that, that's probably nailed on. It's a type of subject. We have always been very interested in and like when I saw that you were, you were researching it and needed participants, like I think it's pretty vital. I don’t think there's enough gone into it, or it's relatively new, I guess, it's kind of something that's been bypassed in the in the past. And so I think it's, it's, it's in not only interesting I think it can help a lot of athletes out in again from male coaches have a bit of understanding instead of just generic training programs to actually tailor them to their athlete and everybody has different symptoms and you know, it's nothing's really you tailor everything else, you know, you sort of dial in on your nutrition, you do all this work around S&C and training on the road and mobility and stretching and sleeping and hydrating it. But then there's, like, vital parts that you you're missing out on which no one sort of speaking about. So, I think it's silly to get everything else right and just not be talking about something that's it's quite blatantly there, you know.

0:43:51.170 --> 0:43:56.430
*Interviewer*
OK, great. If you got anything else, you'd like to add before I stop the recording.

0:43:57.60 --> 0:43:58.40
*P1*
I don't think so, no.

0:43:58.710 --> 0:44.04.0
*Interviewer*
OK.

**P2 – interview transcript**

0:0:0.0 --> 0:0:7.90
*Interviewer*
So how long have you currently been cycling, and have you always been a cyclist?

0:0:8.260 --> 0:0:16.160
*P2*
Umm, probably I started with my bike in 2004. Yeah, so 20 years.

0:0:19.520 --> 0:0:25.40
*Interviewer*
And how is training going at the minute? Are you planning to race this season?

0:0:25.50 --> 0:0:42.60
*P2*
So training has been going really well. But it's mainly indoors, lots of racing indoors and working on the FTP and five minute power and things like that. But didn't start with that. That's more of a recent thing, over the last year.

0:0:43.420 --> 0:0:47.760
*Interviewer*
And so, yeah, do you have any kind of goals or targets for this season?

0:0:48.970 --> 0:0:52.680
*P2*
Well, it was to get to A category on Zwift.

0:0:52.960 --> 0:0:54.220
*Interviewer*
OK and oh wow.

0:0:55.210 --> 0:1:20.390
*P2*
Fake. I'm not really very good at all, and so that was the goal. And then I could buy a new road bike, but at the moment I don't know. I've got some duathlons entered. So, I'd quite like to be a bit faster on the bike part, one of the consequences of that the running has deteriorated.

0:1:22.560 --> 0:1:33.790
*Interviewer*
The trade off with duathlon and triathlon which I used to find a massive challenge to balance. OK. So, moving on to the menstrual cycle, how old were you when you first started getting periods, was it 11? What was it like for you? Do you get any education around that time?

0:1:34.540 --> 0:1:42.930
*P2*
A little bit at school, it was an all-girls school, so bit there, but not really very much.

0:1:42.940 --> 0:1:45.890
*Interviewer*
Were you doing sport at that time? Did it have any effect on that?

0:1:46.580 --> 0:2:3.160
*P2*
Didn't notice it though. I didn't do sport, but I kind of didn't. So, I did lots of hockey and netball and that I used to avoid things like cross country.

0:2:3.240 --> 0:2:9.420
*Interviewer*
And as you got older, has your menstrual cycle stayed relatively similar or have you experience any changes?

0:2:9.890 --> 0:2:40.340
*P2*
I was on the pill for quite a long time, thinking that it was just so easy to get pregnant and obviously I would fall pregnant and then realise it wasn't. So, I guess it was. It was all quite fake from, probably the age of 25 to 32. It was all controlled by the combined pill. So, I didn't really know, and then it took a while to go back to normal cycles when I came off that.

0:2:40.390 --> 0:2:48.830
*Interviewer*
Did you get any symptoms when you're on the combined pill or side effects.

0:2:48.840 --> 0:2:54.570
*P2*
I did get bits of cramping. It wasn't actually terrible. Yeah, it's only realised when it comes back how bad it can be.

0:2:56.120 --> 0:3:12.890
*Interviewer*
And when you obviously said once you came off the pill it took a while for your cycle for turn to normal, and once you're back on a more regular cycle did that that have any effect on you as an athlete, on your training.

0:3:13.220 --> 0:3:35.200
*P2*
Well probably wasn't doing as much because I was basically trying to get pregnant, and I suffered quite a few miscarriages while I was doing sport. So therefore, I stopped doing cycling and running. I just swam. So yeah, it's quite hard to tell and I wasn't doing things at the level that I would have been doing, thinking I want to have kids.

0:3:35.250 --> 0:3:38.880
*Interviewer*
So what's it like now? I think you did mention you have some symptoms on.

0:3:38.890 --> 0:4:12.390
*P2*
Yeah, and I don't know if it's because I'm what I'm trying to make myself do is a bit harder. Yeah, we do this racing series, this racing series I can literally every 4th Tuesday I am absolutely crap. And you know, just I'm like, what's not. And then I get. Oh, then it clicks and I'm like that's why I'm feeling the fatigue while I've got a bit of cramp, a bit of nauseous and but yeah, when it was on pretty much 28 days cycle, it always fell on the Tuesday, which is the same day as the race.

0:4:13.20 --> 0:4:18.50
*Interviewer*
So that is day one of your period, so is that the worst day for your symptoms I think so?

0:4:18.100 --> 0:4:21.820
*P2*
Yeah, that's what I've started to pick up. So that's why I've had to be a bit more strategic. When I look at can I not do that one or I might miss that one.

0:4:21.830 --> 0:4:30.460
*Interviewer*
Because do you track your cycle?

0:4:30.470 --> 0:5:2.40
*P2*
My Garmin does. When I keep getting these horrific messages, like when you, feel like you feel OK, oh, your period is due now. And yeah, it does track it, but I don't track the symptoms on it, but I think I'm, with knowing how I started to feel in races and predicting that I'm a bit more mindful of it now than I've probably have been. Yeah, I probably wouldn't have, you know, have done race every now and again for running or duathlon but because this is kind of like such a weekly thing, I'm just a bit more aware of it.

0:5:2.290 --> 0:5:9.100
*Interviewer*
Yeah. And in terms of symptoms, what physical symptoms are getting mentioned cramps and absolute give you pain as well?

0:5:9.110 --> 0:5:35.330
*P2*
Yeah, I feel like I have done a marathon. Yeah, like the quads. So, everything's always in my quads where I feel things. They just feel like someone's gotten a baseball bat and battered them around a bit and they don't wanna play. My heart rate does go a little bit higher than it should. I kind of go up a zone when I don't feel, I'm not doing that much. Yeah, and I think my husband would be say I am a bit ratty.

0:5:35.440 --> 0:5:39.920
*Interviewer*
Yeah, I was gonna ask if you have any of the more

0:5:40.50 --> 0:6:14.940
*P2*
More probably a little bit before more pre? Yeah, I get a bit kind of what I could just cry. Quite sure. Not really cry person, but yeah. And normally I can control what I eat, but I'm not sure I'm like, oh my god just give me sugar. Yeah, that doesn't, like no control like the day before. I just want to chocolate and get Nutella by the spoon.

0:6:8.170 --> 0:6:14.940
*Interviewer*
Do you consider yourself to have heavy periods or say fairly?

0:6:18.130 --> 0:6:19.980
*P2*
Probably just moderate, I would say.

0:6:19.990 --> 0:6:30.210
*Interviewer*
Yeah, is that a consideration for like if you are on training rides in terms of needing a break for toilets change sanitary protection or is that not really an issue for you?

0:6:32.330 --> 0:6:32.630
*P2*
OK.

0:6:35.110 --> 0:6:47.940
*Interviewer*
So you don't really need to. You don't feel apart from feeling a bit rubbish when you race you don't have to modify training around that, and when you go out.

0:6:47.950 --> 0:6:50.340
*P2*No, I'll probably would not go out and not do long rides because that would be my mind. I probably it's just easier to do it at home, yeah.

0:6:53.180 --> 0:6:55.210
*Interviewer*
Do you need to take any pain medication?

0:6:55.220 --> 0:6:57.70
*P2*
Sometimes I take some paracetamol.

0:6:58.940 --> 0:7:14.770
*Interviewer*
This is another question based around some of the previous research done. Do you ever experience any changes I suppose in your core strength, coordination, mobility, or flexibility throughout the menstrual cycle? Does this impact on your training?

0:7:14.770 --> 0:7:14.770

*P2*

Not that I know of.

0:7:14.860 --> 0:7:37.390
*Interviewer*
So you don't notice any like particularly, I don't know whether you sit in more aero position, you don't know notice it is more challenging.

0:7:37.400 --> 0:7:54.990
*P2*

I just wouldn't sit in the aero, if yeah, probably subconsciously would adapt. When I'm tired which can be at the same time, that I do feel a bit less confident on the like taking corners and stuff like that. And I start to doubt that I can do it and I might click out a bit more. I feel like if I'm feeling a bit ropey and it could be because I'm just tired, or it could be because I'm that ropey because of the period. Yeah, but yeah, I feel like, oh, my goodness, I could take that corner. Why have I not taken it very well? A bit of stuffed out sometimes.

0:7:55.0 --> 0:8:10.420
*Interviewer*
And in terms of what your menstrual cycling training, do you kind of just manage it now, by maybe slightly modifying when you're gonna race, if you know it's gonna fall on day one on Zwift race. Do you anything else to manage your training on competition around it?

0:8:11.910 --> 0:8:19.980
*P2*
And yeah, that that's about it really or just you make sure that they've got an extra person in the team, so if I do have to drop out, it isn't like a drastic thing.

0:8:21.130 --> 0:8:23.100
*Interviewer*
Would you let them know that's the reason why, or would you just say?

0:8:23.110 --> 0:8:41.690
*P2*
If it was the woman that I was training with I would, but when I'm racing with the men, but I just …. yeah. No. Well, I did yesterday say something to them, and they were like oh right, that that that's fine. So, I'm just gonna blame being female that can't hit these watts and then you can't say that I'm like, oh, I think I can.

0:8:46.450 --> 0:8:58.790
*Interviewer*
Have you as a female athlete have you ever had any like kind of information around the menstrual cycle or how it affects you? Have you ever had a coach that's giving you advice?

0:8:58.800 --> 0:9:20.710
*P2*
So, no, yeah, I think it's been quite interesting with the athletic scene that it's been talked about a lot more, isn't it? And I think that's raising the profile of it. When they were cramping and things like that, the sprinters and yeah, I was like, oh, that's quite interesting. So, then I did read a bit more about it. I thought, well, if they're elite athletes and getting effects, then of course I'm going to get effects.

0:9:21.400 --> 0:9:28.880
*Interviewer*
Yeah, because that was one of my questions. Have you been aware of the raised profile in the media?

0:9:28.890 --> 0:9:29.500
*P2*
Yeah, I think about speaking about it a lot more, aren't they?

0:9:29.710 --> 0:9:48.700
*Interviewer*
Yeah, which is a good thing. I think that's all the questions on menstrual cycle. If you've got anything else, you'd like to add or talk about before we move on?

0:9:48.710 --> 0:10:4.200
*P2*
No, I mean, I'm just probably been quite oblivious to until I started to expect a bit more from my body, really, I think apart from the whole trying to get pregnant thing and then trying to try a bit harder and then realizing that, oh, it's a bit unpredictable, that I can't be consistent, you know, and I think oh why is that? And then it's made me think. Yeah.

0:10:4.750 --> 0:10:43.460
*Interviewer*
No, I think it's ridiculous how like I've learned quite a lot about 30s about it. You're like, why are we not taught this as part of school apart from like, the getting pregnant part. The part about the symptoms and the phases and how that affects you. So, moving onto the bicycle equipment and injuries. I think when you shared your pre interview questionnaire, you had a mix of women’s specific and kind of men's bikes. In terms of your bikes, have you made any modifications to suit your setup or have you kind of just got them how they came?

0:10:43.570 --> 0:10:49.700
*P2*
No, so I had a bike setup with Nev Martin on my road bike. So, my old road bike and then and we just use those measurements on everything else.

0:10:51.150 --> 0:10:54.20
*Interviewer*
So in terms of change and stuff. Have you changed things like crank length, stem?

0:10:54.30 --> 0:11:16.420
*P2*
Both, all my bikes have like got narrower handlebars, female specific seats and swishy seats, and even got a gel seat cover on the wattbike because that does help, but that that's probably about it. And I've got a brand new bike. Female specific bike, which has been outside once.

0:11:23.120 --> 0:11:32.480
*Interviewer*
Have you or do you currently suffer from any cycling overuse injuries, so lower back pain, knee pain, saddle pain, foot pain or hands nerve pain?

0:11:33.230 --> 0:12:15.450
*P2*
It's quite hard to tell because I have quite a lot of niggles but I'm not sure if they're running niggles. Get made worse on the bike or vice versa, so I have very tight hamstrings and quite weak glutes, and at the moment I've got chronic, got chronic chondromalacia in my right knee. Yeah, I don't really have that much cartilage left the end of the. So that was from lots of running in my 20s. And so that was all worked up and scanned and everything. So, I'm just trying to keep that at bay. And it's OKish at the moment.

0:12:16.110 --> 0:12:23.190
*Interviewer*
Have you modified any of your position specifically for like your tight hamstrings or your knee, or?

0:12:23.200 --> 0:12:31.370
*P2*
No, I just went for that set up because it, yeah, he's very, very thorough. And he could see that I wasn't the most flexible, I think.

0:12:31.980 --> 0:12:34.250
*Interviewer*
So found he adapted your position.

0:12:34.540 --> 0:12:36.160
*P2*
Yeah.

0:12:36.660 --> 0:12:44.930
*Interviewer*
OK, great. In terms of your saddle and do you always ride a women's specific saddle, or do you have a mix?

0:12:45.240 --> 0:12:46.70
*P2*
I think so.

0:12:46.180 --> 0:12:49.160
*Interviewer*
Do you have the same saddle on all bikes? Or do you?

0:12:51.680 --> 0:13:5.60
*P2*
Umm, I have no idea what that one is. That might even be the one that they came with. To honest that feels alright, but I've got the Selle Italia on the road bikes.

0:13:5.850 --> 0:13:7.600
*Interviewer*
Is that one with a cut out?

0:13:7.750 --> 0:13:23.810
*P2*
Yeah. And I think I've got that on the on the wattbike as well. I bought the same one and put a gel seat cover on anyway, and just for extra since I was doing a lot longer on the wattbike than before.

0:13:23.880 --> 0:13:34.840
*Interviewer*
Do you have a TT bike? Or do you not ride TT? Would be ever ride in aero position on road bike or like the bars? Or do you just ride on the drops?

0:13:34.920 --> 0:13:40.20
*P2*
On the drops. But on the wattbike bike I will go into aero when I don't wanna look at the screen.

0:13:40.30 --> 0:13:45.310
*Interviewer*
Yeah. Do you find any difference in saddle comfort moving between the positions?

0:13:47.290 --> 0:13:56.150
*P2*
It does feel different. Yeah, I feel like I can't get as much power out on the wattbikes when you're in aero.

0:13:56.430 --> 0:14:3.660
*Interviewer*
You've mentioned you put a gel cover on your wattbike is that? Is that one of the times when it gets the most uncomfortable, the kind of longer.

0:14:4.130 --> 0:14:7.860
*P2*
Yes, because it's got no movement.

*Interviewer*

Do you'd all get out at all?

0:14:8.710 --> 0:14:21.870
*P2*
I try and get up and down, but when it's when I was like doing sort of 3.5 hours on it, it was getting a bit kind of like, yeah, sitting bothered. So, I just thought it was an extra thing to help. Perhaps.

0:14:22.900 --> 0:14:24.820
*Interviewer*
Have you ever suffered from saddle sores?

0:14:27.150 --> 0:14:45.660
*P2*
And I don't know what saddles. I don't know. I have dodgy skin, especially this time of year. My skins usually quite knackered, so I have to put lots of sudocrem round my legs. So, I am not sure if they're sort of saddle sores as such.

0:14:45.670 --> 0:15:15.140
*Interviewer*
Quite broad term, so it can be anything, can be pressure related, kind of chafing related. Some people develop like little cysts or boils or like, yeah, broken skin and, yeah, swelling, or any kind of numbness, I suppose as well for the sit bones, upper thigh, vulva region, the whole lot for female cyclists. So, do you ever suffer?

0:15:16.880 --> 0:16:0.630
*P2*
So I think I'd probably stop sometimes; it just gets uncomfortable. Yeah, especially those 3.5 hours on the wattbike. So, I can feel that that's that and it’s kind of an ache afterwards as well and it's quite uncomfortable, even like sitting down after getting things. But the skin at the top I think it's kind of like almost like friction this time of year, it gets from the cold quite bad, I think, from doing the bike, changing bikes, changing clothes can't help, showering in between, but then maybe I wouldn't shower necessarily at work, because then they'll be like 4 showers a day. And yeah, I am like I can't.

0:16:0.640 --> 0:16:4.160
*Interviewer*
So have you done anything apart from kind of limiting your ride hours if you start feeling comfortable and using the geography, have you done anything else to kind of help?

0:16:11.340 --> 0:16:23.660
*P2*
I have got some anti chafing cream chammie stuff that maybe I would do if I was doing it with a long bike ride. I would put that on, and I do use quite a lot of sudocrem yeah, to try and help heal the horrible skin here.

0:16:24.300 --> 0:16:32.330
*Interviewer*
OK, has saddle discomfort ever affected your ability to train and compete?

0:16:33.430 --> 0:16:38.800
*P2*
I don’t really go that far.

0:16:38.910 --> 0:16:39.740
*Interviewer*

So, it’s never made you have to take a break off the bike or modify training?

0:16:38.910 --> 0:17:11.130
*P2*
No, I guess it was just it. It's one of those things. There's quite a few more group that I cycle with “Oh yeah, we're gonna do like this ridiculous, we're gonna go and do Everest”. And I'm like, yeah, no, I can see that being really in a couple of have lots of time. But yeah, it would. It's one of the reasons I wouldn't want to do that because that would be a long time in the saddle. Yeah, I would be really quite sore. Yeah, not just during that ride, but potentially for days and days afterwards. But yeah, I don't put myself there. So, I guess it's a pre-empting rather than. It's the fear.

0:17:14.620 --> 0:17:33.250
*Interviewer*
I think that's all my questions. Sorry, one more question in terms of kind of anything regarding female cycling, so menstrual cycle, training, bike set up, injuries particularly related to the female cyclist. Are there any areas you think we need to do more research or that you would like researching?

0:17:35.430 --> 0:18:14.320
*P2*
I guess there's something that always crops up is maybe it's more from running, but I guess it applies to cycling as well. Is that this kind of like the weak glutes, weak core, weak kind of pelvic area in female seems a lot more just from taking people a lot more predominant in females than it is in males. And all the niggles that go along with that, like the knee pain, the IT bands, the piriformis, all the injuries. I literally come out one and I go into the next and stuff and it's all related to weak core and glutes. I wonder whether there is more that could be done about that, and more preventative rather than treatment.

0:18:15.70 --> 0:18:20.950
*Interviewer*
So kind of programmes, to design programmes and test them to kind of improve this core area particularly in females.

0:18:20.960 --> 0:18:23.240
*P2*
Yeah, because it just seems so common.

0:18:25.150 --> 0:18:30.240
*Interviewer*
Have you got anything else?

0:18:32.620 --> 0:19:48.180
*P2*
Well, actually someone at Newcastle is doing a project recently on how through the cycle I think how your immune system dipped. And how you're more prone to things at different age. I think that's quite interesting because that's what I do - immunology. So that would be quite interesting to know. I think physiology - I'd really love all the data is based on male figures and all these heart rate zones and zones of training. It all seems very male and one of the guys that I cycle is that like, Oh, yeah, I don't like that's not working, but the numbers don't match up. Yeah, that would be quite interesting to, yeah, to look at more closely because that's like, you know, I can't do that. I don't wanna say because I'm a girl because I don't know n equals one, or is it just me, or is it? Why can't I hit the really high powers that they not that ridiculously high, but I can't. Yeah, I can't hit over 300 watts. And they're like, yeah, let's go 300. And I'm like I have a mental block. I can't. I think my body can't do it very well. But when you look at our profiles and that they're really similar, but how come you can go that much? And I can't do that. So, there's something there, I don’t know what.

0:19:50.210 --> 0:20:20.840
*Interviewer*
There's definitely sex differences, particularly in the top end power. Males are just more powerful and that is, and I think that is something that, well, my PhD students trying to address with the sex differences so it's not all based on training females like and small, less powerful men, because when we don't have the same profile, we are much more fatigue resistant than men but we can't max out the same power as men do though.

0:20:21.810 --> 0:20:26.20
*P2*
Which is why when you race men it's just a completely different experience. I'm like, oh, my God, it’s horrible.

0:20:29.250 --> 0:20:35.210
*Interviewer*
Have you got anything else you'd like to add before I switch off the recording?

0:20:36.10 --> 0:20:36.880
*Interviewer*
Ok, thank you very much.

**P3 - Interview transcript**

0:0:0.0 -0:0:12.250
*Interviewer*
So just a bit of background about you and cycling. How long have you been cycling, and what events do you do, or type of riding?

0:0:13.90 -> 0:1:29.700
*P3*
So I started cycling probably about six or seven years ago. So, I think at first the free time and exercise, and keeping fit was probably the main reason I started. Then in terms of events that I've been doing, I've done a few sportives and I've taken part in the occasional time trial. So, I do do, so last year I started doing a lot more the club time trials rather than any of the open time trials which are organised by CTT, although I have done the odd one of those. This year I'm planning to probably stick to that again to be honest I will aim for the club time trials again, maybe one or two CTT. Then in terms of events I’m doing the Fred Whitton this year which I’ve not done before, so I’m trying to get my head into training for that at the minute as it’s not far away.

0:1:32.680 --> 0:1:38.580
*Interviewer*
Do you mainly, is it all road biking or do you do a bit of mountain biking or anything like that?

0:1:37.80 --> 0:1:47.710
*P3*
Yeah, it's road biking. I’ve tried gravel riding, but it just didn't click so I stick to the road.

0:1:49.360 --> 0:2:05.320
*Interviewer*
OK, that's great. So, moving onto the menstrual cycle, thinking about when you started having your period, what was it like for you? How old were you? Did you get any sort of education or advice around that time?

0:2:06.0 --> 0:2:45.250
*P3*
So I think I was 14, I felt like I was fairly late compared to my peers. I was fine, I think I probably knew, everything had been covered at school and had the talk from my mum so I kind of knew. I do remember the first time, obviously in hindsight it was quite bad period pains, I had no idea what it was, I was just why, it was so painful. But apart from that it was fine.

0:2:45.500 --> 0:2:49.740
*Interviewer*
Were you playing sport at that time did starting your period have any effect on playing sport?

0:2:50.410 --> 0:3:11.180
*P3*
No, I mean I have to say I wasn't particularly sporty growing up. It was just school PE, but I didn’t notice any massive impact. I suppose the biggest impact I had was swimming because it’s always difficult to know what sanitary protection to use and stuff so.

0:3:12.200 --> 0:3:28.40
*Interviewer*
As you've got older, has your menstrual cycle changed? I think you mentioned being on hormonal contraceptive for a while, the pill. As you’ve got older and changed between different types, has that had any effect on your symptoms?

0:3:29.910 --> 0:4:21.450
*P3*
Yes, it has changed. So, I would say from starting right up through my 20’s I was always really regular and I suppose going on the pill when I was maybe about 20 kept it regulated. But then when I had my first daughter at 29, then after that everything went crazy in terms of my cycles, it was never the same again. I never went back on hormonal contraceptive after the kids. It’s gone from very few and far between to a bit regular and then it goes away again, so I don’t know what's going on there.

0:4:18.300 --> 0:4:27.840
*Interviewer*
So you mentioned that your periods are quite irregular. So, do you track that or try to do anything to figure out what's going on? Or do you just accept it's going come when it's going to come?

0:4:28.460 --> 0:5:10.520
*P3*
I do track it on the Apple Health app just so that I know when the last one was and things like that. I do recognize the symptoms, so I know when it’ll be coming even if it's been a while since the last one. I do recognise that certain symptoms will probably mean that it's only like a couple of weeks off. But I don’t know, in terms of having it investigated, I think I did have various blood tests, so on and so forth but it all fine, and it was just one of those things apparently.

0:5:12.590 --> 0:5:30.280
*Interviewer*
On the form as well, you mentioned that you suffer some symptoms from your periods. How would you rate those? How bad are the physical symptoms? Do they effect you?

0:5:28.140 --> 0:6:23.100
*P3*
What I find is that the worst symptoms are premenstrual. So, when I'm actually on my period itself, I don't feel too bad. It's maybe the 10 days to week leading up to it, just really bad, really bad cramps and lots of low mood and things. Just in general, in terms of training just a lot more tired at that point. So, lots of lethargy and not really and not feeling as strong when you're when you're out and then normally on day two of my actual period I am like yeah, I’ve got my energy back, I'm going to go again. I don't know how common it is that way around but that's certainly the way that I experience it.

0:6:24.840 --> 0:6:35.900
*Interviewer*
Yeah, I think it's mixed. So, you obviously feel really tired and not up for it. Do you modify your training? Do you like reduce it a bit during that period?

0:6:36.560 --> 0:7:18.530
*P3*
I do actually, I do tend to. I used to find, not so bad with cycling, but when I used to run, which I have stopped running, in the past kind of year or so I couldn’t actually run in those times with stomach cramps and everything. It was just a no go with running but with cycling because you’ve not got that same impact I don’t think around your abdomen, when your running you have impact going up through your body, so but with cycling it not that, I can still cycle it's just less and I taper a bit.

0:7:18.760 --> 0:7:37.510
*Interviewer*
Obviously you said once your period starts normally by about day two you’re not feeling as bad, you feel like you got some energy back. Do you ever have to modify what you do training in terms of going out on the road? Is that a concern about needing to have a toilet stop or whatever for sanitary protection or anything like that?

0:7:37.500 --> 0:8:20.850
*P3*
It is. I just try and hope for the best really. I only get kind of a couple of days that it’s really heavy. I did find out one point that the pads in cycling tights are not that absorbent when I was riding a white bike, it was in lockdown there was nobody there, so nobody saw. So yeah, I've kind of just hoped for the best, and I might have missed a long ride because I wasn't sure I’d have been able to like change on time.

0:8:20.800 --> 0:8:39.410
*Interviewer*
And one question, I think you mentioned it, it relates a bit to what you felt about when you were running on your premenstrual symptoms and period with cramps. Do you feel like you've got any changes in your core strength, coordination or mobility, flexibility around any points in your menstrual cycle?

0:8:40.40 --> 0:9:12.790
*P3*
Yeah, I think it's more my back really. I think you feel if you try and do too much, it feels like putting pressure on your back. Because alongside the cycling, I do go to the gym as well, so I sort of modify what I'd do in the in the gym and maybe won't go to a particular class because I think it would be too heavy around my core and then put the pressure on my back and I don't want to risk any straining on that.

0:9:13.920 --> 0:9:19.300
*Interviewer*
In terms of bike set up, are you always on a road bike set at fairly similar? You don't have a TT bike, is that right?

0:9:19.650 --> 0:9:22.400
*P3*
Yeah, I'm always on the road bike.

0:9:20.140 --> 0:9:30.630
*Interviewer*
Do you ever ride one or the other in terms of position for comfort? Sometimes, like winter bikes are a more relaxed setup, or does that not come into consideration?

0:9:32.440 --> 0:10:41.670
*P3*
I find them probably all equally as comfortable. But like you say, the one that use for commuting is a bit more relaxed and then the other two I think they do have pretty much the same setup. Originally the bike that I use in summer, which is what I would be doing the TT’s on, that was set up quite a bit more aggressively. But I did go for bike fitting, I was realising I’d spent all winter fine which was when I was riding my commuter bike but then suddenly in summer, I started getting loads of hip pain and thought ok, maybe it’s the bike set up because that was the only difference. So, I did go for a bike fit on that one and it’s a bit more relaxed now. It’s still not quite as relaxed as the other one but that has helped. I've got another one which I use all year round and it’s actually the bike I’ve got set up to do the hill climb for the Fred. It's got the really easy gearing on so kind of and that set up pretty much the same as my the one that will do the TT's on.

0:10:40.820 --> 0:10:49.790
*Interviewer*
In terms of like being a female athlete, have you ever had any specific advice around menstrual cycle, periods on this topic?

0:10:50.590 --> 0:11:10.950
*P3*
Not personally, no. Just sometimes read about it online. You know, if you find advice and you pick up like magazine Runners World or something has got an article on about training, but I have not had any kind of specific personal advice.

0:11:12.110 --> 0:11:15.240
*Interviewer*
Are you aware about the kind of increased discussion I suppose in the media on periods with female athletes. Have you seen any of those articles, maybe with Dina Asher-Smith or Eilish McColgan?

0:11:21.850 --> 0:11:45.830
*P3*
Yeah, I have actually, you do notice that a lot more, I think that has changed, hasn't it? I think people are a lot more open and speaking about it and recognition that it's not necessarily healthy for an athlete to overtrain so much and that they impact on their cycle. Yes, I have seen things like that.

0:11:46.100 --> 0:11:51.610
*Interviewer*
In terms of like comfort of having conversations around the menstrual cycle, do you ever talk to anybody about it? Would you talk to other riders or teammates?

0:11:56.360 --> 0:12:18.330
*P3*
Yeah. Sometimes. It would obviously depend on who, I'll probably say more closer friends rather than just generally speaking about it like in a middle of a club ride like how you getting on.

0:12:18.450 --> 0:12:21.550
*Interviewer*
That's all my questions around the kind of around the menstrual cycle. Did you have anything else you'd like to add or think I've missed?

0:12:32.540 --> 0:12:34.10
*P3*
No, I don’t think so.

0:12:39.200 --> 0:12:57.940
*Interviewer*
OK, so next section is around kind of bike equipment and injuries. I think from looking your interview questionnaire you've got is it two women specific bikes and then one I think it’s your commuter that's not women’s specific. Is that right? Or is your Ridley not women’s specific.

0:12:58.810 --> 0:13:15.580
*P3*
I don't think the Ridley is specific. I think they've called it women's version, but it's actually got exactly the same geometry as the men's version, just they make it an extra small way that they might not do for the for the men.

0:13:15.210 --> 0:13:21.790
*Interviewer*
So you if you've got two male bikes, have you made any modifications from those since you bought them to fit your setup.

0:13:22.570 --> 0:14:54.730
*P3*
Well, the Ridley was built from scratch, so I got the frame and husband kindly put it together for me. But the other two, they were bought and to say, the Merida I use for commuting, that is very much a women specific bike and that was the first bike that I got when I first got into to cycling and so I haven't in terms of setup, I don't think I made any changes to it. My other one I bought it from Cycle Art in Prudhoe and I'm not actually sure what bike it is because they have their, they’ve got this cycle brand. So, it's where they get frames from certain manufacturers, but then they rebrand them, so I’ve got this bike but I'm not quite sure what actually what the real model is. I think it might be Genesis, but apart from that I don't know what frame model it would be, but it was pretty much the same frame size as the Ridley. I've been to Cycle Art for the bike fits, and they have my details so when I bought it from them, they just set it up identically to how the Ridley was setup.

0:14:55.20 --> 0:15:00.510
*Interviewer*
In terms of your three bikes, are they comfortable? Or do you still think you could improve setup on them?

0:15:01.690 --> 0:15:24.130
*P3*
I think possibly I could improve setup on the two that are the same, as I do sometimes get pins and needles in my hands and my arm bit but it's not every time. It's one of those things I keep meaning to work out and have a look and sort it out, but then don't.

0:15:24.380 --> 0:15:30.330
*Interviewer*
Is that brought on by length of ride or does it not have a pattern?

0:15:32.570 --> 0:15:42.840
*P3*
Yeah, it will be after a certain amount of time, maybe an hour or so, and then it might come and go during the ride.

0:15:42.410 --> 0:15:48.290
*Interviewer*
Have you had any other overuse injuries so low back pain, knee pain, foot pain, saddle pain?

0:15:47.910 --> 0:16:23.620
*P3*
It was mainly that hip pain that I mentioned before, before I had the bike fit. I think, what did they say that was for, I can’t remember why, I want to say crank lengths, but I didn't think it was as I didn’t change the cranks, so that can’t have been it, so I’m not sure. It could have been seat height or angle or saddle angle or something.

0:16:25.910 --> 0:16:31.910
*Interviewer*
So are you saying this result of the hip pain, you did have a bike fit and then they modified position to try and help with that?

0:16:31.270 --> 0:16:45.480
*P3*
Yeah, I've not had that pain since. I think in terms of cycling probably the only thing, the is the pins and needles and numbness I need to figure out, but otherwise, yeah, that's the only thing.

0:16:48.50 --> 0:16:54.210
*Interviewer*
In terms of saddle, I think that you ride different saddles on your bikes.

0:16:54.550 --> 0:16:59.500
*P3*
Yeah, randomly three different ones.

0:17:00.840 --> 0:17:07.170
*Interviewer*
Is that like a conscious decision or is it just what was on them, and you've just been OK riding them and you've kept them the same?

0:17:50.170 --> 0:17:55.180
*P3*
So the Selle Italia one was the one that came on the Cycle Art bike and that was something I didn’t change and is probably most comfortable and it’s probably the most basic out of the three. Then I bought the other two as well, I think I have, I don't know why I’ve got different ones. I think I probably got one of them I got and thought it’s fine, but it's not amazing, so I'll try a different one, so I got saddle for to that bike and then again it was fine but not amazing, but I generally think that this Selle Italia one was, it's the most comfy.

0:17:56.760 --> 0:17:58.300
*Interviewer*
Is that a one with a cut out through the middle.

0:17:58.890 --> 0:18:01.30
*P3*
It's not fully cut out; it's got like a dent.

0:18:04.280 --> 0:18:05.840
*Interviewer*
Is that a women's specific one?

0:18:3.440 --> 0:18:28.170
*P3*
I don't know. I don't think it is. Because I noticed my daughter's cross bike, which is a Ridley, which we got from Wiggle and that's not women specific, but that came with the same saddle as I've got. So, I doubt it's the women's specific one.

0:18:26.710 --> 0:18:37.0
*Interviewer*
And following on from that, have you ever suffered from saddle sores when you've been out cycling so ranging just from like pain in sit bones to like chafing, pressure, cysts or anything like that?

0:18:38.710 --> 0:18:57.990
*P3*
Yeah, I do fairly often I'll find that I do get kind of spotting, I don’t really get any pressure sort of pain or any hip pain or pelvic pain, but it's more kind of skin issues.

0:18:57.360 --> 0:19:1.770
*Interviewer*
So like breaking the surface of the skin it?

0:19:1.190 --> 0:19:2.430
*P3*
Yeah, yeah.

0:19:3.920 --> 0:19:11.860
*Interviewer*
Where does that is it kind of upper thigh or is it kind of more like kind of on the outer labia or those kind of areas?

0:19:12.610 --> 0:19:59.890
*P3*
Yeah, more in the that that sort of area. So again, I, yeah, it's not like broken skin, it's like spotting. So, I can get maybe like one probably like one, like quite large spot which will go down after maybe 2-3 days and then it can be it. So then if I went on a ride again the next day, it can be a little bit uncomfortable, but I don't tend to, I have never had it so bad where it stopped me going out for a ride just yet. But I think I have found that actually it's worse depending on which seat, or which bike I have been on. I do use cream as well which has been a bit of trial and error to work out which cream seems to be most effective at stopping it.

0:20:12.340 --> 0:20:18.970
*Interviewer*
Yeah, so if that happens, do you just wait if you get like a small spot, do you just wait for it to clear up or do you use anything to treat it?

0:20:20.410 --> 0:20:35.900
*P3*
I usually just wait for it to clear up sometimes, put some cream on like some hydrocortisone or something like that. Just to try and stop it flaring up so much and reduce inflammation and moisturizer and so on.

0:20:39.990 --> 0:20:50.390
*Interviewer*
So you said in terms of likelihood of developing maybe a saddle sore, is it time on bike or is it saddle type or is there anything that seems to be related to it?

0:20:51.470 --> 0:21:14.980
*P3*
Yes, I think time on bikes, it’s after those long longer rides. So very rarely, kind of course I commute to work a couple of times a week, 2/3 times a week, and I've very rarely notice anything, so that's about an hour each way, so I've rarely kind of notice anything after a commuting day, so it is more, you know, after the long, long weekend rides.

0:21:16.340 --> 0:21:25.190
*Interviewer*

Does this ever affect, like your enjoyment of cycling? Or is it just like one of those cycling things to get used to?

0:21:25.570 --> 0:22:15.950
*P3*
It's one of those things you get used to. I’d rather, it wasn't there but I think actually, because as I said so that sort only tends to occur after a long bike ride, and then I probably wouldn't be doing that until a good few days later. Again, by which time would have calmed down, whereas in between it might be a commute to work, or just a short bike ride to get out. So, I'm just kind of like for the like the hour just sort of put up with it because it was. But yeah, if I was going out, if I got a saddle sore and then try to go out again for another 5-6 hour ride the next day then that might be a different story. That might not be so happy when I came home after that, but yeah.

0:22:18.380 --> 0:22:37.230
*Interviewer*
I think that's all my well, all my questions on that topic. I've just got one final question. Are there any areas you would like research in regarding female cycling so menstrual cycle, female specific training bike setup, saddle choices, injuries? Do you think there's anything we need to do any research into?

0:22:39.560 --> 0:24:10.10
*P3*
Yeah, I think it's more awareness around the menstrual cycle and how all your hormonal changes impact what you can do and how to like modify that. And I know that the research has been have been done in and you do, you do see research on it, but I guess at the minute it's still fairly limited as well. But I think it's not just the research, it's a raising awareness. I think even a lot of women don't understand that. So it's like how it's more about social aspect of how to raise that, that message so that people understand it and something that you touched on before about especially you know you kind of like think of like younger women and you, you know, girls coming into sports and I think they need to know from an early age of about what's healthy and what's not, and kind of those signs. And yeah, I would impact as well. Yes, I suppose thinking from that, if there was any research on impact, you know possible longer-term impact for young, younger athletes when they're training too much and you know if they train to a point where the impacting the menstrual cycle like any longer-term impact that they might see from that. Yeah, I have supposed just everything really on that side of things, yeah.

0:24:12.470 --> 0:24:16.50
*Interviewer*
OK, great. Have you got anything else you'd like to add before I stop recording?

0:24:17.220 --> 0:24:19.320
*P3*
No, I don't think so.

0:24:19.650 --> 0:24:19.870
*Interviewer*
OK.

**P4 – interview transcript**

0:0:0.0 --> 0:0:9.280
*Interviewer*
I will just start with a bit of background about your cycling and what cycle events do you currently compete in, and have you always been a cyclist, or have you done other sports?

0:0:10.740 --> 0:1:19.720
*P4*
So I've been a cyclist since I was about 8 when I joined a local club and I've not really done any other sports competitively. I've done a little bits of, I used to horse ride a bit, etc but I've never done anything in a committed way as I have for cycling. Probably started racing when I was about 14 and I did cyclocross and then I moved to do cyclocross, mountain bike and road and I've done all of them to a national level, up until I was probably about 20. And then that was COVID year. So that's 2020 and everything stopped, and I didn't compete for about two years. And then I've started getting back into cyclocross which I now compete regionally and nationally again. As of the end of last year, I started trying out some gravel events, so I'm kind of dipping my toe in the water with that and I've got quite a few planned this year, so mostly off road, cyclocross and gravel to a regional and national level……

0:2:39.280 --> 0:3:2.80
*Interviewer*
That's why I chose my current club because there's actually some women that race at club level without having to commit to lots of competitions. So, moving on to the menstrual cycle, thinking about when you start having your period, what was it like for you? How old were you? From what you have said, I presume you were cycling at the time. Did that have any impact?

0:3:3.520 --> 0:4:17.220
*P4*
Yeah, I can't. So, I can't remember specifically what age I was, but I know I was a little bit of a late starter. I think I was around 15 or so. I don't actually remember it initially affecting my cycling at all. I think, part of that is like I've always written with my dad and his friends, and you know, everyone's male, because that's what happens when you're in cycling. And I suppose there's a little bit of that not wanting it to affect you. So, you just kind of get on with it anyway. I was never embarrassed or ashamed or felt like I couldn't speak to someone, but it was just, it wasn't gonna stop me. It was, I think when I was about 16-17, I went on to a combined pill and then that stopped any symptoms that would affect my cycling, so, cramps or anything like that and it was only when I came off that a couple years later that my cramps started coming back and I was an adult and then it started to affect my cycling. So, at that point I, well, it's not too hard sessions on the first couple days of my menstrual cycle because I have really bad cramps.

0:4:18.930 --> 0:4:27.310
*Interviewer*
And when you first start getting period obviously said you, you didn't have a problem talking to anybody about it. Did you get any education around that age, like school or?

0:4:27.510 --> 0:5:11.290
*P4*
No. Uh, yeah, in in general, not sports specific. Yeah, we definitely had something in school. I suppose one of the biggest things for cycling is what products to use and using tampons. But again, I think that's quite lucky of having a mum that was just quite open to tell me and let me know how to do that. So, I didn't really run into that issue. Whereas I can imagine if that's something that intimidates you, as a cyclist, that is really what you need to use, for your bibs, your bib tights. So yeah, pretty good.

0:5:6.650 --> 0:5:14.790
*Interviewer*
You mentioned the obviously you went on the combined pill and then I think you said you went on like the mini pill or progestin only pill as well as that?

0:5:20.480 --> 0:5:20.720
*P4*
Yes.

0:5:16.330 --> 0:5:24.940
*Interviewer*
And then once you've come off that the cramps have come back, would you take pain medication for those every month?

0:5:24.240 --> 0:6:10.350
P4
Yeah, definitely. So, when I think it might be both times, I came off the pill. They were much, much worse than. I mean now they've completely settled them. I don't take anything and they're much better. And so yeah, definitely, ibuprofen but I now still every month, pretty much every month, I still take out ibuprofen for the first day, sometimes two days but that stops it along as I kind of catch it early and then I'm completely fine and I could still ride my bike. Whereas when I came off the pills for that initial, probably three or four months I wouldn't ride my bike that day because it it's, you know, I was in bed. It was quite a lot of pain.

0:6:11.890 --> 0:6:18.10
*Interviewer*
Do you have any other symptoms of who mentioned cramps? Do you suffer any kind of mood related changes or anything like that?

0:6:18.860 --> 0:7:1.700
*P4*
Yeah, I most probably do. It's something that I find quite difficult to notice myself and I do sometimes like, you know, if you just watching something on TV and you cry and then two days later you start your period and you. Oh yeah, that makes sense. So, I definitely do have it. I just probably don't recognize it very well but yeah, probably tend to get a little bit more emotional and a bit more irritable, and I am definitely irritable on the first couple days when I have cramps and I don't know if that's also just pain related and just going like, I don't want to, it's an overload. If someone's annoying you, you just quite snap quite quickly.

0:7:3.980 --> 0:7:16.820
*Interviewer*
I'm in terms of your symptoms obviously said the cramps have settled down, but does it make? Would you still go on a long ride on the first couple of days of period or would you not, for like maybe needing to have a toilet stop if you go for a long ride, or kind of anything like that?

0:7:17.920 --> 0:7:19.130
*P4*
Yeah, I'd probably. I'd definitely tend to do less, just in in general in my life, whatever I'm doing, I'm definitely doing less for those couple, first couple days and that does relate to cycling, yeah.

0:7:31.550 --> 0:7:40.420
*Interviewer*
You said obviously you're not, in terms of symptoms but do you do anything to track your menstrual cycle or is it just when it's due to start?

0:7:41.420 --> 0:7:58.0
*P4*
I don't, I don't use anything to track. I have used apps previously, but I don't currently use anything. I don't really feel like I need to because the pain has died down that I don't need to be too prepared but yeah, I know roughly when it is.

0:7:59.590 --> 0:8:5.150
*Interviewer*
Do you consider yourself to have heavy periods or is that something that's bothers you?

0:8:5.970 --> 0:8:20.490
*P4*
Heavy on usually day two and then the rest of the time I'm pretty, pretty normal. Nothing that causes difficulties, and needing extra provisions or anything.

0:8:22.170 --> 0:8:31.280
*Interviewer*
This is quite a specific question, but do you experience as any changes in core strength, coordination, mobility or flexibility throughout the menstrual cycle?

0:8:32.890 --> 0:8:36.460
*P4*
Gosh, that's it's not something I've ever noticed or thought about.

0:8:37.310 --> 0:8:44.690
*Interviewer*
OK. Again, you don't have to. It's just some people notice particularly around their period that they may have yeah reduction.

0:8:42.340 --> 0:8:45.100
*P4*
Yeah, I'll be thinking about it.

0:8:46.990 --> 0:9:2.520
*Interviewer*
In terms of impact on your training, you said you typically do less for the first couple of days of your period. Are there any other provisions you take for that? Do you have more recovery or modify sessions?

0:9:4.100 --> 0:9:39.480
*P4*
Definitely less hard, so I would usually use that day as a recovery session and just something really easy. Quite short as, as we say, not, not as long duration and but otherwise no. I know that sometimes around that, that kind of time in those first few days, even if I'm feeling OK, I have to knock off the resistance on Zwift. You know, turn down the bias or don't do quite as many reps, but yeah, I probably just take quite a few of those days a bit a bit easier.

0:9:41.320 --> 0:9:48.120
*Interviewer*
How do you manage it around competition? If the competition falls on, particularly on those first few days of your period?

0:9:48.980 --> 0:9:49.820
*P4*
I just get on with it. I think it is just that mentality of especially if you've paid for it. I have paid for it so I'm just gonna do it and you know, that probably doesn't help emotionally because I know that I won't do as well as I want to. And then you have to deal with that. But I would, yeah, I would just keep doing the event.

0:10:13.920 --> 0:10:16.490
*Interviewer*
So just take a couple of painkillers and yeah, get on with it.

0:10:16.130 --> 0:10:20.390
*P4*
Yeah, yeah, yeah. I would take ibuprofen if I needed it and then just get on with it. So yeah.

0:10:22.370 --> 0:10:36.990
*Interviewer*
I think you mentioned on one of the questionnaires that maybe when you were youth rider, you have maybe had a bit of education from BC on the menstrual cycle. If you had anything else, advice around periods / menstrual cycle as a female athlete?

0:10:37.720 --> 0:11:48.180
P4
Yes. So that was, so that was actually recent. So, well, it's it was a confusing way of wording it, so I've never had any education on it, at all, and I've gone through like I've been part of a go ride club. I've been part of teams. I've, you know, I've really been in the system and it's only a couple months ago, British Cycling were hosting something for youth riders and I was like can I just sit on it and find out? So, that's the only education I've had, and it was very useful because I had no understanding of how it impacts my training at all. I suppose, except from what you personally feel, but having that acknowledgement helps, and to know what to do during those times. I think I hadn't realized that there are actually periods throughout the month where you know you're stronger and you're better and it's you can do certain types of training and that's really helpful. I don't only ever had these negative associations, so that's really quite nice. But yeah, that's the only information I've ever received.

0:11:49.610 --> 0:11:54.480
*Interviewer*
Yeah, because have you been a coached rider in the past or have you always just self-coached?

0:11:55.90 --> 0:12:48.990
*P4*
So kind of, so I've never properly had a coach, but I've had a dad's friend who was a coach and he's giving me some sessions before. When I that was probably when I was maybe late teens like 19 or so, and I went back to him last winter because he was completing his Level 3 and he was using me as a guinea pig, which is fair enough, and that was the only time that I felt I suppose comfortable to mention it to him. And just that I was, I think it was just that, you know, I have cramps at this point. I'm not going to do something hard, but we didn't really discuss it beyond that and that's not his fault. It was just part of his own, you know, training. But I would feel, you know, happy to approach it with someone, but I am not the coach, so yeah.

0:12:49.720 --> 0:13:16.930
*Interviewer*
Yeah, because that was, yeah. One of my questions about how comfortable you'd feel having conversations kind of around this topic. And one other thing is, are you aware that in the media over maybe the last couple of years there's been more discussions, I suppose around female athletes and the effect of periods? So, Dina Asher-Smith spoke about it? Eilish McColgan as that. Have you noticed any of those stories or made you reflect on anything about your own experiences?

0:13:17.960 --> 0:13:43.610
*P4*
Yeah, I probably haven't noticed anything quite specific, but in general, yes, that there's, people are a lot more comfortable talking about it, which is very positive. I follow pages such as like Wellness HQ or Well HQ, whatever they are. So, I do see it more in my own eye line, which is fantastic, yeah.

0:13:44.580 --> 0:13:56.300
*Interviewer*
Yeah, because so far, my awareness is there hasn't been maybe any female cyclists who have spoken openly about it, it has been so for other sports and obviously women's footballers big one as well for discussing it.

0:13:56.820 --> 0:14:12.850
*P4*
Yeah, I can't think of anyone either. I'm because we have a lot of positivity in other areas, as in you know, we've got like Laura Kenny and Nikki Brammeier are being like female mums and things like that, but it's probably not specific with periods, yeah.

0:14:15.800 --> 0:14:22.40
*Interviewer*
OK. That's all my questions around the menstrual cycle. Do you have anything you'd like to add or feel I haven't asked you about?

0:14:22.900 --> 0:14:23.630
*P4*
No, don't think so.

0:14:25.880 --> 0:14:43.300
*Interviewer*
OK, so going on to the second part of the interview, from looking at your questionnaire, I think you don't write any women's specific bikes and you ride all well what we'd call male frames or unisex? Have you made any modifications to those since you've bought them to set them up for you specifically?

0:14:44.390 --> 0:15:26.330
*P4*
Yeah. So, I always put small bars on. I ride 38’s and often frames come with 40’s or 42’s, which mostly I just like for men and women if you're buying a frame size that is that small, I always just think that's so daft that the boss is so big. The only thing I do is replace the saddle. Again, it's not female specific saddle. It's just that suits my body. I think that's what you need, which is hence why I don't buy female specific bikes. It's just something that fits my size. But those are the two things. I don't change gears or anything else like that.

0:15:26.890 --> 0:15:29.870
*Interviewer*
You don't change crank length or things like that.

0:15:30.500 --> 0:15:34.740
*P4*
No. Again, frame size tends to come with what I want, so yeah.

0:15:35.580 --> 0:15:42.450
*Interviewer*
In terms of like your saddle you said that, what suits you, how did you arrive on that saddle? Was it trial and error? Recommendations?

0:15:43.100 --> 0:16:26.200
*P4*
Trial and error. Yeah, but getting a saddle that suits you is such an expensive, you know situations do. I think it's a good idea to go to an actual fitter and but yeah, I had a couple of specialized saddles with cut outs in the middle, so I knew what I liked out of the saddle and then the one that I've actually settled on is because I was in Spain with some friends, and I wasn't getting on with my saddle and I tried my friends, and it was really good, and I bought it off them so now I'm. I'm hooked and I buy them on eBay when they're cheap. So yeah, I think it's just trial and error, yeah.

0:16:27.750 --> 0:16:36.190
*Interviewer*
Do you suffer from any cycling overuse injuries? So lower back pain, knee pain, saddle sores, foot pain, hand or finger numbness?

0:16:37.600 --> 0:16:59.950
*P4*
I get saddle sores even though I really like my saddles. I don't, I don't really know why, but I often get saddle sores. I don't really get anything else at the moment to be fair, not riding so much to get overuse and it's more and more under use at the minute and but yeah, just saddle sores.

0:17:1.230 --> 0:17:11.700
*Interviewer*

So presumably still affect you on the cross and gravel bike. Is there anything that make it worse? So, time on saddle, like time of ride or anything like that?

0:17:12.840 --> 0:17:47.760
*P4*
Yeah, it's, so staying in my chammie. As soon as I get home, I'm like in the shower straight away. I don't really like stopping at cafes because I just don't like sitting there in a chammie. Definitely, probably terrain doesn't help, which is great for the sports that I do, and I know just that it is definitely worse if I'm doing like a rough gravel race and it's usually something that I just don't notice until I've finished. So yeah, probably terrain, duration and time in chammie, definitely.

0:17:48.600 --> 0:17:58.610
*Interviewer*
Where do you suffer saddle sores? Is it sit bones or is it more kind of upper thigh, vulva, inner/outer labia?

0:17:59.650 --> 0:18:5.410
*P4*
Yeah, it's upper thigh. Yeah, kind of outer labia area, yeah.

0:18:6.630 --> 0:18:14.880
*Interviewer*
Without being too graphic, is it kind of bruising, pressure, chafing or do you get kind of like little cysts or spots?

0:18:15.370 --> 0:18:23.230
*P4*
Little spots and cysts. Sometimes they're not really, actually cysts, as in there's nothing really there and, but yeah.

0:18:24.950 --> 0:18:34.850
*Interviewer*
And in terms of treatment how would you normally treat a saddles or would it just be time off bike or would you need to get any like kind of antibiotics or medical treatment?

0:18:35.410 --> 0:19:23.190
*P4*
So I have. I did go to the doctor's once because I had one that was repeatedly coming back but to be honest, they were not very useful. It's really, it's as you all know, it's difficult when you're an athlete going to the doctor's about something because the response is just, well, just don't use that or don't ride or don't do it, which at that period I think I'd had like a month off the bike and it was still there. I'm going well, I'm not gonna, like how long to not ride my bike. I actually most of the time don't treat them. They are not bad enough to cause me that many issues. If they are particularly bad, I would put like a like sudocrem or something on them, but other than that I just carry on.

0:19:24.660 --> 0:19:31.380
*Interviewer*
Does it ever affect your ability to train a compete? If you ever had to miss training and competitions because of them?

0:19:32.670 --> 0:19:45.670
*P4*
I've definitely missed like a couple turbo sessions because you sat still. It's quite hard, and but yeah other than that, not really. I just really just get on with it.

0:19:47.450 --> 0:19:54.270
*Interviewer*
Classic cycling mentality, does it influence your enjoyment of riding?

0:19:53.410 --> 0:19:56.800
*P4*
Oh yeah, it there it can do, especially on the turbo, if you are sat there, and the only thing you can think about is your saddle sore, that’s unpleasant.

0:20:3.190 --> 0:20:10.690
*Interviewer*
In terms of turbo position, are you do you long turbos or do you tend to keep them short because of the discomfort? You can get discomfort in the saddle.

0:20:11.360 --> 0:20:22.140
*P4*
I keep them short, but just for my boredom, because I really dislike the turbo. So, I can't bring myself to do more than an hour on the turbo. Yeah.

0:20:23.350 --> 0:20:47.780
*Interviewer*
No, that that's the same here. Saddle is discomfort and boredom. Have you. I think you've mentioned there about trying to prevent settle sores about being really particular, about not sitting in chammie and showering. Have you done any other changes apart from the saddle and kind of it? Have you ever sought any other treatment apart from the GP, if you change your bike setup in terms of saddle tilt or anything like that.

0:20:48.460 --> 0:21:23.300
*P4*
Umm, no, I've tried. I've tried different shorts. I had. I was probably riding old team kit. That was I had realized how long since I've been in that team and so then bought myself some other shorts. I'm not sure it really made a difference. I haven't changed bike position. I do check my position if sometimes because I've got like cyclocross. You need two cross bikes so want them to be in a very similar position, but I haven't tried any other changes.

0:21:25.550 --> 0:21:30.760
*Interviewer*
That's the end of the questions on bike set up and saddle sores. Have you got anything else you'd like to add?

0:21:31.690 --> 0:21:32.290
*P4*
Don't think so.

0:21:33.720 --> 0:21:44.10
*Interviewer*
And final question, are there any areas you would like researching, in regard to female cycling so menstrual cycle, female specific training, bicycle setup, saddle choice, injuries?

0:21:45.790 --> 0:22:38.930
P4
Ooh, that's very good question. I think I think the things around saddle sores are really good. Speaking to friends that is something that is really common in women's cycling. I've a friend who's a pro cyclist and she sometimes has to have, you know, periods of time off because of saddle sores. And she's gone to XYZ doctors and everything. And this is her job. And you know it. It stops her from being able to compete, so I think it's, it is definitely something that would be fantastic to have more knowledge in. I have a lot of male cycling friends. None of them really seem to complain about saddle sores or very, very rarely, and so yeah, that's definitely a good area. But yeah, I just very interested to see what comes about really.

0:22:41.20 --> 0:22:45.660
Interviewer
OK, that's great. That's all my questions. So, I'll just stop the recording.

**P5 – interview transcript**

*Interviewer* 0:04

So, we'll just start with a bit of background. Have you always been a cyclist, or have you done other sports?

*P5 0:15*

I mean from school, from being a kid, if there was anything sporty to be had at school from Juniors onwards, it was like quick cricket, yes, touch rugby, yes, netball, yes, rounders, ok. Like, you know, that was me growing up. High school was your typical netball, cross country. You know, just whatever they get you to do in PE. But I did do like extra netball outside of school as well. And then after that. I always rode like me bike as a kid for fun. Like, I lived on me bike pretty much, so grocery shopping - big pints of milk hanging off each thing trying not to hit the tyre with the carrier bag and go over the top, so all of that kind of stuff. But as far as competitively that didn't start really until I was about. Oh, how old would I have been in a in 2011, if I am from 41 now, but yeah, so about 2011 I did, I sort of jokingly say to a friend about doing the Great North Run. So, we decided that we would do that and that was kind of the start of the running. But you know, between sort of high school and 2011, it was just sort of gyming, going to the gym and stuff. I remember not, like, I remember being like running for the first time I ran for 20 minutes on a treadmill and being like, yes, and then, and then after that it was just like, it just progressed and progressed until I got injuries, main injury kicked off around about 2015. That stopped us from running properly, and then I kinda bought a bike which was the wrong size for us, first ever sort of drop handle bike. I remember riding it, absolutely crapping my pants because I'd always had stuff with suspension or mountain bike kind of setups. So it was so rigid, and so like foreign feeling, and but didn't get massively into it straight away cause I found it too uncomfortable, downstairs. So, from that I then, instead of having like a second hand bike, I went to a shop and got like a proper bike. But well, not the second hand bikes aren't, but the one that was more geared up for my size. Still didn't really find a massive love for it straight away. Again, just discomfort in saddles over long distance. But I was going out, and doing, like, I was one of those kind of people who was just doing big miles on weekend but never really riding any other time. I've used me bikes and things that I've had just for commuting purposes when I've, but that wasn't until I got the Grey Cannondale, which is kind of like my commuter bike. And I would say, the only events that I had done really were like the Wooler Wheel Challenge, and I did that a couple of years running and then it was mainly. I would say when I met my partner that I started competing in the cyclocross and then from the cyclocross it went to the TT's. And, you know, bits of triathlons here and there, but yeah.

*Interviewer 5:46*

Yeah, that's great. OK. So, we'll move on to talking about the menstrual cycle, so thinking about when you started your period, what was it like for you? How old were you? Did it affect playing any sport at the time?

*P5 5:58*

I was eleven. It was in the summer holidays. I was at me Nana's house. Me Nana obviously didn't have anything at all because she had passed that stage of life. So, it was sunny, and I remember her folding up little pieces of cloth to put into my pants. And then ringing up me mam, going I am a woman. You know. As far, as far as sport goes, I can't say when I was younger that it ever really affected anything apart from like feeling a bit sort of squishy pants, and embarrassed, do you know what I mean, or worried, like, have I leaked, is something showing, you know, but I have always had very short periods and not an awful lot of issue. That didn't tend to come until I got a little bit older, so, and then it was just kind of like you know crampy diarrhoea, you know, just the norm. Competition wise, I feel like I actually race quite well on it. But, I don't know, I mean, as I've got to that sort of 38/39/40 mark and onwards that's I think it it's probably affecting us more but I think that's more do with maybes me diet and me age.

*Interviewer 7:48*

Yeah. Obviously looking back to when you first started, did you get any education or advice around periods or menstrual cycle?

*P5 7:56*

Yeah, we had the normal school thing, you know, and I remember, I don't remember having anything in juniors, but I remember having the talk in seniors..……. Right. And I what we're saying and.

*Interviewer 8:33*

If you have any educational advice on menstrual cycle / periods?

*P5 8:38*

Yeah, I don't. I don't remember. I mean, I was aware of stuff. It was never. It was never a shock. You know, what I mean? I knew it was coming. Females in the family must have spoke to us about it. Girls at school had already had theirs, I remember. I was in high school at the time, it was, just was it the summer holidays before though, I can't remember if it was the summer holidays before starting seniors, or actually the summer holiday during me first year of seniors, but yeah.

*Interviewer 9:08*

OK. You mentioned as you've got older, your kind of symptoms have changed, they were quite kind of short periods before, didn't really have anything, has, as I said have these like change in symptoms as you've got around 40, if they had any effect on you as an athlete in terms of racing or training?

*P5 9:37*

Maybe just feeling a bit flat, but then it's like but then we results have been all right. So, it kinda it's one of those things where I think if I had felt flat and then hadn't done as well then I would be thinking like, uh yeah me period would then become more of a factor in my brain. But if you feel flat but still podiuming or anything like, or have a pretty good time, or whatever. Then you just, you don't really make the connection that strongly, do you?

*Interviewer 10:21*

Yeah. In terms of symptoms you get, are they all during your period or do you get some like pre-menstrual, so the few days before your period, do you get symptoms as well or?

*P5 10:32*

Mainly the one thing that I've always noticed is, and past partners have noticed as well as about fortnight before I have one day of been an absolute bitch. That's what, like, the, that's the male phrasing of it. One of my partners in particular, but other ones have just kinda said like you, you'll have like about, you know, a fortnight or so before you get quite emotional. And that's when I'll have, like conversations with a partner about stuff that's been bugging us. Obviously it's probably the worst time because my emotions are all over the place and everything seems massive, and you know, so.

*Interviewer 11:07*

Yeah, and during I kinda suppose that menstrual cycle or during the periods you even it was any kind of changes in core strength or kind of coordination or flexibility to go with that or is that not a problem that bothers you?

*P5 11:31*

I mean you say that actually, because I was at yoga yesterday and could I hell balance or bend, and I came on my period a couple of days ago. And but I always I do feel like we coordination is not quite as good. Do you know when? I'll. I'll I was told you once about since COVID. I feel like me coordination is being a bit off and was off for quite a while after that. But yeah, like me, balance and stuff felt really dodgy after COVID, so I don't know if it's just a knock on of that still.

*Interviewer 12:20*

Yes, it was hard to ascertain what is causing it. In terms, I think you said, I'm like, yeah, you track your menstrual cycle on Garmin. Do you do anything with that information or is it just more it records it, and it records your cycle length.

*P5 12:36*

Yeah. Just to keep an eye on it. Because I never actually used to when I was younger, I never used to really know when it was gonna come. Not because it wasn't on time or anything like that. Just because I have a very poor concept of timelines, and yeah, I don't know. So it would just be sometimes like, you know when you feel the little, the little poop, like you can almost feel your ovary just go poop and like the little pinprick, of it releasing and it would be like, alright, I guess all that some pads, you know, so whereas now I'll just more so because when I got older and then I had my son. Like I had my son when I was 19 and when I was getting questions off like the nurses and stuff like that didn't know the answer, and I didn't, I didn't. It was. It was bad enough in some ways how they treated you because you were a young parent. But then, when you irresponsibly couldn't even answer a question about when your period normally is and how long it is and all the rest of it, it was like, it just it made us feel like I need to do better. So, I started kind of got the tracker watch when he was about. I mean, it didn't bother. It wasn't a bother, just that much at the time, because I didn't really start running. As I say, until 2011 and he was born in 2002, so I had no means of like tracking it until that, until 2011.

*Interviewer 14:23*

Do you share that information with your coach or anybody else?

*P5 14:28*

Sometimes, like, if he asks what happened, what happened there, like, I don't know. I came on, might be something to do with that.

*Interviewer 14:35*

It's in terms of like training and performance. Is that what you mean in terms of like training was down or something?

*P5 14:42*

I presume so. It must be cause that's pretty much the only time you'll ever sort of say what happened there, it'll be if you haven't done very good at something that he was expecting you to.

*Interviewer 15:00*

And do you feel comfortable talking about it with your coach?

*P5 15:04*

Why, Louise, you know me, Christ. I would tell the Pope I was on my period if it came up in conversation. You know, what I mean? Sorry if I was there and farted I'd be like, yeah, sorry, that was me. Pardon. I've got absolutely no shame. Put it this way, when I was in the bike fit and I was talking to my coach and were on about discomfort, I basically turned round and said to him that the main part of the pain was on me happy button. At which point I heard my coaches wife in the background like pissing herself laughing because she probably would have known how uncomfortable that maybes made my coach. But I didn't know how else to sort of say with main pain was, so I thought that was the most polite way to say it.

*Interviewer 15:56*

Have you ever had any education around like menstrual cycle and sport and phases and training or anything like that?

*P5 16:12*

No, not that I can think of offhand.

*Interviewer 16:15*

Are you aware of there, I suppose it's been more in the media in recent years. If you've seen any of the stories, maybe around Dina Asher-Smith getting cramps, so you haven't, because obviously footballers and ACL.

*P5 16:27*

No.

*Interviewer 16:28*

OK. It's just interesting to know whether that's made any impact.

*P5 16:31*

I don't claim honestly, very rarely watch the news and if I do catch anything, it's just by accident. So.

*Interviewer 16:37*

That's all my questions on the menstrual cycle. Do you have anything else you think you'd like to add, or you feel I've missed that I should have asked you about?

*P5 16:52*

No.

*Interviewer 16:54*

OK, moving on to your bike and I think in your form you've got you said you got three bikes: Scott Cannondale and Vytus. Any of these women's specific or are they just kind of I suppose unisex?

*P5 17:09*

I wouldn't know to be honest, if they were women’s specific or not. I mean, the commuting, Cannondale, that one, I went into a bike shop, they said how tall are you? They looked at us and they said this will be about you, and I tried a couple of bikes on their car park, and they were like that one is the better position, which is the one that I've got and. I couldn't tell you if that was a male or a female bike at all. Every other bike, like the cross bike. My partner got us that for like me birthday and it's the same as the one that he has. So, I presume it must be a boy's frame. The road bike is his old road bike, so that'll be boys frame, I would guess. The TT bike I bought that off a fella, so I presume that's a boy's bike as well.

*Interviewer 17:53*

Have you made any modifications to the bikes since you bought them? Like, have you changed handlebars or cranks.

*P5 18:18*

The only one that I would know that about would be the TT bike because. All the other bikes, my partner just tinkers as on with them and he has changed, like, he tells us. But, you know, it'd be like me telling him about makeup tips it just it doesn't sink in. It doesn't stick in my head, so it's like, what was it? I know he’d mentioned something about stacks on one of the cross bikes. So, what the handlebars go on to?

*Interviewer 18:51*

How high they are. Yeah.

*P5 18:54*

I think he had done something with them once. He's, like, we've had various bike fits with my coach and things, so things have got changed around, seat height and whatever else. Cranks I think we have always rode the same cranks. I don't know, whether. I mean, technically, most of the bikes than I rather his, so I don't really have much of a say over them and I haven't. I haven't done as any harm and I've got no, like discomfort, no injuries, like. Well, I say no injuries, but no sort of positional related injuries or anything that's gone back to a position. But TT bike, it is pretty much, apart from putting an extra thing in the where the saddle sits in, that the saddle attaches to, that's got like a little block in it to adjust it. Nothing. Nothing abnormal, just the normal bike fit stuff.

*Interviewer*

And based on that you haven’t had any problems with lower back pain, or knee pain, foot pain?

*P5*

Well, I've got a bunion. So sometimes on long rides, like, I'll get a very painful pins and needle foot which is quite uncomfortable. I mean, I think in a TT position you'd have to be some kind of weirdo not to get a bad back. Doesn't matter how good your position is like, that position, it just leads to that, you feel alright that minute. You'll know yourself when you're finished, and then you come back up for the first time its just like ohh, you know, so, but no, nothing more than the norm.

*Interviewer 20:58*

In terms of saddles, I think from your questionnaire you said use different saddles on different bikes. You have a different saddle for your TT bike. How have you kind of arrived on that saddle?

*P5 21:07*

It's been the best saddle that I've had so far. So, best saddle that I've had so far, I don't have any internal labia swelling, grazing or any damage like that, but I do get like a bit of sort of external pressure point bruising or swelling if I'm doing a particularly long session and so the day after getting back on the bike again and like slowly getting back into that position, waiting for the lips to go numb, you know.

*Interviewer 21:56*

So, is it more kinda of inner/outer labia or upper thigh that you get your problems?

*P5 22:03*

I used to get it on. I've got like one fat labia internal, like, one extra one that isn't on the other side. And it was on that extra one that I used to get the problem. Never really got much of a problem on the cross bikes, but again different position, but it's only one I've been road biking and TTing. So, on the. Is it the one that looks like satellite, but it's not satellite. Italia something or other?

*Interviewer 22:40*

Alright. Yeah, the Selle Italia.

*P5 22:42*

Yeah, those ones used to give is on the road bike and on the TT bike the internal swollen blistery kind of fat labia internally. The ISM split saddle that didn't get us internally, but I ended up with a really bad blood blister on the external of me groin. So, like, yeah, it just it looked almost kind of like when you get an ingrown hair, but there was no ingrown hair there and it just got big and bloodier and bigger and bigger and then it eventually burst. So, I moved away from that saddle. And then the one that I'm on now, as I say, it doesn't break the skin. It doesn't damage anything internally, it just, I just get like, a bit of tenderness in the pressure points. And obviously, if I've been on the bike for a long time, especially on the turbo, and I haven't moved around much, then I get like a bit of, I would, I would say it's almost like a hardish lump that's a bit tender, just like a normal swelling, but nothing, it's nothing like what I've experienced previously. It's like, it's manageable., it's like the best saddle I have had.

*Interviewer 24:08*

And how long do you need to ride for, for that to become an issue?

*P5 24:14*

Hmm, it's generally anything over the hour, so like, but only indoors.

*Interviewer 24:21*

So is it just TT bike or is it road as well?

*Interviewer 24:26*

Just TT bike.

*P5 24:28*

Yeah, I don't have that saddle on the road bike.

*Interviewer 24:31*

Yeah. Do you have a problem on the drops on the road bike? If you're on the drops for a prolonged period or.

*P5 24:39*

For me back or anything, you mean?

*Interviewer 24:40*

No, for saddle sores on the road bike. If you go on the drops, do you have problems?

*P5 24:48*

I mean historically maybe. Last time I was on the road bike would have been. Ohh now then. Maybe it's about three weeks ago. We rode round one of the TT courses just to check for potholes for the for the CTT people. I was on the Italia one, the Italia saddle and there was times was given us a bit of grief. But it's just because I'm not used to that saddle anymore. So it's, but I would say if anything, I think the drops at that point were more comfortable because it sort of puts the pressure where the TT saddles currently putting the pressure. Rather than when I'm upright, it ends up getting in the all the delicate internal areas, and the pad, it's pads more often than not I think more, then some of the saddles.

*Interviewer 25:51*

So, have you changed your shorts?

*P5 26:07*

Yeah, I opt for like a boys pad now because the ladies pads there too, there too padded and it just works its way into places I don't want it to be and causes friction and it, yeah, it ends up really sore even with like the even with the balm on the chammie, it's still, so.

*Interviewer 26:28*

If you get a saddle sore what do you do to treat it? Is it time off the bike? Do you use kind of repair cream or antibiotics, if you ever had anything that got infected or was it just rest.

*P5 26:51*

No rest, just, that femfresh that I told you about with the camomile in. That's magic. Kind of just soothes most of the sort of sore areas straight away and then just, obviously getting a little bit older you have to the issue with dryness, so making sure that you kind of don't have the dryness. And so, you know, just trying to use something that helps keep it to the moisture that it should be, helps as well.

*Interviewer 27:39*

Yeah, in, in terms of obviously, it sounds like your saddle sores are much better than they used to be, does it still affect the enjoyment of riding a bike, or is it?

*P5 27:54*

It did when we went round on the road bike like, cause I did have to stop and sort of dislodge me pad and get it in the back into an area where it's like was more comfortable, but then also then we were kind of we were trying to do a bit of a tempo things or maybe I was, if I'm totally honest, maybe using it a little bit as an excuse to get a rest. Because my partner is always so much stronger than I am. Like, I get sick of looking at the back of him.

*Interviewer 28:32*

I think you mentioned it that you've talked about your saddle issues with your coach in terms of bike fit up, has he helped you kind of get to the saddle, helped you choose the saddle you're at now.

*P5 28:45*

Yeah, it was his suggestion.

*Interviewer 28:49*

And so, in terms of like doing anything to prevent it, is it mainly just you've changed your saddle, make sure you wear shorts with a different - well men's chammie. Have you've done anything else to try and help and prevent saddle sores?

*P5 29:04*

Just that. Just the balm as well. But the one that I've got nearly ran out. And yeah, the place that my partner got it from doesn't have it anymore. So, I need there either try and find somewhere that sells that one, because I'm obviously reluctant to use different one in case it's not as comfortable or as good or if I have savage dislike for it like biologically.

*Interviewer 29:18*

That's all my questions kind of on bike set up and saddle sores. Have you got anything else you'd like to add?

*P5 29:42*

Umm, no.

*Interviewer 29:46*

And just one final question, are there any areas you would like researching, in regard to female cycling, so menstrual cycle, specific training for females, bike set up, injuries, saddle choice?

*P5 30:02*

I don't think. I mean, I have spoke to you about this before. I do think it would be handy for women and companies that sell saddles to give you, some kind of, like, you know, we'll buy clothing and it's got size and on it, we buy skin suits and all the rest of it, and you have to give various different like measurements for all this different area. I don't see why something that's so prolific as a saddle that causes damage to a woman and can affect our performance why there's not more information available about, you know, if you this kind of shape, this is probably the better saddle for you. This is a better pad for your shape because I don't, I really think it's very short sighted for and I know it's for manufacturing purposes and cheapness for them. But really, if there was a company that was making shapes specific pad or a shape specific saddle and charging more money I would buy it. Because who wouldn't? For the shear comfort. So definitely something where, where it's kind of like, right? What saddle works for you? This saddle works for me in this position on this bike. It works this saddle in this position. OK. What? What she kind of the shape of you as a female downstairs. And I know it's quite embarrassing, but there is people out there who are prepared to go all out, right? There's me there. I mean, look at that. Flipping that show, the one where on the TV looking at each other naked where, I mean, if there's people who are prepared to do it for that purpose, then I think there's just as many sports people that would be prepared to do it for a beneficial purpose of helping. So, I mean, I would be a bit shy about it, but if it helped other people, I’d probably going OK, you can have a picture of me vag. You know, what I mean? And these are the saddles that suit it, and these are the pads that suit it. Thought, there you go.

*Interviewer 32:32*

OK, that's great. If you got anything else, you'd like to add before I stop the recording.

*P5 32:38*

No.

**P6 – interview transcript**

0:0:2.350 --> 0:0:9.240
*Interviewer*
Just to start with a bit of background about you and cycling. Have you always been a cyclist, or have you come from another sport?

0:0:10.150 --> 0:0:29.240
*P6*
Well, done loads of sports growing up. Always cycled but not more than just kind of going out for the occasional ride and then probably about 10 years ago got a road bike and started to train properly. So proper cyclist for about 10 years.

0:0:30.490 --> 0:0:34.380
*Interviewer*
How is training and competition going at the minute? Have you started racing this season?

0:0:34.990 --> 0:1:1.280
*P6*
Yeah, I've had one race this year, got ill, missed the 2nd, and the third one was cancelled, say just kind of started. I say training is going pretty well but you just get such, I think it's that time of year where you get such peaks and such troughs and you don't quite know how you're going to have some really, really good days and then other ones you just end up crawling.

0:1:2.860 --> 0:1:5.40
*Interviewer*
Have you got any targets for racing this year?

0:1:6.180 --> 0:1:11.880
*P6*
Yes, so National Masters Road race, it's probably the number one target really.

0:1:14.60 --> 0:1:18.510
*Interviewer*
I think it’s on your questionnaire that you are mainly road and crit racing?

0:1:19.100 --> 0:1:20.240
*P6*
Yeah, yeah.

0:1:19.970 --> 0:1:31.920
*Interviewer*
OK, great. So, I'll go on to the menstrual cycle. So, thinking back to when you first started your periods, what was it like for you? How old were you? Were you playing sport then?

0:1:32.730 --> 0:1:59.500
*P6*
Uh, yeah, I think it was 13, played hockey, tennis, kind of like school sports I suppose. You know, what it was just inconvenient, still is, you just get that bit of the month where you feel a bit feel a bit rubbish, but it's I suppose past that didn't really bother me. It's just inconvenient.

0:2:0.350 --> 0:2:2.540
*Interviewer*
Yes, it didn't stop you playing any of those sports then?

0:2:3.140 --> 0:2:6.90
*P6*
Nope. Probably got worse with age, actually.

0:2:8.690 --> 0:2:15.450
*Interviewer*
When it first started getting periods was, did you get any education or advice around periods or menstrual cycle from anybody?

0:2:16.850 --> 0:2:33.130
*P6*
Yeah. So, like my mum, little bit, and then, you know what school, a little bit, but they kind of shied away from it a little bit. I think they're a bit rubbish on that side of things, but I suppose enough to know what it was and what to do.

0:2:35.740 --> 0:2:41.650
*Interviewer*
so you mentioned there that it's changed, maybe got worse as you've got older. How has your periods or menstrual cycle changed?

0:2:42.190 --> 0:3:10.930
*P6*
Yeah, I think, you know what, I think, I think maybe I'm just more self-aware. So, it kind of recognize like, this is probably why I'm feeling grumpy, or this is why I'm not sleeping and maybe they're more, I had a period of time where they weren't regular, so maybe now they are more regular. I kind of, yeah, I'm just a bit more in tune with what's going on. I say my 20s, didn't really, didn't notice, didn't really affect me at all.

0:3:12.560 --> 0:3:19.760
*Interviewer*
Yeah, you mentioned there that they had a period where there were less regular. Was this like related to health condition or was it related to training? Or do you not know?

0:3:19.620 --> 0:4:15.70
*P6*
I think, I think under fuelling and training, and so I'd maybe about coming two years they came back, got some support to get them back, the NHS were pretty useless. So went to see somebody else privately, and then a couple of years ago did the same, which must have gone for about, say, maybe six months. I think when I get stressed, I just tend to under fuel, but I'd say I'm working with a nutritionist now and I'm just so much more aware of, like, what you need to get your body to do what we do as cyclists, which is quite astounding when you look at how much you end up eating.

0:4:3.260 --> 0:4:29.690
*Interviewer*
Yeah, but it is/can be a common problem in endurance based sports, the kind of under fuelling because, yeah, it might be normal for a person just having normal lifestyle, but it's not enough for the level of training that goes with it. So, in terms of probably talking about how you are now, I think you're back being regular. Do they have any impact in terms of training? You've mentioned maybe mood or feeling a bit sluggish.

0:4:30.640 --> 0:5:9.980
*P6*
Yeah, yeah, I probably get, definitely get like the week before I don't sleep well - that's probably the worst bit. I just train through it and just kind of appreciate that sometimes I'm not gonna be as good. I've had a couple of races that have fallen on like day one and I get it this, it’s really weird, but I feel like I'm asthmatic on that day literally can't breathe and that that's the most annoying thing in terms of cycling the like the performance impact.

0:5:4.190 --> 0:5:19.790
*Interviewer*
Yeah, and you can't schedule races around your periods unfortunately. So, for you is it that kind of premenstrual where you don't sleep particularly well and then that probably like the day one, which is symptoms wise the?

0:5:18.470 --> 0:5:22.400
*P6*
Yeah, yeah. After that I feel fine.

0:5:23.190 --> 0:5:29.210
*Interviewer*
Do you ever have to? Do you ever take any pain medication for cramps or lower back pain or anything like that?

0:5:29.910 --> 0:5:43.130
*P6*
Yeah, probably the first two or three days and then I can pretty much just it, ignore it and that just seems to get me through it quite, quite happily.

0:5:44.400 --> 0:5:55.0
*Interviewer*
In terms of symptoms, do you ever suffer any that kind of effect you sitting on the bike because obviously it can be quite aggressive aero position when you're either on the drops or quite low does that ever bother you on your period or?

0:5:56.50 --> 0:6:15.490
*P6*
Sometimes I get lower back pain. So, I'd say that's more or like muscle, like weird muscle aches. So probably, yeah, I'd say the lower back and I sometimes just feel sick, like nausea, which kind of when you cramped over and you try to stuff your face isn’t particularly nice.

0:6:18.440 --> 0:6:26.530
*Interviewer*
Do you get any kind of mood related symptoms or changes in mood around phases in the cycle or is that not something that affects you?

0:6:27.260 --> 0:6:48.890
*P6*
Yes definitely before, like, the five days before, just irritable. Then maybe say, I don't know the first, not when I'm on my period, but after that maybe for like the next 10 days I am quite, quite happy, quite motivated, like feel more motivated than I would other times.

0:6:51.260 --> 0:7:0.110
*Interviewer*
In terms of adapting to symptoms, you said you often suffer like disturbed sleep. Do you do anything like try to sleep for longer or any kind of strategies to kind of cope with any of those symptoms?

0:7:0.810 --> 0:7:20.120
*P6*
Yes, I've got. I've been taking some, it's called Calmeze MegaMag, like a magnesium supplement for trying to do it the eight days before to see if that helps. And just going to bed earlier. Like try to get upstairs by like 9:00 o'clock. So, I've got more chance of sleeping for longer.

0:7:20.640 --> 0:7:28.600
*Interviewer*
Would you say you have heavy periods or does that affect kind of training in terms of long rides or going out on the road?

0:7:30.120 --> 0:7:38.630
*P6*
No, actually maybe one in maybe couple of years are a bit heavier, but not. I wouldn't say it affects it.

0:7:39.10 --> 0:7:44.520
*Interviewer*
So it isn't a concern that you need to have a toilet stop or anything on a long ride during period.

0:7:45.340 --> 0:7:45.520
*P6*
No.

0:7:46.330 --> 0:8:1.770
*Interviewer*
This quite a specific question. Do you have any changes in kind of core strength, coordination, mobility, flexibility throughout the menstrual cycle? Are there any time you find that like maybe core work worse or gym work or anything like that?

0:8:2.240 --> 0:8:28.190
*P6*
Balance is completely off, I suppose around being on, just all over the place. Then probably actually, probably, say maybe when I'm on, kind of that initial bit, I probably feel quite good in the gym quite strong which seemed counter you're feeling rubbish but actually I'm actually lifting quite well. Yeah definitely a coordination.

0:8:29.710 --> 0:8:38.700
*Interviewer*
In terms of obviously a lot of its your own perception, do you ever track your power data or anything? When is it like turbo sessions that you know it's maybe a down and a bit? Or is it more just how you perceive you feel?

0:8:39.680 --> 0:8:59.140
*P6*
Uh, yeah, I think, you know what I'd say? Probably the numbers are probably similar. It's probably the perception that then makes the numbers lower rather than you can't put out the power. I think, yeah, it feels harder.

0:9:1.550 --> 0:9:6.170
*Interviewer*
I think, do you use a smartphone app to track your menstrual cycle?

0:9:6.580 --> 0:9:6.960
*P6*
Yes.

0:9:7.710 --> 0:9:13.370
*Interviewer*
Is it just when your periods coming, or do you track anything else in terms of like how you feel to go alongside that?

0:9:14.220 --> 0:9:24.70
I tried like, if I'm not sleeping that well or just like weird, weird symptoms that I got a bit odd. So, then I just track it just in case.

0:9:25.530 --> 0:9:30.160
*Interviewer*
And do you share this with your coach and share any of this with your coach?

0:9:30.760 --> 0:9:42.420
*P6*
Yes, I think most of it actually just downloads onto training peaks. So, it's just it's just there. So, he's got a good idea of, he's got a good idea of how I'm feeling.

0:9:44.30 --> 0:9:46.360
*Interviewer*
Do you ever discuss it with him specifically?

0:9:47.350 --> 0:9:57.930
*P6*
Yeah. Actually, his wife always had really bad periods and is going through the menopause. So actually, we chat about it quite a lot. So, he's quite, he's quite good.

0:9:58.530 --> 0:10:15.330
*Interviewer*
Yeah, because that can be one of the challenges of a male coach, you know. I think you had said you had some advice. I think from your nutritionist around menstrual cycle? Have you had any other advice, or was it just being guided, particularly round suffering RED-S and kind of fuelling?

0:10:14.830 --> 0:10:15.670
*P6*
Yes, mainly around that.

0:10:18.270 --> 0:10:28.30
*Interviewer*
In terms of having conversations around the menstrual cycle. Obviously said quite lucky, you can talk to your coach. Do you talk to your teammates or other/anybody else about it?

0:10:28.560 --> 0:10:56.170
*P6*
Yes, so actually, for the team, we've done some like zoom webinars on different topics actually things like hormones, menstrual cycle. I coach a rider with my coach and she's quite aware of it, so we talk about that as well. Then my wife, we obviously talk about it. So yeah I probably talk to quite a few people about it.

0:10:58.910 --> 0:11:11.190
*Interviewer*
You have said, sometimes falls on a competition, maybe day one? Is there anything else you do differently if you know your periods gonna fall on the day of a race? Anything to do with your prep or fuelling?

0:11:11.960 --> 0:11:26.370
*P6*
Yeah, I probably, definitely, the couple of days before I come on, I always, I always feel a bit hungrier, so I'll probably just add a couple of extra snacks just to keep on top of it, that is probably the main one.

0:11:28.260 --> 0:11:41.940
*Interviewer*
Are you aware there's been quite a bit maybe in the media over the last couple of years, about elite athletes talking a bit more about periods and menstrual cycle, likes of Dina Asher-Smith or Eilish McColgan. Are you aware of some of the conversations going on?

0:11:44.570 --> 0:11:51.690
*P6*
Yeah, I think so. I've read different bits and just seeing female athletes talk about it. It's quite nice.

0:11:51.510 --> 0:12:2.540
*Interviewer*
Would you say that's had any influence on you like seeking maybe more information around the menstrual cycle or getting advice on it?

0:12:3.470 --> 0:12:20.210
*P6*
Yeah, maybe the, just the understanding of the different phases and I know everybody feels different, but just trying to get. Yeah, I suppose trying to tie how I feel to how my, like, what my body's doing at that time to makes sense of it is quite handy.

0:12:20.930 --> 0:12:29.500
*Interviewer*
Yeah, and that's all my questions related to the menstrual cycle. Do you have anything else you think I've missed, or you'd like to add or would like to talk about?

0:12:33.200 --> 0:12:35.310
*P6*
No, it's probably good, yeah.

0:12:36.300 --> 0:12:45.950
*Interviewer*
So moving on to the kind of the more bike, and the kind of injuries related to that. And I think you ride a very small bike?

0:12:46.460 --> 0:12:46.680
*P6*
Yes.

0:12:45.960 --> 0:12:47.810
*Interviewer*
Have you got 650 wheels?

0:12:47.480 --> 0:12:48.130
*P6*
Yeah, it's tiny.

0:12:50.50 --> 0:12:54.880
*Interviewer*
And so are both of your bikes women specific? If you ride small.

0:12:54.310 --> 0:12:57.590
*P6*
Yeah, they all are actually.

0:12:59.730 --> 0:13:7.250
*Interviewer*
Obviously they are women's specific, but have you made any further modifications to suit you as a rider? Crank length, handlebars, stems that kind of thing?

0:13:6.710 --> 0:13:43.10
*P6*
Yes. So actually, they both came with, road bikes got 165 cranks, already came with diddy ones. I think my gravel bike is 170 but I don't think they actually make GRX in a 165, so that could be why. They've all got the same saddle. So, swap the saddles. The handlebars are quite narrow anyway. Then pedals so put Speedplays on for a bit more float for previous knee injuries which seems to, seems to help.

0:13:43.810 --> 0:13:51.650
*Interviewer*
Yeah, that was one question I was gonna ask. Have you ever suffered from any overuse injuries in cycling, like lower back pain, knee pain, foot, hand pain or saddle sores?

0:13:52.590 --> 0:14:39.990
*P6*
Yeah, knees. I've dislocated right knee, had surgery years ago and then dislocated the same knee, so I'm always aware of knees but actually probably strength work is kind of got them as like as good as it's gonna be, so they don't bother me. And then really weird ones, say, occasionally in races at the end. I don't know if it's cramp. I don't know what it is, but I get the most debilitating leg pain and it's happened when going for a walk before. I don't know if it's tied to lower back pain, or, I don't know what it is. I'm I've been looking at bike fit recently to see if there's something that's triggering it, so it's a weird injury that I can't explain.

0:14:41.290 --> 0:14:44.740
*Interviewer*
Have you had a bike fit in the past or was this being new thing to will get a bike fit?

0:14:45.320 --> 0:15:12.250
*P6*
Yeah. I have both of them fitted and then refitted about a year ago, but I because of the cramp I went back to see a different bike fitter who's lowered my saddle a little bit. So, I'm now trying that and then trying to find, I have quite wide feet to try to find a pair of cycle shoes that don't mush my toes.

0:15:11.990 --> 0:15:15.780
*Interviewer*
Is it like you toes or is it like your metatarsal joints that you get the pain in?

0:15:16.420 --> 0:15:23.350
*P6*
Is that like the? Is that like the end of your little, you know, your little toes are like, I've got, like, little weird, little curly little toes.

0:15:24.70 --> 0:15:25.290
*Interviewer*
So do they slightly go up?

0:15:24.550 --> 0:16:0.760
*P6*
Yeah, they are like, literally little grizzly things. It's like the top and the side in everything. I think I've worn barefoot shoes normally, which I think has made my feet wider, which is good for your foot health but it just means that, yeah, I bought some Bonts in an Asian fit on the advice of a fitter and they just my foot, the shape of my foot just doesn't work, so I've got a pair of Lakes that I tried out today and I think they're actually pretty, pretty perfect.

0:16:1.830 --> 0:16:5.430
*Interviewer*
On a total side note, I've got wide feet, and I got custom wide Lakes.

0:16:6.390 --> 0:16:6.770
*P6*
Cool. OK, good.

0:16:8.890 --> 0:16:18.820
*Interviewer*
So in terms of saddle, how did you get? I think, did you say you've got on the glider saddle? How did you arrive at that saddle if you've been through multiple iterations?

0:16:19.940 --> 0:16:54.840
*P6*
Yeah, I went, I've been through quite a few saddles before that and ended up with that one from a bike fit a few years ago. I think I'm, because my saddles below it I'm kind of getting used to the feel of it, but out of all the saddles I've tried, it seems to be the one that agrees with me the best and doesn't give me saddle sores. Well, some, yeah, I think, I think it's pretty much pretty much the one and it's on all my bikes. So at least it's the same feel and fit on everything.

0:16:56.370 --> 0:16:59.450
*Interviewer*
And do you ride it flat or do you have a slight tilt nose down?

0:17:0.70 --> 0:17:2.680
*P6*
A tiny little bit of a tilt down.

0:17:4.200 --> 0:17:8.470
*Interviewer*
And you I am right, you don't time trial on it, do you? Don't time trial at all.

0:17:8.830 --> 0:17:13.10
*P6*
Nope, no.

0:17:8.700 --> 0:17:20.30
*Interviewer*
So you don't sit in the really horrible position and in terms of your current saddle you said probably the one, do you ever suffer from any saddle sore issues?

0:17:20.580 --> 0:17:37.210
*P6*
Yeah, you know what occasionally. But I stopped shaving, and actually that seems to have cleared it up, which, I don't know why I read about it, but I just thought it's gotta be something because before it just kept on getting saddle sores and it was just horrendous.

0:17:37.800 --> 0:17:40.980
*Interviewer*
Were they more like kind of little spots type saddle sores?

0:17:41.410 --> 0:17:53.340
*P6*
Yeah. And then kind of build up into the skin into like a I don't know what you call it, like a little cysty thing. And those. Yeah, I don't have them now, so it must be, must be why.

0:17:54.90 --> 0:17:57.340
*Interviewer*
No. Yeah, it is advice from British Cycling not to shave.

0:17:57.870 --> 0:18:0.10
*P6*
So yeah, game changer.

0:18:0.550 --> 0:18:5.990
*Interviewer*
It was developed before the Rio Olympics, with the likes of Laura Kenny and stuff, a big project, so yes, it's the right advice.

0:18:6.600 --> 0:18:7.40
*P6*
OK, good.

0:18:7.940 --> 0:18:19.840

*Interviewer*
Do you ever have any kind of, I suppose probably it sounds like it resolved itself, but if you ever had to have time off the bike for any kind of saddle sore issues, or modify training?

0:18:20.860 --> 0:18:38.630
*P6*
Probably just ridden through it, so. Yeah. And occasionally I get worse, and if there's, like, must be a nerve in your bum or your pelvis, that must hit part of the saddle and you get, like, a weird nerve pain in it. I get that occasionally, but it seems to have settled down.

0:18:39.430 --> 0:18:42.290
*Interviewer*
Is that like a nerve pain down the inside of your leg or?

0:18:42.590 --> 0:18:52.480
*P6*
I just like, right? We're on the inside of like your bum cheek on your pelvis, and it must, there must be a nerve there that just gets irritated.

0:18:53.950 --> 0:18:57.380
*Interviewer*
Is it always one sided, or is it? Or can you get on either side?

0:18:57.960 --> 0:18:58.780
*P6*
I think it's either.

0:18:59.340 --> 0:19:2.80
*Interviewer*
OK. So, it's not like an imbalance type issue.

0:19:7.410 --> 0:19:21.980
*P6*
No, although strangely, the saddle that's on might be the wattbike the back of it rubbed completely, like rubbed through the leather on one side, but not the other side. So, I do wonder if there's something not quite right that I probably need to get checked.

0:19:23.10 --> 0:19:26.570
*Interviewer*
Have you ever seen a physio or anything about that?

0:19:25.860 --> 0:19:46.860
*P6*
Yes, so my wife a chiropractor so, she's looked at various things and I think I'm probably just a bit weaker on the left because of the knee injury. So just trying to like straighten everything out a little bit.

0:19:49.10 --> 0:20:4.300
*Interviewer*
In terms of we talked about saddles, have you ever done anything else in terms of like shorts or you're on team shorts and you just have to ride them regardless? Or if you've got any kind of specific brands that you pick regarding around the chammie or chammie cream to help?

0:20:4.380 --> 0:20:37.690
*P6*
Yeah, there's some, some of the cheaper shorts with a really light thin pad or the pad the, you know, when the like the pad moves. It's not, I hate those. But the kit that we've got for the team the shorts are beautiful it is a women specific pad, but I'd say they are they are so comfortable and which is quite fortunate.

0:20:36.50 --> 0:20:38.990
*Interviewer*
Do you know what they are?

0:20:39.340 --> 0:20:43.800
*P6*
No, I think the kit manufacturer supplies it to some of the big teams.

0:20:44.30 --> 0:20:44.410
*Interviewer*
OK.

0:20:43.810 --> 0:20:46.890
*P6*
So I think it's what INOES wear, but I don't actually know what the brand is.

0:20:47.210 --> 0:20:48.330
*Interviewer*
OK, no worries.

0:20:48.90 --> 0:20:50.380
*P6*
But like, yeah, there very comfortable.

0:20:52.210 --> 0:21:0.400
*Interviewer*
That's kind of the end of my questions about this. Have you got anything else to add around bike fit, set-up, injuries?

0:21:6.960 --> 0:21:49.320
*P6*
No, I just, I say probably, just, the like the lower I think some of them are linked to hormones cause they're definitely cyclical definitely ,like the lower back pain and then that triggers of the things. So, there's probably, oddly if wear bib tights that are, you know, like the waterproof ones or the windproof ones, that the materials not that flexible always gives me knee pain because I think it must, I don't know if the material then stops my kneecap tracking how it should and I think oh no, I'm injured and then I wear something different and it's gone.

0:21:50.150 --> 0:21:52.810
*Interviewer*
OK. That's interesting.

0:21:50.590 --> 0:21:56.50
*P6*
So strange bike, yeah, bike related thing.

0:21:57.850 --> 0:22:8.510
*Interviewer*
And in terms of the low back pain you said you think it is probably hormone related, so is it ever related to like time on the bike? So, like longer rides, does it ever get worse or is it more just, I think time of the month?

0:22:9.180 --> 0:22:49.410
*P6*
Yeah, it's. I don't know. I think I had a period of doing heavier lifting that maybe was a bit of overuse but it was maybe heavier lifting it and then hormones on top of it, I think. Time on the bike probably no, because I could went out this morning for four hours at the end my back may be a little bit but not more than you just stretch off by standing up. It doesn't hurt now, so I tend to think it's probably more hormones than. Yeah. So, I think, I think that's the, I think that's the cause of it.

0:22:50.580 --> 0:23:3.680
*Interviewer*
OK, great. And then the one final question, are there any areas you would like research in regarding to female cycling? So maybe menstrual cycle, specific training for females, bike setup, saddle choice, injuries?

0:23:4.940 --> 0:23:40.580
*P6*
You know what I think saddle choice and like saddle health cause it's, sometimes you don't. I don't really know what to do. Like what? chammie cream or Llike what should I wash with? Should I get like there's loads of stuff that it's a bit difficult to work out? Like what’s the best way to not get saddle sores and when you do like what. Because it's so difficult to you know, we ride through so many injuries and so much discomfort you kind of think, yeah, I think that's a little bit where I don't really know where to go for the for like the knowledge.

0:23:42.370 --> 0:23:44.0
*Interviewer*
And is there anything else or?

0:23:46.290 --> 0:24:36.560
*P6*
Probably. I think the bike fit aspect because so maybe so much of the research and the literatures all about men and like, we're not, women aren't little men, like we're, just the dimensions of this is so completely different. You know, even when I'm talking to my coach about something and it's like, yeah, it's quite a, you know, it's like, why, why are you riding your bike with tiny wheels and like my feet knock the wheel if I turn it ,and you know. Yeah, I think the yeah, even like reach, I think I've got quite long legs in a short body, but even like pelvis size like even a little like a small woman like some women got really wide hips and it must still affect bike fit and stuff like that.

0:24:38.650 --> 0:25:7.390

*Interviewer*
So that is one of the things that this work is some of it is related with British Cycling and one thing even on the elite riders, there's differences in how you can tilt your pelvis, particularly in the more aggressive positions. Women can't, they don't have the same flexibility men do, so we can't get into some of the same positions. So yes, it's not you. It's known that we can't get into and also the lower back pain and the hormones is, yeah, there's stuff showing pelvic tilt can change throughout the menstrual cycle phase.

0:25:8.140 --> 0:25:8.400
*P6*
Wow.

0:25:8.0 --> 0:25:21.20
*Interviewer*
So yeah, but it's not out there. People don't really talk about it, so. OK, that's great. That's all for me, unless you've got anything else you'd like to add.

0:25:21.750 --> 0:25:22.570
*P6*
No, I think that's cool.

0:25:22.420 --> 0:25:23.280
*Interviewer*
OK, great.

**P7 – interview transcript**

0:0:0.500 --> 0:0:8.100
*Interviewer*
So just start with a bit of background about you and cycling. Have you always been a cyclist, or have you done other sports as well?

0:0:9.210 --> 0:0:54.730
*P7*
OK, so I've been cycling since I was eight years old. So, from seven years old. I practice competitive cycling and then from the youth categories I moved to triathlon. So, uh, now I'm doing both triathlon and cycling. So yeah, triathlon and running and swimming separately. But the sport where I feel more confident is cycling…

0:1:48.710 --> 0:1:52.240
*Interviewer*
How is your training going towards those races at the minute?

0:1:54.70 --> 0:2:30.670
*P7*
Uh, so I'm training for cycling four times a week. So, usually three times it's like one hour, one hour and a half with more specific training such as intervals or tempo and so on. And while in the weekend I do more steady cycling, so like 3-4 hours, but it usually depends also on like the weather or something like this.

0:2:32.340 --> 0:2:41.720
*Interviewer*
And going on to your menstrual cycle, thinking back to when you got your first period and what was that like for you? How old were you at the time?

0:2:43.150 --> 0:3:13.80
*P7*
I was 14 years old, but like the first year it was very irregular. Also, because my weight was very low and so yeah, for the first like 2 years, it was very not regular. I mean and then, but I've never had like symptoms associated with menstrual cycle.

0:3:15.320 --> 0:3:28.310
*Interviewer*
So when you first started because obviously you mentioned you are now on oral contraceptives were you playing sport when they first started for well you would have been cycling. Did that have any effect on cycling or wanting to do training?

0:3:29.550 --> 0:3:31.120
*P7*
Uh. The oral contraceptive?

0:3:31.190 --> 0:3:33.110
*Interviewer*
No, starting your period. Sorry.

0:3:33.310 --> 0:3:50.980
*P7*
OK. Uh, no. I like, I feel like when it comes, I don't mind. I mean it doesn't affect any, yeah, the performance or also how I feel.

0:3:52.930 --> 0:4:4.590
*Interviewer*
And you mentioned when you first started because you were quite, your weight was quite low that they were a bit irregular. Did you get any advice or information around that time about periods or menstrual cycle?

0:4:6.330 --> 0:4:53.610
*P7*
No, but I was, I had like a nutritionist and my doctor told me that I had to increase a little bit my weight because it was really low and was it also a problem for like performance also because when I, like move from different categories like the distance became like longer and so on. So, I had to take more care of diet, yeah.

0:4:55.380 --> 0:5:3.670
*Interviewer*
And so is I said you shared you on contraceptives, do you experience withdraw bleeds and are these regular?

0:5:6.80 --> 0:5:8.600
*P7*
Uh, if it becomes more regular?

0:5:9.320 --> 0:5:13.380
*Interviewer*
So do you experience withdraw bleeds on the oral contraceptive pill?

0:5:14.840 --> 0:5:18.320
*P7*
Uh. What do you mean?

0:5:19.10 --> 0:5:25.930
*Interviewer*
So you take the oral contraceptive pill. Do you get a withdrawal bleed every month?

0:5:26.640 --> 0:5:28.810
*P7*
Uh, uh. Yeah, yeah.

0:5:28.680 --> 0:5:35.700
*Interviewer*
And from what you said, that has no impact on your training. You don't make any modifications around that.

0:5:37.250 --> 0:6:0.850
*P7*
No, I had like, I had no symptoms during like a normal menstrual cycle. And also, when I began to take the oral contraceptive. So yeah, I felt like better because it was regular, so I know when it comes so.

0:6:2.720 --> 0:6:16.40
*Interviewer*
And I think you mentioned on your form the original reason to start taking the oral contraceptive pill was due to acne. Was that recommended by a doctor at the time?

0:6:14.470 --> 0:6:20.150
*P7*
Because I, yeah. Because I went to the doctor of the skin.

0:6:20.710 --> 0:6:22.70
*Interviewer*
Yeah, dermatologist.

0:6:22.300 --> 0:6:42.820
*P7*
Yeah. And they, I tried different types of cream and antibiotics and so on, but it didn't work. And when I started the pill and like the acne disappeared.

0:6:49.30 --> 0:7:8.570
*Interviewer*
I think kind of had a chat, but obviously you do some quite long-distance racing, so 100 kilometres if you're on your like a withdrawal bleed happens during that time. Do you have to take any extra precautions or is it the bleeding quite light and you don't have to worry for 100K that you'll be OK?

0:7:9.770 --> 0:7:28.180
*P7*
Yeah, it's very light. So, like it doesn't affect the race Yeah, I don't, I don't have to take extra measurement for it, yeah.

0:7:29.740 --> 0:7:43.120
*Interviewer*
As a female athlete, obviously you mentioned you had any nutritionist when you were younger. And obviously I think you've had coach in the past. Have you had any advice specifically around the menstrual cycle and training or contraceptive use?

0:7:45.660 --> 0:7:48.360
*P7*
From the nutritionist or from the?

0:7:47.370 --> 0:7:54.560
*Interviewer*

From like anybody who supported you. So, coach nutritionist, any of those practitioners?

0:7:54.460 --> 0:8:5.820
*P7*
No, they like, didn't, they never mention like the menstrual cycle. So no, they didn't take it into consideration.

0:8:7.170 --> 0:8:18.570
*Interviewer*
And in terms of feeling comfortable talking about it, are you, would you be comfortable I know you self-coach now, but would you be comfortable having conversations around the menstrual cycle with a coach or with anybody else?

0:8:19.690 --> 0:8:26.330
*P7*
Uh, yeah. When I was like young or.

0:8:23.330 --> 0:8:27.660
*Interviewer*
Now, and yeah, maybe talk about when you're younger as well.

0:8:29.160 --> 0:9:58.200
*P7*
No, like during my competitive seasons. Like during when I was like younger and so on that I had some coaches I never talk about the menstrual cycle. Maybe also because they were males. But I had like 2 years for two years, I had a female coach and with her I've never had problems with the menstrual cycle, but some of my teammates had and so they feel they felt free to talk with her like it was easier to talk with like a female coach about their period instead of like male. Also because it seems that sometimes they were also like old coaches and so like they didn't want talk about this.

0:9:58.270 --> 0:10:40.430
*Interviewer*
Obviously cycling is a very male dominated sport even now in terms of coaches, team managers. I am obviously I'm talking about this stuff based normally for UK riders. I'm not sure what the situation is in Italy but are you aware that kind of more elite athletes are discussing their periods in the media. So, in the UK we had a couple of big athletes, one was Dina Asher-Smith sprinter who spoke about her periods affecting the 100 metre final I think it was a European Championships and Eilish McColgan the distance runner talking about the influence of periods. And are you aware there's more, I suppose conversation going on in media, social media around these issues?

0:10:42.10 --> 0:10:43.820
*P7*
Uh, no.

0:10:45.280 --> 0:10:49.300
*Interviewer*
So in Italy, do female athletes not talk about it at all?

0:10:51.810 --> 0:10:53.940
*P7*
Uh, I don't feel it. I mean, I, nowadays that like social media and so on. It's easier to find people who talk about it, but I mean in terms of like cycling federation or in the teams for example, like, I don't feel like they talk about it.

0:11:24.330 --> 0:11:46.600
*Interviewer*
And to my knowledge, I haven't heard any female cyclists talking about this issue and maybe a little bit like Laura Kenny is talking a bit about it, but that I think around yes, about it. That's all my questions related to the menstrual cycle. Do you have anything you'd like to add or think I've missed asking you about?

0:11:49.370 --> 0:12:41.230
*P7*
No, it's only that I think it depends a lot on your coach. I mean, if you like, feel free to talk about it or if you feel judged or. Yeah. And I think that probably a female coach and like she knows what it means, and so and maybe. Yeah, she's more aware of what's happening that, some athletes could have some problems related to menstrual cycle and others not so.

0:12:43.530 --> 0:12:53.810
*Interviewer*
So some moving on to the next section. In terms of the bike you ride, I think you ride a women's specific frame, is that correct?

0:12:52.990 --> 0:12:54.350
*P7*
Yeah, yet.

0:12:54.20 --> 0:13:2.10
*Interviewer*
Have you have you made any modifications to the bike setup since you bought it to suit your position like handlebars, cranks?

0:13:2.770 --> 0:13:38.960
*P7*
OK. For the bike I have now, no, because it's like women specific and it like my size because I'm short so in the past I found very difficult to find my right size, so I had to do a lot of modification. But now, yeah, I have my bike and I don't want to leave it.

0:13:37.450 --> 0:13:49.680
*Interviewer*
yeah, once you get it right. Have you suffered from any cycling overuse injuries so things like lower back pain, foot pain, knee pain and hand or finger numbness or saddle sores.

0:13:51.320 --> 0:14:38.980
*P7*
Yes, saddle sores. Yeah, and but it it's like, uh, also when I started cycling and I thought it was normal to have a saddle sores, but I tried different type of saddles and now I found my more or less right saddle, but I think I've like I've never had injuries, but I think the most. It's not injury, it's like a sore, which is like the saddle. Yeah, it was the most.

0:14:42.430 --> 0:14:53.130
*Interviewer*
In terms of like finding your saddle, was it just a case of trial and error? So, try and different saddles and seeing if it was comfortable or did you get a bike fit to help with that.

0:14:52.870 --> 0:15:24.960
*P7*
Yeah. Yeah, I went to, I did some bike fit and they, and they suggest me a type of saddle because they had a system and where I had to, like, sit on this. I think it's like you have some sensors in this. I don't know how it's called.

0:15:24.20 --> 0:15:31.320
*Interviewer*
Was it like, was it either like a bit of foam to measure your sit bone width or was it measuring the pressure on the saddle?

0:15:30.540 --> 0:16:7.50
*P7*
Yeah, the pressure and also like the distance between the ischial bones and I had also to make like a movement of the back like a flexion and extension to see how was my mobility. And then I did the same, like, on the bike and so they measure like the pressure in on the front end on.

0:16:10.870 --> 0:16:11.740
*Interviewer*
And is this the saddle you have now.

0:16:12.580 --> 0:16:31.730
*P7*
Uh, no. I mean it's similar but now I have a more like a shorter saddle. So, the type is very similar, but like the it's only shorter.

0:16:35.200 --> 0:16:47.0
*Interviewer*
In terms of suffering, you said you suffering saddle sores. Is there anything that makes that worse or more likely to happen, like length of ride, going on the turbo, position?

0:16:48.250 --> 0:17:44.60
*P7*
Yeah, it's more like how much, how many hours I stay on the saddle. Yeah it's like more the length then like the position, but I think like umm, because I have two bikes with the same saddle on the two bikes. But with one bike, so not the best one, I mean, I feel more uncomfortable, but the saddle is the same, but probably the position is different, and I think so I think that's also the position influence how you feel on the saddle, not only the saddle itself.

0:17:45.130 --> 0:18:4.780
*Interviewer*
So yeah, if you've got a tilt, a drop to the handlebars. And you said you said from suffer from saddle sores, what type of saddle sores are these like from pressure, do you get chafing, do you get a little cyst like spots or and yeah.

0:18:4.830 --> 0:18:39.430
*P7*
Yeah, it depends. It depends on the saddle. So, with some saddles, it was like a matter of pressure and with others like the bike I had on track, it was more like. Some I don't know. I have to code like some signs. With some blood also.

0:18:36.50 --> 0:18:54.400
*Interviewer*
OK. So, you like you broke the skin and had a little. Yeah. OK. So yeah, maybe like chafing related and in terms of where the saddle sores were with these sit bones, upper thigh or more like kind of labia, outer labia, inner labia, and kind of front of the saddle?

0:18:56.170 --> 0:19:21.240
*P7*
Allso in this case depends also on the saddle. So, for the pressure, it was like more on the front. And while for the skin it was more on lateral but yeah, it really depends on the saddle.

0:19:23.360 --> 0:19:37.840
*Interviewer*
In terms of getting saddle sores, what do you do if you get a saddle sore? What you do to treat it? Do you just have time off the bike? Have you ever had to get medical help or kind of use painkillers, or like often repair cream?

0:19:38.710 --> 0:20:21.60
*P7*
Yeah, yeah, I use like the cream. And but I've never had like to rest too much. I mean, because if it was a very big sore, I change like I did something to change the saddle or the position because otherwise I couldn't train. Because and sometimes I had some sore, but it was manageable. So, I didn't care too much.

0:20:23.930 --> 0:20:35.430
*Interviewer*
And has it ever you've mentioned there that if it got bad, you'd make some modifications, but has getting a saddle sore affected your ability to train and compete or influence your enjoyment of cycling?

0:20:37.470 --> 0:21:7.700
*P7*
Yes, sometimes it more than like performance or a so on it influences like my well, so maybe you want to finish your training because of the saddle not because your fatigue. So yeah, it was more like uncomfortable than like affecting performance, I think.

0:21:9.620 --> 0:21:14.460
*Interviewer*
And when you were a coached rider, did you ever discuss if you had a saddle sore with a coach? Did you mention it to them if it was affecting you?

0:21:25.230 --> 0:21:43.700
*P7*
Probably I think yes, but they didn't care too much, so I tried to solve it myself. Yeah, because maybe they think it’s OK, it's normal so.

0:21:45.930 --> 0:21:59.590
*Interviewer*
In terms of trying to prevent saddle sores you've mentioned, like you've done quite a lot to try different saddles. Is there anything else you've done? If you changed your position, have you changed your shorts or use of chammie cream any of?

0:22:5.970 --> 0:22:37.260
*P7*
Yeah, I found like a cream which is useful. And also, I changed the shorts, or maybe I had new shorts and they found they were more comfortable, so I tried to maintain always the same so it's more. Oh well it's the right saddle, or wow I found the right short, so I tried to maintain always the same just because it's better than the others.

0:22:40.150 --> 0:22:47.870
*Interviewer*
That's all my questions on kind of saddle sores and bike set up have you got anything else you'd like to add, or you think I've missed asking you about.

0:22:50.90 --> 0:22:52.430
*P7*
No. It’s OK.

0:22:53.420 --> 0:23:10.230
*Interviewer*
Final question, are there any areas you would like researching, in regard to female cycling? So menstrual cycle, training, bicycle setup, saddle choice, injuries.

0:23:8.0 --> 0:23:10.300
*P7*
Uh, sorry I didn't catch the question.

0:23:10.270 --> 0:23:22.880
*Interviewer*
So are there any areas you would like researching in female cycling? So are you around the menstrual cycle, training for females, bicycle setup, saddle choice, injuries.

0:23:24.310 --> 0:24:32.520
*P7*
Yeah, I'm interested in all these areas. So also, because like for the saddles or for also the bike I mean the size of the bike, I think there's not so much choice compared to males. So also, when I was like younger, and I couldn't take like a female saddle or female frame for example. So maybe now it's better, but I think there are, there is a lot of to do because you have not like so much choice compared to men. So, also because like the anthropometry is different with and also, like uh the height is different, the pelvis is different. So, I think, yeah, there's a lot to do.

0:24:34.900 --> 0:24:45.10
*Interviewer*
That's great. Is there anything else you'd like to add before I turn the recording off?

0:24:47
*P7*

No

0:24:48
*Interviewer*

OK. Thank you.

**P8 – interview transcript**

0:0:0.0 --> 0:0:7.600
*Interviewer*
So just a bit of start about you and cycling and how long have you been cycling and what events do you compete in?

0:0:8.820 --> 0:1:17.780
*P8*
I started cycling ten years ago, but I was doing triathlon and I wouldn't have really said it was a cyclist at the time but when I went to uni, so eight years ago, it was when I properly started cycling. So, I did triathlon and that kind of led to time trials. So, I did time trials from 18 to age of 21 and then for, then I did a season of cyclocross and then that was 2019-2020 season and then I was just time trialling because that's all that was available. Did a bit of cross the following winter and then in 2021. What I'm trying to think now, what were. 2022 I did my first road race and I've done a handful of crits before, but not really anything properly, so 22 I did my first road race, a couple of crits and then last year I did a full season of road racing. So yeah, sorry…

0:2:2.360 --> 0:2:11.250
*Interviewer*
So going on to your menstrual cycle, thinking back when you first started getting the period, what was it like for you? How old were you at the time?

0:2:12.390 --> 0:2:37.440
*P8*
I think I was twelve. I'm pretty sure I was 12 or 11, like late 11. I don't really remember what it was like. It was never horrific, horrific. Like I certainly got period pains, but I'd say it was probably four to five days when I was a teenager, I think, yeah.

0:2:37.450 --> 0:2:41.820
*Interviewer*
And were you playing sport when that happened? Did that have any effect on you playing sport?

0:2:43.100 --> 0:3:11.970
*P8*
Yeah, when I first got my period, I wouldn't swim because I used to. I did quite a bit of sport as a kid and so I wouldn't swim and then it didn't really stop me. But I don't, I don't think I didn't go ever, but I did judo and jujitsu and I remember like getting out of the session a few times and like, obviously you wear a white suit and being like oh no. So yeah.

0:3:13.190 --> 0:3:22.550
*Interviewer*
At that time, did you get any kind of educational advice around periods or menstrual cycle? Was it just the standard school chat?

0:3:21.330 --> 0:3:40.100
*P8*
So standard school. I remember when we were in, I think it was year six, which I think is quite early. We got shown how a tampon works, even though I only think like a couple of the girls started their periods. So, guess it made sense, but other than that it's just standard school stuff, yeah.

0:3:41.150 --> 0:3:46.790
*Interviewer*
And as you've got older, has your menstrual cycle stayed the same or your symptoms changed?

0:3:47.990 --> 0:4:29.910
*P8*
Changed quite a lot when I went on the pill. I was in the combined for a while and I think they were like fairly standard on the combined. And then I went on the mini pill. I lost it for a year and then I changed which progesterone it was, and then I spotted for like 9 months because they said it would go, but it didn't. And then I changed the progesterone again and I think it went back to no spotting, just a normal period but then I just couldn't be assed for that anymore. So, I went off the pill and then since then it's been really standard. Every 28 days. Three days so.

0:4:27.840 --> 0:4:33.120
*Interviewer*
It sounds like from your questionnaire that you had some pretty horrific symptoms off the pill.

0:4:36.580 --> 0:4:37.490
*P8*
It's not my favourite.

0:4:34.290 --> 0:4:43.360
*Interviewer*
Was that ultimately what made you decide to stop using like the pill?

0:4:44.150 --> 0:5:28.150
*P8*
Yeah, like I'd had side effects of the two progesterone types. Obviously that the first one, I didn't have a period I really liked because I didn't have a period and but then obviously, so I got a lump and then I just had constant boob pain. So, I just like, OK, can't do that anymore. And then the other one was spotting I was like, no. And then the last one, I kind of had seen a bit about how you're not yourself on hormonal contraceptives and I was like, I think it was during lockdown and I'm in a long-term relationship and he's literally like, I'll do whatever's best for you. It can't be great, and I've done a tiny bit of reading about it, and I was like, oh, I just can't be bothered anymore. So, I stopped.

0:5:29.910 --> 0:5:37.430
*Interviewer*
In terms now obviously back on a natural cycle and it sounds like it's pretty regular. What symptoms you get now with your period?

0:5:38.950 --> 0:6:2.900
*P8*
Period pains like cramps in my stomach and back on the day before and the first day, occasionally a little bit on the second day but generally that's about it. I mean, it's rare, but sometimes they're a bit like, it can be a bit worse and I'll get, like pain in my tops of my legs as well. But yeah, other than that its kind of it.

0:6:3.780 --> 0:6:7.710
*Interviewer*
Do you get any kind of emotional symptoms like changes in mood or irritability?

0:6:9.150 --> 0:6:11.980
*P8*
Yeah, like the week before. I'm like, yeah, pretty.

0:6:11.610 --> 0:6:13.200
*Interviewer*
So that kind of premenstrual phase.

0:6:13.970 --> 0:6:14.590
*P8*
Yeah.

0:6:15.920 --> 0:6:20.60
*Interviewer*
In terms of that, do you ever have to take pain medication for your period pains?

0:6:21.0 --> 0:6:28.830
*P8*
Yeah. I tend to take paracetamol the first day of, when I definitely know that is, even though I can predict it and I know its period pain the day before I will only take it on the day.

0:6:30.310 --> 0:6:52.300
*Interviewer*
Yes, once it happens. Then in terms of managing periods around training and competition, say if you're on your period, does that stop you going on off for a long road ride? If you need a toilet, stop to change sanitary protection. Or are your periods of light and is that not ever a consideration?

0:6:49.750 --> 0:7:10.40
*P8*
I use menstrual cups, so I don't like. And to be fair, I think if I used to, I had to use a tampon. A couple of months ago because I forgot my menstrual cup wherever I was, and I was like this horrible. And I don't like cycling with one in, so maybe it would be different if I did, but yeah.

0:7:11.90 --> 0:7:18.40
*Interviewer*
Do you ever suffer from any tiredness around your period or take any extra recovery? Or do you just kind of get on with it?

0:7:19.550 --> 0:7:46.100
*P8*
Kind of get on with it. I have done research around menstrual cycle, so I do train with it a bit and my coach was pretty good with that. So, we have some like recovery-ish the week build up. On the actual period, unless I'm feeling really crap. So, very occasionally, I won't train on the first day of my period, but usually I just crack on.

0:7:47.310 --> 0:8:2.600
*Interviewer*
Is there any difference between, obviously, you've said you time trial and obviously time trial and is pretty aggressive position. Does that ever affect like period pains or bloating or anything ever affect you going into TT position or you're just trying to avoid it on the turbo?

0:8:4.20 --> 0:8:11.910
*P8*
I usually just crack on with it, like its back pain anyway. So just yeah.

0:8:13.470 --> 0:8:26.170
*Interviewer*
Do you ever experience as any changes in kind of your coordination, core, strength, mobility, or flexibility at any point during your menstrual cycle or maybe balance?

0:8:27.820 --> 0:8:30.220
*P8*
Not that I've picked up on.

0:8:31.10 --> 0:8:47
*Interviewer*
Yes, some people do, some people don't. You mentioned there that your coach is pretty good and you maybe you slightly train around the menstrual cycle. So, I presume you're happy having conversations with your coach about how your periods are affecting your training?

0:8:47.710 --> 0:8:47.910
*P8*
Yeah.

0:8:47.870 --> 00:8:54.770
*Interviewer*
Do you share your tracking data, or do you just put a note on training peaks or whatever to let your coach know how you're feeling?

0:8:53.790 --> 0:9:3.370
*P8*
I put on intervals. You can add the date of your period, so I just I'm on.

0:9:3.120 --> 0:9:19.360
*Interviewer*
Ok, so you just let them know that way. In terms of competition, have you ever, would you just crack on if it was a competition and your period started, or have you ever had to not race or felt you performed worse than ideally you would like to?

0:9:20.660 --> 0:9:39.140
*P8*
I always just crack on. I'm not necessarily ever blamed on my period. I don’t know if it could have been, but usually I just fall into a pit of self-loathing and stay.

0:9:40.100 --> 0:9:45.620
*Interviewer*
Yeah, but you haven't like tracked you period and how you performed. You don't like objectively look at that?

0:9:46.420 --> 0:9:46.680
*P8*
No.

0:9:48.420 --> 0:9:57.550
*Interviewer*
I am in terms of you mentioned you've done some research around the menstrual cycle. Is that just online or have you had any like kind of more formal education advice on this?

0:9:58.380 --> 0:10:0.60
*P8*
Just online and reading books.

0:10:1.140 --> 0:10:5.950
*Interviewer*
As you coach ever recommended anything to like information to read or look at.

0:10:6.740 --> 0:10:7.720
*P8*
Yeah, yeah.

0:10:9.140 --> 0:10:24.500
*Interviewer*
And are you aware that there's been for the last couple of years in the media, there's been a lot more talk about periods and elite athletes - Dina Asher Smith spoke up about it, Eilish McColgan. Has that influenced you maybe doing research on this topic, wanting to find out more?

0:10:26.90 --> 0:11:2.770
*P8*
I think I saw I was like already reading about it just before that stuff. I remember the Dina Asher-Smith interview. Like and I'd already done a little bit of research prior to that. So, I think I started training around my period in 22 early 22. So, yeah, two years ago. I didn’t see anything specifically convince me but like I was already doing research, but if I see stuff, then I’ll go and research more about it if there's.

0:11:5.410 --> 0:11:34.870
*Interviewer*
Obviously you said that you started training in 22 around your period was that prompted by you doing research, or was that prompted by your coach introducing it? Sorry P8 did you disappear there, or did it freeze? Sorry, I'll repeat my question. When you said you started training around your period in like 22, was that prompted by you doing research or was that your coach suggesting it was a good idea or a joint discussion?

0:11:34.410 --> 0:11:51.830
*P8*
My coach is a rowing coach for female like female rowers and he, I think was doing some stuff with them. And so, it kind of said to me like, oh, we don't have to, but I don't if you be interested in this, so I read about it and I was like, yeah, definitely. So that's when we started.

0:11:54.180 --> 0:12:8.210
*Interviewer*
OK, great. Another question talking about your coach, you're quite comfortable talking with your coach. Are you comfortable talking with your teammates or your team manager about periods or does it just depend?

0:12:9.40 --> 0:12:36.970
*P8*
It kind of depends. There's nothing that would stop me, but also, I'm very aware that other people aren't always comfortable talking about it. So sometimes I'll like hint towards it, but if people aren't interested, and maybe if I got to know a bit better because they're quite new, I've only met them a handful of, so maybe further down the line. But yeah, once I've got to know someone, I don't really have much of a filter so.

0:12:39.240 --> 0:12:48.590
*Interviewer*
That's all my questions around the menstrual cycle. Have you got anything you wish to add? Think I've missed in my questions around that topic.

0:12:51.70 --> 0:12:53.50
*P8*
I don’t think so.

0:12:53.900 --> 0:13:6.540
*Interviewer*
Ok, I’ll go on to the second part. So, in terms of, I think from reading your bikes, I think none of them, they're all kind of unisex if you wanna use that, they're not. You don't write any women's specific, is that right?

0:13:7.170 --> 0:13:9.550
*P8*
So far as I am aware I think that.

0:13:9.280 --> 0:13:15.910
*Interviewer*
And if you made any modifications to those, since you bought them like handlebars, stem, anything like, that to suit your position.

0:13:16.760 --> 0:13:21.200
*P8*
Yeah. Yeah, sorry do you need a list or?

0:13:21.410 --> 0:13:25.110
*Interviewer*

Yeah, if you want to say what you've changed, that would be great.

0:13:25.740 --> 0:14:15.10
*P8*
Stem length changes across all of them, generally a bit shorter. I don't know if that's because I'm short or because I just like the handling with it shorter. TT bike obviously has the most adjustments. I'm not sure how long I think the stem is, really short actually, but it's quite an aggressive position. My drop from my saddle to my bars is, I don't think there really is much of one on my TT bike. Saddles, there all the same now, but they've changed quite a bit through the years. But other than that, I have 165 cranks, so yeah.

0:14:15.800 --> 0:14:24.370
*Interviewer*
In terms of saddle, you said it's changed over the years. How did you arrive at your current saddle? Was it like trial and error, or recommendations, bike fit?

0:14:25.840 --> 0:16:22.520
*P8*
Trial and error really. I was on, I don’t even know I was on then. My dad is a bike mechanic and really into bikes. That's how I got into it. He recommended a women specific Selle Italia one and I was on that for quite a while, but it's quite thick padding, it's, I think I've always been on ones with a gap in the middle. So then I was on that for a while, changed to a Fizik deluche – maybe, it's the women's one that's wide and I've had one of those things where you sit on the it's like a block and you sit on it, and it's got like padding in it and it shows you where your sit bones sit. Yeah. And that's why I got the wide one. So, it's on that for a while, kept getting saddle sores and I think my TT bike I had one of the ISM, two prong ones and I kind of just accepted that you had to be in pain on a TT bike which was kind of crap. But, yeah, I just thought that was how it was. And then I changed to the Prologo ones out of luck, I think when I got my cross bike. Oh no I was on a luche for that as well. I think, I don't know how I ended up on the Prologo on it was just I think my dad said oh that one looks like you, you know quite good. I like it, so I had to sit on it and I was like, this is really comfy. So I got it on my road bike and then we were talking about my TT bike and then I just put it on that as well and I was like, no way is this more comfortable because it's just a cut out again it's not the two prong, but I think because it's thinner and the two prong always felt like I had two bits of metal digging into me. So yeah, that's how I ended up with that one just trial and error.

0:16:23.810 --> 0:16:45.970
*Interviewer*
Yes, so talking about saddle sores. In terms of you said about the two prongs and quite wide, where were you getting saddle sores? Was it like upper thigh? Was it outer labia, inner labia? Was it kind of front end from sitting on the TT bike?

0:16:38.830 --> 0:17:36.690
*P8*
Uh, usually I'd get them outer labia like groin, like, right on that. And they'd be, like, hard internal cysts, like, that's certainly the saddle sores I always get. I still get them occasionally they're always there. After I while we worked out that I have one leg shorter than the other, so I always had saddle sores on one side. Now I've got a shim on my pedal. I get them on both sides, but less regularly. And then a very occasionally now I'll get them like quite far up like bikini line. It's almost like an ingrown hair that gets aggravated. I don't know if it is or not, but that's how I would describe it as it was almost starts as an ingrown hair kind of size lump and then gets massive because of the friction I guess so yeah.

0:17:36.800 --> 0:17:41.710
*Interviewer*
So is that is that the same sort of thing like a hard cyst, or is that more like a kind of spot?

0:17:43.150 --> 0:18:23.780
*P8*
It's like a hard cyst, but a lot of them have heads occasionally, maybe were it is if there is a hair there or and yeah, and then to be fair, sometimes the ones on the outer labia can be blistery like sometimes they get, so, like I have one relatively recently actually that it got to a point I could tell this skin was really thin, so I popped it at like with a needle and that was like, yeah, blistery. And I have occasionally had a like a slight blister on the inner labia, but that doesn't happen very often.

0:18:25.150 --> 0:18:38.630
*Interviewer*
Yes. And in terms of what brings, you so obviously is much better now you've changed your saddle, but is there anything that makes you more likely to get a saddle sore like long ride, TT’s? Or is there any? Or is it just seems a bit random?

0:18:39.670 --> 0:18:52.80
*P8*
Luck of the draw I think and sometimes I wonder if it's if I haven't, like, pulled my shorts right into my groin or something. Certain shorts can be but nothing particularly.

0:18:54.180 --> 0:19:2.870
*Interviewer*
In terms of treatment for saddle sores, how would you? How would you manage them? Do you have to have time off the bike or do you just kind of push on through or?

0:19:4.910 --> 0:19:22.700
*P8*
Usually I just put sudocream on it and push through. I've had two where I've had two or three maybe that I've had to take time off the bike because it just isn't healing and but generally, yeah, I just hope for the best and hope they will go away at some point.

0:19:23.780 --> 0:19:28.420
*Interviewer*
Have you ever had to get any medical treatment for saddle sores or seen the GP? Or if you just yeah.

0:19:29.360 --> 0:19:29.700
*P8*
No.

0:19:32.500 --> 0:20:0.610
*Interviewer*
In terms of like getting saddle sore apart from me, you said a couple you've had to have time off the bike. Does it affect you training and competing generally, or is it just if it's really severe?

0:20:1.910 --> 0:20:15.900
*P8*
Sometimes I do especially with time trialling where you can’t really shift about on the saddle that much. I have had a few races where I have been like hovering for half of it because you just can’t put weight on that part which hasn't been great. But yeah, like, on the road bike, it's funny the worst one seem to be that I don't even notice them until after I've gotten off the bike. So yeah.

0:20:19.580 --> 0:20:41.700
*Interviewer*
In terms of obviously, you've changed your saddle and you've now got a shim to help address the leg imbalance. Is there anything else you've done to try and help prevent saddle sores like change of your shorts, saddle tilt is another one, and if you had any like kind of physio to kind of treat the ending up on one side, more prominent one side saddle sores.

0:20:43.370 --> 0:21:25.290
*P8*
So I do have a saddle tilt and have had for a long time to be fair. Shorts, yeah I only get pretty, I try to only ride in pretty decent shorts now, like I won't ever cheap out on it. I am. I've never. I went to the physio because I have a dodgy leg but not specifically treat like saddle sores on one side. Yeah. I guess having one dodgy leg like probably leads to having saddle sores on one side, yeah.

0:21:26.270 --> 0:21:36.370
*Interviewer*
In terms of any other cycling overuse injuries, have you ever had problems with like back pain, knee pain, foot pain, or hand numbness or finger numbness?

0:21:37.860 --> 0:21:49.800
*P8*
I get a lot of hand numbness, a lot. I had actually looked it up. I can't remember what it was called now, but it was my like here was all numb from here.

0:21:48.940 --> 0:21:51.570
*Interviewer*
OK, so the ulnar nerve.

0:21:49.810 --> 0:22:15.560
*P8*
Yeah, yeah, I think it's quite common in cyclists. Yeah. So that went numb. It like it's fine now, but it was for a couple of months last year. And yeah, I do lose feeling like get numbness in my hands generally whilst I'm riding and then my feet go a bit not as well most of the time.

0:22:14.950 --> 0:22:22.620
*Interviewer*
Is that like under the metatarsals joint? So kind of where your foot bends on the ball of your foot, is it in that sort of position or?

0:22:23.990 --> 0:22:25.690
*P8*
Yeah, they're and like my toes.

0:22:27.240 --> 0:22:31.660
*Interviewer*
Again, have you done anything to try and treat those or just get on with it?

0:22:32.570 --> 0:22:59.450
*P8*
No, I thought that was circulation, to be honest with my feet and hands. No, I kind of accepted because it only happened last year like I get numbness while I'm out riding and I know that's because of where I'm pressing on the handlebars but the like persistent numbness once I'd googled it I was like oh probably go away because it's a generally you get it when I'm on the drops I think is what caused symptoms.

0:22:56.670 --> 0:22:59.450
*P8*
So yeah. No.

0:22:59.430 --> 0:23:9.200
*Interviewer*
That's all my questions around kind of saddle sores and injuries if there anything else you'd like to add before I move on?

0:23:11.210 --> 0:23:21.710
*P8*
Uh, no, I think oh. In terms of other injuries, I don't think it's the same, but I do, I do get a hip impingement, especially when I'm on the time trial bike, so I don't if that's relevant.

0:23:22.400 --> 0:23:35.220
*Interviewer*
Oh yeah, no, yeah that relevant because that is often a female specific injury. Again, is that does that result in a loss of power in the leg with the impingement? Have you had any treatment for that?

0:23:35.880 --> 0:23:51.550
*P8*
Yeah. Yeah. So, years ago, when I was exclusively time trialling, it was in my right leg, and I had treatment for that. And then that's my left leg is now the one that's really dodgy and that's got an impingement and the whole leg is just a bit crap, so yeah.

0:23:52.340 --> 0:23:55.350
*Interviewer*
Are you still getting treatment for that, or have you just accepted it?

0:23:56.10 --> 0:24:26.270
*P8*
I got treatment last year, went to Physio and he gave me load of exercises to do and it kind of calmed down after I've done for a few months and then over the winter I imagine it's because I'm on different bikes and throughout the winter and then summer I change and I guess the so the setup is slightly different across all the different bikes. And so, it's gotten pretty like it was awful this past week again. So, I'm starting to do all of the exercises again.

0:24:27.380 --> 0:24:33.140
*Interviewer*
Is it just time position or is it also like an aggressive road position that is a problem for?

0:24:33.900 --> 0:24:37.980
*P8*
Aggressive road as well.

0:24:40.390 --> 0:24:52.410
*Interviewer*
Just one final question, are there any areas you'd like research regarding to female cycling so menstrual cycle, training for female athletes, bike setup, saddle choice or injuries.

0:24:54.90 --> 0:24:55.540
*P8*
That I'd like to see researched?

0:24:55.600 --> 0:24:57.70
*Interviewer*
That you would like to see research and yeah.

0:24:58
*P8*

I mean all of it, obviously. Saddle sores and saddles would be really, really great. It's ironic just before my team manager sent the email around from you, I was talking to the girls at work about at about just how saddle sores are like for cyclists and how rubbish it is. And they're like oh, I don't know how, like we haven't accessed a market here, we could create something. And I'm like, yeah, I don't know what though.. Yeah, definitely something female specific for saddles or how to prevent that. And then again, yeah, research into female specific training. I know that it's only really been recent and there's a limited amount of research in it, because there's been so little time to be doing it, I guess so. Yeah. Yeah, just everything. And just generally, females and research would be great, you know.

0:25:57.740 --> 0:26:4.750
*Interviewer*
Yeah, definitely. Have you got anything else you'd like to add before I turn off the recording?

0:26:6.300 --> 0:26:12.50
*P8*
I don't think there's anything else I can think that's relevant.

0:26:14.410 --> 0:26:16.450

*Interviewer*
No, that's fine. Right. I'll just stop the recording.

**P9 – interview transcript**

0:0:0.0 --> 0:0:9.340
*Interviewer*
OK, so to start with a bit of background about you and your cycling, what cycling events do you do and have you always been a cyclist?

0:0:12.90 --> 0:0:47.730
*P9*
I don't do any events now. Started cycling I think in 2006 when I took a triathlon but didn't start bike racing until 2015 when I did hill climbs, and then the following year in 2016 did some hilly time trials and hill climbs and then moved into general time trials and hill climbs and then couple of long ones - 100 mile and the 12-hour time trial. But I haven't raced since 2022.

0:0:49.510 --> 0:0:55.780
*Interviewer*
So in terms of your cycling at the minute you not competing, have you got any goals or is it just generally to maintain fitness?

0:0:57.480 --> 0:1:32.450
*P9*
Generally, just to maintain fitness… so bike touring in the summer like this weekend we'll go away and do a couple of long days on a mini bike tour. And cycling to work and back, which is about 2 hours each way, which I don't do every day obviously and not in the winter. So yes, I don't know at some point I don't know if I'd ever do a competition again, but the minute it's more just to keep fit and to be fit for long summer bike tours.

0:1:34.650 --> 0:1:42.450
*Interviewer*
So moving on to the menstrual cycle, thinking back to when you started getting periods. What was it like for you? How old were you at the time?

0:1:44.370 --> 0:2:1.20
*P9*
I think 13 or 14, and didn't have any particular issues. I think fairly standard at that age. I think sort of like one a month, pretty regular.

0:2:3.90 --> 0:2:5.770
*Interviewer*
Were you playing sport back then. Did it have any effect?

0:2:7.50 --> 0:2:14.830
*P9*
I used to be a runner when I was at school, and I don't know. I used to be quite fast up until about year nine and then dropped off markedly into year 10 after my periods had started and then didn't sort of get decent again until I went to university.

0:2:31.190 --> 0:2:40.470
*Interviewer*
When you started your periods, and did you get any advice or information at the time from school, parents?

0:2:44.20 --> 0:3:2.430
*P9*
Probably there was generally one annual school lesson I think, but I don't. There wasn't a great deal and my mum passed on a bit of information. Just the practical stuff, but nothing about really symptom or anything else.

0:3:4.730 --> 0:3:15.170
*Interviewer*
Then as you've got older, has your mental cycle stayed relatively similar or is it changed? I think you mentioned on your form you had been on a contraceptive pill for a while.

0:3:16.750 --> 0:4:11.470
*P9*
Yes. So, I went on acne medication called Roaccutane. I think when I was 18 and on that, I think it's a requirement that you go on the contraceptive pill because it's very dangerous to become pregnant if you are on that. And then when I came off Roaccutane and stopped the pill. I think I can't quite remember all the order, but I didn't have a period for maybe a year or something after that. And then since then, I've been on the mini pill, which didn't agree with me, so, I came off, but it's been sort of up and down, but I think my periods now a couple of years ago started to get heavier and then sort of every three weeks instead of every four but now they're back to being about every four.

0:4:13.990 --> 0:4:18.350
*Interviewer*
And you said the mini pill didn't agree with you. What sort of symptoms do you get on the mini pill?

0:4:20.540 --> 0:4:40.130
*P9*
Nothing in particular. I just felt like it messed up my cycle. I never really knew when I was going to get a period which I didn't like. So ultimately, I just thought it was easier to not be on it.

0:4:42.20 --> 0:4:54.860
*Interviewer*
In terms have now you said your periods are more back to being more regular, in terms of symptoms that you get what would you say, do you experience different symptoms throughout cycle or fairly symptom free?

0:4:56.400 --> 0:5:59.220
*P9*
A couple of nights before it starts, I generally don't really sleep and then have sort of abdominal pains in the night and the next day. So, then I know that it's sort of a couple of days away from starting. And then during the period, I think I am quite lucky in terms of symptoms like I have the abdominal pain at the start and then not much else, really. I think performance wise it's difficult to know when I did the monthly tracking survey that coincided quite inconveniently with my walking pneumonia. So, it wasn't very easy to see what the symptoms were, because I was coughing all the time anyway but I think in the run up to my period, I don't, like, the weekend before I am definitely not as fast or as powerful as I am normally for the first couple of days.

0:6:0.750 --> 0:6:7.90
*Interviewer*
So would you say your symptom burden or symptoms are worse? The kind of few days before your period actually starts?

0:6:8.420 --> 0:6:30.550
*P9*
Yeah. I think for me it's the few days before and then once I'm into it, symptoms, like the first couple of days are really heavy, but not. And I guess I wouldn't, I don't really know how I go on those couple of days. But then after that it's I think I start to get my performance back a bit.

0:6:32.120 --> 0:6:36.190
*Interviewer*
And you mentioned you get stomach cramps. Do you ever take any pain medication for those?

0:6:36.940 --> 0:6:40.430
*P9*
No, mine aren’t bad enough to need to take pain medication.

0:6:41.960 --> 0:6:50.950
*Interviewer*
You mentioned the first couple of days you get quite heavy bleeding. Does that have any impact on going out on long rides or planning toilet stops or anything to do with that?

0:6:51.850 --> 0:7:31.490
*P9*
Yeah. So, I never want to do a group ride or anything in the first couple of days and it is, so when we're touring that's always a problem as well when you're out all day and you have to factor in where there might be a toilet, so it is a deterrent. But equally, because we get so little good weather if it was on a day when I was going to go out, I think I would go out anyway it's just a massive hassle.

0:7:23.500 --> 0:7:28.100
*Interviewer*
Have you ever used any products like a menstrual cup to help with heavy bleeding and long rides?

0:7:29.450 --> 0:7:31.490
*P9*
No, never actually considered that.

0:7:32.670 --> 0:7:35.480
*Interviewer*
So is it just kind of Tampax and get on with it?

0:7:36.490 --> 0:7:36.710
*P9*
Yeah.

0:7:38.370 --> 0:7:46.170
*Interviewer*
In terms of we talked about kind of physical symptoms, you get any mood related changes throughout the cycle or is it mainly just physical?

0:7:47.550 --> 0:8:3.530
*P9*
I'd like to say no, but I think I am a bit more irritable beforehand and possibly more emotional in the few days before and the first couple of days after it starts, I think.

0:8:5.790 --> 0:8:18.990
*Interviewer*
And obviously you mentioned you would still go out in a long ride on the first few days of your period but in terms of training, I know you're not competing now but would you ever modify your training around those few days before your period starts in the first couple of days of your period?

0:8:20.540 --> 0:8:57.180
*P9*
I never used to moderate it because otherwise it felt like it was cutting out quite a lot of your training time, but now that I'm not competing and I don't feel that pressure to be as fast, I will modify things a little bit if, like I say, if it's going to be a long ride, I'd still do that. But apart from that, if I'm not doing a long ride, I can modify it a bit now.

0:8:58.580 --> 0:9:2.990
*Interviewer*
So in terms of like if you're on the turbo or whatever, you can maybe just do less reps or less intensity?

0:9:4.320 --> 0:9:26.690
*P9*
Yeah, like I wouldn't think now I'll go out and do hill laps I’d do that time when? Or like swimming, I'd go swimming and just do 100 lengths. Sort of steady. Rather than trying to do anything shorter and faster, I suppose I just don't really want to do like top end stuff beforehand.

0:9:29.420 --> 0:9:39.880
*Interviewer*
It's quite specific question do you ever experience any changes in you kind of balance, core strength, coordination, mobility throughout the menstrual cycle or have you never noticed that?

0:9:41.90 --> 0:9:57.400
*P9*
Never noticed, but, because I do Pilates 5 times a week and I normally do some balance sometimes I don't balance as well, but I've never actually thought about where that is in my cycle, if it could be linked to that.

0:10:0.210 --> 0:10:11.140
*Interviewer*
Also, you're not competing now, but when you were competing was there anything you would do differently if you were kind of around that few days before, on your period or would you just kind of get on with it?

0:10:13.350 --> 0:11:9.390
*P9*
I don't know really. It's not. Because it's really difficult when you're competing and if it's going to fall a race is going to fall kind of at the wrong time. So, I think I was quite lucky when I won the National Hill climb that was the day before my period started, so that was really the worst possible time and actually when I trained on the hill the weekend before I put out higher power than I did on the day of the race. But you can't ask a national event to move because of your cycle. So, I don't know really. I don't know what options there are or what there is that can sort of alleviate symptoms. I know some people take pills to not have a period coinciding with something like the Olympics or whatever, but it's pros and cons to that, so I, but I don't really know what I would have done differently.

0:11:10.740 --> 0:11:25.590
*Interviewer*
In terms of you mentioned that you sometimes have some nights disturbed sleep before you period starts would you do anything to if you had a competition to accommodate that, as in go to bed earlier to try and get more sleep that way to counteract the effect of that. Or would you just go normal bedtimes?

0:11:26.330 --> 0:12:10.90
*P9*
I think in the run up to a competition I would always try and get decent sleep for the whole week beforehand because if you've only had one night of bad sleep, that's not actually too problematic so if I've gone into it having had a few nights of good sleep then I'm not so worried. Also knowing why it's being caused I don't like, get worried when I'm in bed I think at least I'm lying here and I'm relaxing. I'm not, instead of getting worked up about it, I think when my eyes are closed, and I'll just lie here still and rest. I don’t get up because then I think well, may as well just lie in bed.

0:12:11.920 --> 0:12:22.0
*Interviewer*
When you were competing, would you talk about if you're going to get your period with a coach or other team members or anybody like that if you had symptoms?

0:12:23.110 --> 0:12:49.530
*P9*
No, my, the coach I had when I when I won the national hill climb, he would check in if I was getting regular periods, but we never talked about when they were or adapting anything. I think with hindsight, possibly should have done and factored in an easier few days or something once a month. But no, never discussed it.

0:12:50.760 --> 0:12:57.60
*Interviewer*
Have you ever had any advice around kind of the menstrual cycle and training and competing in sport?

0:12:58.640 --> 0:13:16.410
*P9*
No, I've read some things, but I've never been given advice, so I think there's a little bit more out there now and there's some stuff that suggests you are more liable to injury or ligament damage in the run up to period and things, but I've never been given any advice.

0:13:18.290 --> 0:13:31.830
*Interviewer*
Obviously you said you've read some stuff, are you aware of elite athletes talking in the media about the effect of periods on their performance so talking the likes of Dina Asher-Smith, Eilish McColgan, and has that had any influence on you?

0:13:33.340 --> 0:13:53.110
*P9*
Yeah. So, I've read. Dina Asher-Smith has been good at talking about things. Some of the British cyclists have mentioned it. And yes, Eilish McColgan, I think she said she felt like she ran like a walrus. Was that Eilish McColgan?

0:13:54.240 --> 0:13:54.880
*Interviewer*
I think so.

0:13:54.410 --> 0:14:9.310
*P9*
Which is not good for her obviously, but I think it is better that people are more open about those things now because historically I think when we first started competing, nobody spoke about it at all.

0:14:12.330 --> 0:14:19.780
*Interviewer*
OK, that is all my questions around periods and menstrual cycle. Is there anything else you think I've missed out or should have asked you or you want to say?

0:14:21.410 --> 0:14:49.480
*P9*
I don't think so. I mean, I do it because I when I was doing the monthly tracking that would have been quite interesting to know. if I wasn't coughing all the time because there is a period of the month, I think we're, I'm definitely most energetic. But I'm not 100% sure when that is. I suspect it's probably somewhere around the middle where you feel like you just got a lot more energy, but.

0:14:49.610 --> 0:14:56.910
*Interviewer*
I suppose based on that would you consider doing tracking in the future if you were looking to train properly for something?

0:14:58.910 --> 0:15:28.880
*P9*
Yeah, I think so and I wouldn't rule out, even though tracking every day feels like a bit of a burden. But I think just keeping a loose tab of where you are when you're cycle and then like when I've had a really good swim session or I have had really good legs when I've been cycling probably is quite useful to know. So, you know when you can really go for a hard session or when you should be dialling it back a little bit.

0:15:31.970 --> 0:15:48.930
*Interviewer*
So on to the next part of the interview on kind of bike equipment and injuries. I think when you filled out the questionnaire in terms of your bikes, you ride a mix, I think maybe only one of them is women specific. Is that right? You're light tourer?

0:15:50.420 --> 0:15:53.700
*P9*
Yes, I think so, yes.

0:15:54.440 --> 0:16:7.840
*Interviewer*
So for any of your bikes I suppose mainly talking about the ones you've raced on or do your kind of main summer training have you made any modifications to those to suit you? So, kind of stem length, handlebars, cranks?

0:16:9.870 --> 0:16:43.190
*P9*
Yes, but I would struggle to say exactly what all of them are. All of them have narrower bars to what they would come with. And stem length, I think we've put a slightly shorter stem on one of my road bikes. I think cranks I just ride what they've all come with, so it's mainly just the handlebars, I think.

0:16:44.60 --> 0:16:47.370
*Interviewer*
Have you done anything to change the brake lever position?

0:16:48.320 --> 0:17:14.480
*P9*
Oh yeah, because I've got tiny hands, so I've had to change the brake levers on quite a few of them actually. I think I'm one of my commuting bikes the first one I got was a was a 51 centimetre one but when I bought the second one they were just small, medium or large. So, I bought medium, with hindsight, I think it was a mistake so I put a shorter stem on that one. But yes, to be able to reach the brakes, we've had to put those in.

0:17:16.570 --> 0:17:25.910
*Interviewer*
Have you ever suffered from any cycling overuse injuries? So lower back pain, knee pain, saddle sores, foot pain or hand/finger numbness?

0:17:28.500 --> 0:17:50.520
*P9*
Nothing long term in terms of hand finger numbness, like sometimes on the ride I do, but nothing lasting. The only lasting thing is saddle issues. I think I've been quite lucky unless I'm forgetting about something, I don't think I've had anything else long term with cycling like back pain and stuff.

0:17:52.350 --> 0:18:0.970
*Interviewer*
In terms of you mentioned you have saddle issues in terms of the saddles you ride at the moment, have you changed those to try and help with any saddle issues?

0:18:2.300 --> 0:18:50.60
*P9*
So I hate everything to do with saddles because I never know if the new ones going to be better. So, at the minute on my summer road bike, I've been thinking about changing it to for quite a long time, but then I think is it bad enough to change or now that are not cycling as much is it worth risking something that worse. I think ultimately, I probably should have one comfortable saddle and put the same one on each bike which my husband has done. He's got one that works and he's got the same one on every bike, but I'm never convinced that I've got exactly the perfect saddle.

0:18:51.590 --> 0:18:55.850
*Interviewer*
And when you used to time trial, did you ridea different saddle on a TT bike?

0:18:57.980 --> 0:19:43.870
*P9*
Yeah, so the first saddle I had in my TT bike was absolutely excruciating so, somebody gave me one of their rejects that was a bit more comfy. I think with hindsight, it wasn't really that comfy. I think it's really difficult to know because there was that phase of having ones with the cut out in the middle, but then they just put a load of pressure down the outside and that was where my issue was down the outside, so I didn't ever want to go to a cut out one in case it was just going to exacerbate the issue. But I know there's been a lot of female saddle developments in the last few years, and I do think I should probably put more effort into it. It's just that worry about spending hundreds of pounds on a saddle and then it being less comfortable.

0:19:45.430 --> 0:19:51.650
*Interviewer*
So in terms of your saddle issues, you said it was on the outside. This was it upper thigh, outer labia that was the problem?

0:19:52.680 --> 0:20:18.570
*P9*
Outer labia just on one side, so I don't know, but I don't know what. Why it was on one side if I was like riding at a funny angle or if, somebody did say to me, a man unsolicited at a race that said a lot of women have a labia imbalance, which is interesting to know, but also really odd thing to say to a woman after a 10-mile time trial when you've never met her before.

0:20:20.880 --> 0:20:29.240
*Interviewer*
In terms of like getting saddle soreness was there anything that made it worse so types of training or racing or position?

0:20:30.930 --> 0:21:31.70
*P9*
Well, I think the turbo is a terrible thing to do for saddle sores because you are just in one position, and I think with hindsight that's when I first started turboing in like January 2018. When I start doing a lot and it came on like moderately quickly but then it was also winter so, I was turboing more than you would ordinarily. So I went to the doctors then and she said women don't get saddle issues, but men do. So, she said it, because I said my concern is, is it doing lasting damage? And she said no. Turns out she was actually wrong, and it has done lasting damage, but she was a doctor, so at the time I believed her. So, I think it was sort of it come from turboing and then probably I mean time trialling’s not a great position to be in because you're not getting out the saddle at all or anything. So, I think it's probably just a combination.

0:21:33.200 --> 0:21:38.20
*Interviewer*
And obviously you said it's done lasting damage as it just kind of swollen on one side is that?

0:21:38.790 --> 0:23:9.220
*P9*
Yeah. So, I think the doctor said when I went was it last year or two years ago, she said at the tissue has hypertrophied where it's like was gone into that position and won't go back. So, I've been to see a gynaecologist about it, but she said the options, well, she said they don't do anything for cosmetic reasons, which was not why I was going but she said, although they could do surgery she thought it could potentially be worse with the scar then it would be from having the swollen tissue, so I've just left it as is and I guess I'm not cycling as much anymore. I wouldn't rule out going back in the future cause I've spoken to Jasmine Muller, who had won the world 24-hour time trial who was very public about her saddle issues. So I got in contact with her, and she was bounced between loads of different gynaecologists and people and then eventually, somebody said she should have surgery. And she said she never looked back since the surgery. But I don't think mine at the minute is severe enough for surgery and I do think the gynaecologist has a point about the scar being potentially more problematic. That is obviously a concern depending on who does it and how they do it.

0:23:11.80 --> 0:23:18.430
*Interviewer*
In terms of now, does it cause you a problem when you ride? Is it uncomfortable or is it kind of manageable or have you just got used to it?

0:23:19.640 --> 0:23:48.400
*P9*
It is still uncomfortable, so I can feel it on every ride, but I think I get used to it because it's not a really sharp pain. It is just a feeling of being bruised and I was worried two years ago when we cycled to Amsterdam to Berlin that it would be really sore. But I suppose on that because we were touring, we were stopping every couple of hours and it was actually, I could feel it every day, but it wasn't excruciating.

0:23:50.170 --> 0:24:1.780
*Interviewer*
Also you've mentioned you sort some medical advice around this. Have you done anything else to help with it? Like a bike fit, looked at imbalances if it's a one side or anything like that, change shorts?

0:24:2.980 --> 0:24:50.380
*P9*
No. So, I should do all of those things, so. There was a saddle, I think Specialized released a new saddle that a few people raved about for women and I was going to look into that, but then it was more than 100 pounds. I sort of put it off, but I think realistically I should do more to make sure I'm not sort of loading stuff unnecessarily. I don't quite know what you do when it's one side rather than the other. I don't know what the options are, but I think a proper bike fit probably would help. It's just that worry of getting somebody who actually knows what they're talking about and understands like the women's issues.

0:24:52.540 --> 0:25:1.10
*Interviewer*
And would you say, obviously, it sounds like it does if you're uncomfortable, does it affect your enjoyment of cycling? Does it mean you maybe do less than you used to or?

0:25:2.950 --> 0:25:44.350
*P9*
Yeah. So, I think there's lots of things I've got used to now is when I'm approaching any kind of a rough road surface, which is of course 90% of Britain's roads, I get out the saddle, so we even like rough tarmac is uncomfortable to rattle over which is a lot of tarmac, so I could at the saddle quite a lot. I don't. I think if I was still racing and still doing the amount of cycling I used to when I was going six days a week, I'd definitely have to take it more seriously. Just at the minute, I mean, over the winter I hardly cycled at all. Over the summer when it goes back up again I do think I need to approach it with a more scientific approach than my current approach, which is just hoping it recovers enough in between rides.

0:25:46.90 --> 0:25:53.0
*Interviewer*
Obviously you mentioned it started when you did a lot of turbo, and when you're training hard, did you ever discuss it with your coach at the time?

0:25:54.970 --> 0:26:0.830
*P9*
No, which with hindsight obviously was a mistake, but I just thought I just need to get faster.

0:26:2.970 --> 0:26:6.490
*Interviewer*
Did you take any time off the bike, or do you miss a training session because of it?

0:26:7.220 --> 0:26:9.710
*P9*
No. Again, possibly a mistake.

0:26:13.60 --> 0:26:18.320
*Interviewer*
That's all my questions on saddle sores and bike fit is anything else you think I should have asked you about?

0:26:19.990 --> 0:26:23.440
*P9*
I don't think so, no.

0:26:25.20 --> 0:26:38.370
*Interviewer*
And then my final question, are there any areas you would like researching, in regard to female cycling? So menstrual cycle, training for females, bicycle setup, saddle choice, injuries, for example.

0:26:39.410 --> 0:27:52.960
*P9*
Yeah, basically all of those. I think it would be really useful to know what performances like at different stages of the menstrual cycle and if you are more likely to be injured in the run up to your period starting, so I think was it just the other week, was it the hockey team? Women's hockey team were talking about they thought they were more prone to injury in the run up to their cycle. Obviously in cycling you're not doing the same type of movements you are doing in hockey where you're much more likely to tear ligaments or something, but I think it probably would be quite useful to know. And also saddles because I think historically everything's been done about men and I think obviously the cut out in the middle of a saddle can be great for men because that alleviates their pressure point but doing that for women can be worse. Yeah, I think saddle and how you should train at different parts of your cycle would probably be quite useful. And also just to help calibrate your expectation of knowing, oh, it's like a few days before my cycle starts I might only reach like 90% of my power or something.

0:27:55.620 --> 0:28:1.630
*Interviewer*
OK, great. That's all my questions. Is there anything else you'd like to add before I stop the recording?

0:28:3.480 --> 0:28:5.100
*P9*
No, I don't think so. Thank you.

**P10 – interview transcript***Interviewer* 0:29
So when did you start cycling and how long have you been competing? So just start with some general questions.

*P10* 0:38
So I've always, always ridden my bike, but I started competing at pretty late really, relatively, it was about I think 2016 when I started competing, I stopped because I was running before that. So yeah I was competing in athletics and running but it was cycling in 2016 when I made this switch. So, eight years, yeah….

*Interviewer* 3:46
So going on to the menstrual cycle. How old were you when you got your first period and what was that like for you?

*P10* 3:55
I was 13. I didn't really want it. I definitely know I definitely didn't want it, but it was fine. I got the hang of it pretty easily. My mum was always talking to us about it and we're quite open about it with friends and stuff, so I didn't want it, but it was fine.

*Interviewer* 4:10
Where you doing sport at that time? Did that have any effect on it?

*P10* 4:21
I was doing sport at that time. I don't remember being affected from the perspective of like never have, I got like stomach ache and stuff like cramps and things like that, but I don't remember ever being like, like, really physically affected as a teenager or sort of held back, particularly by it. So, I think it was, you know, a bit of a nuisance to have, but I don't think I was really troubled by it.

*Interviewer* 4:49
Because were you running at that point? Was that your sport when you're a teenager?

*P10* 4:55
Yeah, it's doing lots of sport, lots and lots of athletics, so I don't remember it being really stand out problem put it that way.

*Interviewer* 5:05
Did you get any kind of information or advice around that time you mentioned your mum, but did you get any others from school about periods?

*P10* 5:15
School, definitely yes I think, and then once I got a bit older, when I was training around the age of like 17-18, then my coach, he would try and talk to us about it as well. So yeah, I feel like it's always been kind of a discussed topic. I don't think that there's ever been a complete that we are even now, like, not such a necessarily looking, only recently such an understanding of how different points in the cycle affect how you feel and they're definitely wasn't that, it was more just like, you know, if you're on your period and you're training and you don't feel good or something, then that's understandable. It was a bit like that.

*Interviewer* 5:55
Obviously you mentioned like you've had some changes as you've got older like how was your menstrual cycle changed? You've also, I think, mentioned to being on the pill for a bit.

*P10* 6:07
Yes. And when I was about maybe about 18, I went on to a contraceptive pill and then sort of in my, it was just quite light, but it was normal, consistent and stuff. And then in my sort of mid 20s, it just kind of was getting lighter and lighter and then dwindled to nothing. I think I actually I was, I got ill, I had some, I can't remember what was wrong with me, but after that I got really ill and I lost, you know, some kilos in a really short amount of time and then after that it never really like established itself back again. And then I got really into cycling and then that was it, like, really inconsistent and I've always been pretty slim and doing lots of sport. I think when I got into my cycling it was like I suppose it's just those couple of kilos lighter that makes a difference. When I would have like off season and I would take some time off my bike, that's when I'd get period or if I was injured and had some weeks off then I'd have one. So, it was definitely, well, I think it was a case of sort of like energy, energy, like lack of energy availability really. Because then yeah, it was like, yeah, really in consistent, like one or two a year and then when I started to think about having a baby, I'd had a period in the April and then in the summer I was thinking right. Well, if I want to try and get pregnant at some point, I need to try and get a regular period for at least six months beforehand. So, then I, I didn't stop training, but I ate loads more and made an effort to eat like 80-90 grams of carbs on a training ride even if I was just sort of tapping around slowly, they literally put on like 1.5 kilos and then I had one period and then I got pregnant, so.

*Interviewer* 7:53
So as you say probably just that very small, about 1.5 kilos, 2 kilos, that seem for you anyway must make the difference.

*P10* 8:00
Yeah, I think so, yeah.

*Interviewer* 8:02
When you did get your period, obviously it was very infrequent. Did that ever cause you any problems around training or race? And if it will probably didn't come during race season, but did it ever cause you any problems? Any symptoms, cramps or?

*P10* 8:16
Oh yeah, so I take paracetamol because I'd have like that dull ache, like lower abdomen ache. But it's never been like so bad that I can't do anything, and definitely I remember mood swings like I used to have mood swings, of course. But again, it wasn't like really debilitating. I think if I had them regularly then I would definitely notice changes throughout the month because I'm quite in tune to how I feel, but I just didn't have them regularly enough.

*Interviewer* 8:38
Yeah, not getting regularly periods did that give you any other symptoms of like relative energy deficiency or apart from not getting period? Were you healthy?

*P10* 8:55
So everything else seemed like it was absolutely fine. Like, I always felt good, but my DEXA scans showed slightly dropping numbers, which is something that I'm looking at like yeah, more now, I think pregnancy helped recover it a little bit, but it was it was showing a sort of like a downward trend in bone density, which obviously doesn't help, cycling doesn't help, but also lack of periods or energy availability doesn't help either.

*Interviewer* 9:24
Yes, was that something raised by a doctor that you needed to get that checked out, or was that just something you thought you may be needed to get looked at?

*P10* 9:30
Yes, through our team, they just monitor it. So, they send us for regular bone density scans.

*Interviewer* 9:35
OK. In terms of like with the team, if you had any conversations around your periods or it not being regular or anything like that?

*P10* 9:44
Yes. So even if it wasn't like I was trying to get thinking about pregnant, getting pregnant, we, that was the like a goal was to try and have a regular period because we it's just it's good for performance. You will perform better as an athlete.

*Interviewer* 10:03
And in terms of like not maybe you, but in terms of your team, do people openly talk about menstrual cycle, periods if they're not feeling great, if it's race day?

*P10* 10:16
Yeah, some people will, but I think that there's probably a lot of, a lot of the team that don't have regular periods actually, because the amount of people, times, I hear someone say oh, I've got period pains or something it's really infrequent like, yeah, it happens. Somebody might say it, but it's not very often.

*Interviewer* 10:33
In terms of around the menstrual cycle you mentioned obviously, when you were a teenager you were running, it was more just like, are you getting your period do you feel a bit rubbish? Has I suppose, some other elite athletes the likes of Dina Asher-Smith, Eilish McColgan talking about it has that made you more aware of it? And I suppose the different phases and cycle and kind of the importance.

*P10* 10:55
They feel certainly in the last like 10-15 years. It's been much more topical than when I was like 15. I think it's become. Yeah. And there's so much more like understanding now or encouragement actually to track it, to understand when you feel good and why you feel good at different points in the cycle and understand like what your hormones are doing and when it's a good time to train in a certain way or why you might not feel good. And so, yeah, I think there's been a huge increase in the understanding and the encouragement for athletes to try and like, not necessarily use it to our advantage, but to prevent it from being a disadvantage.

*Interviewer* 11:31
Do you talk to your coach about your menstrual cycle, or anything related to that?

*P10* 11:38
I would do there’s not a lot to say.

*Interviewer* 11:40
Yeah, because I think is it right, you have got a male coach. So, have they ever asked about it? Or is it just not kind of come up, or was it up to you?

*P10* 11:48
So he, yeah, I suppose it was. It did come up when we were talking about how it needs to be something that we get, that I wanted to get back but yeah, I don't. I would do if there was more to say.

*Interviewer* 12:05
Have you had anything, obviously you've completed for GB. Have you had anything from British Cycling, any education around it? I suppose you on the periphery.

*P10* 12:16
No I think the girls that are on the track who are much more under a GB program, absolutely have a lot of focus on it, but because on the road you go and compete because you're much more assigned to your team. So, it's much more about like support on the day of the racing opposed to like full round the clock support. But I know that they do give it and it is it is topical.

*Interviewer* 12:41
That's all my questions around the menstrual cycle. Do you have anything else you'd like to add or?

*P10* 12:51
Nope, I think that's what. Yeah. Think I've said everything.

*Interviewer* 12:54
Ah, sorry, one question I realise from for me. Question one reason you don't have to answer this, but was there a reason you came off the pill? Was that just you wanted to go on to a natural cycle and get normal hormones or?

*P10* 13:05
Yeah, I think so. I've been in a long-term relationship for yeah, like five years or so and then that ended. So, I was like oh, just come off it and see whether I can get back a normal like could get that having period or something again. And then, because I wasn't really having one, I was like, well, I don't really need to be on it so. Well, that's suppose. Yeah, that's the other thing. So then then I had. Then I got into a new relationship with who's now my husband, and we basically essentially had unprotected sex for like 4 years and then it was like oh, I just kind of put on a kilo and half wanted to get pregnant and had one period and then got pregnant. It's all quite surprising. I wouldn't encourage them method to most people.

*Interviewer* 13:50
But it worked out for you.

*P10* 13:51
It worked out for us yeah.

*Interviewer* 13:51
OK, one second, so the next part of the interview is on kind of bike set up and obviously you ride for teams. So, you've got team issue bikes that are not kind of women's specific. Do you make any modifications to those to suit your position in terms of things like handlebars, crank length, saddle, etc?

*P10* 14:14
Yes, so we've got the bike fitter that comes specifically to get us set up. So that's, yeah, interesting because handlebars we can go narrower, but to be honest, the men all want to go narrower anyway than what is actually standard to what you can even get. So, we are on the most narrow that we can possibly get which the men want anyway. Crank length the same actually if you want them a bit shorter, I mean, obviously that's kind of like height dependent anyway, but now everybody seems to want to go shorter because shorter is more aero, so a lot of the things that you might deem to be women specific are kind of almost like performance specific, like there almost there anyway. So, saddles has been a really big topic each year with the team they're really, really looking to find the best options for the team because we've had really big problems in the first year of the team in 2022 with saddle sores, and we've had riders have to drop out of stage racing and stuff because the saddle sores have been so bad. So, whilst we have well, we have a saddle sponsor, its still very free reign, like you can try their saddles. We've got a range of different ones, different widths, different shapes. They look at your seat bones, look at your position on the bike, look at what saddle could be good, but if you need to have your own saddle then you have your own saddle.

*Interviewer* 15:41
Do you ride your own saddle, or do you ride a team sponsor one?

*P10* 15:46
I have a team saddle. I do have some saddle sores like I do get them paerticularly when you're just doing lots of hours, but it's kind of that slight middle ground where it's never really that bad to bother doing anything about it.

*Interviewer* 16:02
Yeah, and it's it, you mentioned time on bike is an issue. Is it a bike? Is it worse in time trial position or in terms of?

*P10* 16:11
So when on my TT bikes. I do all my training on the turbo on the TT bike and then I get them there. So, I've got the narrowest ISM that you can get, but I still will tie the front nose, which is not recommended, but it makes it better for me. Quite a lot of them I think that it's not necessarily just the saddle that's a problem or just your bib shorts. It's usually a combination of the two together where the seam sits in relation to the saddle, and I have had problems before, and we did some like pressure mapping and then trying to adjust the position of how to how I sit. But yeah, definitely, yeah, both saddles for long duration I get little sores and like sort of the top of like the like top of the leg like underneath the bum cheek almost on the inside.

*Interviewer* 17:03
OK. Do you get any problems more further forward, so kind of upper thigh, kind of outer labia, anywhere like that? Or is it mainly round sit bones?

*P10* 17:11
No. Yeah, sort of. Like, yeah, it's almost like whether the seam is from the edge of the chammie.
So like the bum crease, that's where I tend to get them.

*Interviewer* 17:22
Is it more like a spot, or is it kind of chafing, pressure related?

*P10* 17:25
No. So, but yeah, like, a lump.

*Interviewer* 17.2

OK so has that ever caused you to have to take time off the bike?

*P10* 17.28

Yeah, sometimes it's like after. So, we had a really wet stage race this was the one where some girls actually drop out of the race, really wet stage race, and that made it much worse and then after that, like I had to have some days off. But generally what happens like in with training I'll do an endurance say I'm doing an endurance block and they'll be getting worse over the days you get on the bike and you're like oh, that's a bit uncomfortable, but you get going and it's fine, but then you have a rest day and then in the rest day it actually recovers quite a lot. And then by the time you get back on your bike, the next time it's kind of fine again. So, it's generally it's not too much of a problem, but I do have saddle sores, if that makes sense.

*Interviewer* 18:15
Yeah, if you have had to get medical treatment for a saddle sore or you just kind of managed it with time off the bike?

*P10* 18:22
Just managed it with time off the bike, yeah.

*Interviewer* 18:24
In terms of like your saddle, you said you'd had some pressure mapping and use a team saddle, have you ever done anything like, have you got tilt on your saddle because they're obviously a lot now females put nose slightly down for TT. Have you ever done anything like that to kind of help?

*P10* 18:41
I'm very, very, very particular about the angle of my saddle on my TT bike if anyone touches my saddle I sit there with my spirit level like it has to be exact, but actually it's just completely flat. I find that when you tilt the saddle really small amount up or down it has a massive impact for me where I can deliver power from whether it's like from above my knees or whether it's coming from my glutes or whatever. So, I'm, I would say much more obsessed with the angle of my saddle because of power production and not because of saddle sores.

*Interviewer* 19:07
In terms of overuse injuries, have you had any other overuse injuries like lower back pain, kind of foot pain, hand numbness, knee pain?

*P10* 19:24
So I was having bad problems with getting really numb hands on my road bike but then that was one of the things I looked at with the bike fitter changed my position a little bit, changed the position of the cleats as sometimes I get numb feet. Then I get really bad pain in my lower back on the right side, but only when I'm in Andorra, because there we climb, you climb for like hours and hours and so it's really high torque, pretty low cadence like I've got you get like we'll see more gears for it, but you still spend a lot of time at like 70-75 RPM and the sort of, and that strains my lower back.

*Interviewer* 20:04
Yeah. So, has the bike fitting helped on any of those or is it just something you put up with when you are in Andorra that you're going to get back pain?

*P10* 20:12
I still get a little bit, but it's not like it was. It's much better.

*Interviewer* 20:16
OK. And in terms of discussing it with your coach, do you discuss like things like saddle sores with your coach, if you have to have time off the bike or do you ever modify a session to accommodate a saddle sore? Maybe reduce the length or anything like that?

*P10* 20:31
Yeah. So, before I cable tied the nose of my TT bike, I was getting pretty bad saddle sores on the long endurance because it's when, I don't, it's only when the weather is really bad and we were in Andorra that I need to do endurance on my TT bike inside. Otherwise, I would ride out. And that was happening. And so, it was like, yeah, OK. So, we just had to make the sessions shorter and then cable tied the nose. But yeah, then we'll tweak the session to be more comfortable. So yeah, no, I have told him before and after the stage racing. I'll say I'm really sore. I might need a day off because I need to let my saddle sores get better.

*Interviewer* 21:04
That's all my questions on kind of bike set up, saddle sores is there anything you would like to add in terms of injuries, saddle sores?

*P10* 21:17
It's funny because when I don't have them because right now, I've just had a week off the bike because I had a bit of a crash and I'm like oh no, it's no problem. But then when they're bad, it's like the most important thing in the world but then and then the day goes by and then they get a bit better and then I forget about it. So, I do, it probably is worth addressing, and it probably is bad enough. I think we just learned to live with it. Really think we do, don't you?

*Interviewer* 21:40
I would describe I ride the least worst option for my saddle like I've tried loads and I just feel I just can't seem to ever get fully comfortable and I just put up with it.

*P10* 21:47
Yeah, exactly. It's just, it's not that bad, but it could be better.

*Interviewer* 21:53
Yeah, like I don't need medical attention, so I kind of, yeah, I'm not on that level. So yeah.
Although maybe after the 100 I could have done with some like morphine, I think, but yeah.

*P10* 22:02
Oh my god, so uncomfortable. Actually, there was once I've had a different saddles sore, so I changed my saddle and this was back in 2022 and I was trying some different ones and the nose of the saddle basically pushed the chammie up like inside and then I got like a blister, literally like where like, I wee and that was like well painful. So, I quickly took that saddle off. So yeah, that was the only other time I've had another really bad one.

*Interviewer* 22:29
And then my final question, are there any areas like researching in regard to female cycling, so menstrual cycle, female specific training, bike setup, saddle choice, injuries?

*P10* 22:46
Particularly, I'd be really interested in more research around women and like fat metabolism and like fasted training and things like that, because I think there's really little research which is women specific, and women quite often will just follow advice and guidelines to men and their fast approach to fasted training and yeah, training. Yeah. And I think that that's really like I think it's pretty dangerous and I think it's misleading and I think that's one of the reasons potentially one of the reasons why so many athletes have problems with REDS, because training without any, without eating, I think is it has a different effect on our bodies to or more extreme effect potentially but there's just not very much female specific research.

*Interviewer* 23:26
Is there anything else you'd like to add which you think is worth looking at?

*P10* 23:33
What else would I be interested to read. I think probably around saddles actually like actual proper research on how women sit on bikes. I'd love to see the results for a whole host of different shapes of women with a whole range of different saddles and see what sort of shape roughly each woman rode each saddle, sort of what they felt like that would be interesting.

*Interviewer* 23:55
Yeah, I've got one of the women I race against the northeast that's what she really wants. Thinks would be the most valuable because people just assume women are all the same and we're not. And so, what works for one rider and really doesn't. And yeah, and so OK. That's great unless you've got anything else to add I will stop the recording.

*P10* 24:16
Yeah, that's fine.

**P11 – interview transcript**

0:0:0.0 --> 0:0:9.460
*Interviewer*
So just a bit of background about you and cycling and what, when did you start cycling and what events do you compete in?

0:0:10.70 --> 0:0:12.150
*P11*
So I started cycling in, it was 2011 that I started training and racing seriously. I did road racing for 10, well, 11 years. I stopped doing that in 2022, and since then I've been focusing on Zwift racing.

0:0:28.660 --> 0:0:31.0
*Interviewer*
How is your training and competitions going at the moment?

0:0:31.590 --> 0:0:54.870
*P11*
I think they're going well. I mean, I'm kind of in my offseason at the moment because the Zwift season is in the winter. So right now, I'm doing base training really and I haven't been racing that much recently. So yeah, I'm not in probably, not in top race shape, but you know.

0:0:57.390 --> 0:1:3.870
*Interviewer*
What are your targets for well, I suppose the season coming up in Zwift over next winter, have you got any targets?

0:1:4.590 --> 0:1:26.440
*P11*
Yeah, …. I'm hoping to qualify and compete at the world's in well I think it would be about six months time.

0:1:26.810 --> 0:1:31.850
*Interviewer*
Does that have qualification races then leading up to September? Do they have some earlier racing to account for that?

0:1:32.380 --> 0:1:48.320
*P11*
Yeah, they do. But then you can also get selected by your National Federation and so, I'm hoping because I did pretty well at the world's last year, I'm hoping that I will get selected by my this year.

0:1:50.440 --> 0:2:17.650
*Interviewer*
Great. So just, now starting on some questions about your menstrual cycle. Thinking back to when you start getting your periods and what was it like for you, how old were you at the time?

0:2:1.930 --> 0:2:31.270
*P11*
So I was eleven when I got my first period and at the start, they were quite irregular, which I think is quite common when you first get your period like I'd, I'd often like skip months, maybe have it like every other month but you know, nowadays they are more regular. So, my cycle tends to be between 28 and 35 days long.

0:2:32.420 --> 0:2:38.920
*Interviewer*
When your period started were you playing sport then? Did it affect you participating in sport at the time?

0:2:39.360 --> 0:2:46.440
*P11*
I was playing sport, but not at an elite level, so just doing sport for fun, really.

0:2:47.810 --> 0:2:56.610
*Interviewer*
Yeah, and did at that time did you get any advice or education from anybody - information around periods, I suppose from school, parents?

0:2:57.600 --> 0:3:22.480
*P11*
Yeah, so I mean my mom was very good because she told me all about it when I think it was ten, and she explained to me, you know, what a period was and like, you know, when I could expect to get my first one. I also had education at school from a school nurse who spoke to all the girls in my class about it.

0:3:24.310 --> 0:3:30.810
*Interviewer*
As you've got older, has your menstrual cycle remained relatively similar and regular? Or have you experience changes?

0:3:32.130 --> 0:3:57.120
*P11*
I have experience changes. So, I did go a year without having a period in 2014 and I think that was due to training hard and not eating enough to support the training but thankfully you know I managed to get it back and since then my period has been regular.

0:3:58.610 --> 0:4:2.980
*Interviewer*
What in terms of symptoms do you get throughout the month?

0:4:4.50 --> 0:4:49.160
*P11*
So normally about two weeks before my period, I get hunger pangs. So, I'm really hungry, can eat everything. Then about five to seven days before I start having PMS symptoms and sort of lose my appetite a bit and the symptoms can be that I'm feeling, you know, feeling dizzy, a bit lightheaded, sluggish, and struggle to sleep. It doesn't impact my day-to-day life. I'm still able to do everything, I go to work and ride my bike, but in terms of training and performance I do need to scale back on the intensity of training.

0:4:50.830 --> 0:4:57.290
*Interviewer*
I'm in that with those PMS symptoms do you get any mood related symptoms or is it just more kind of disturbed sleep and dizziness?

0:4:58.230 --> 0:5:11.770
*P11*
It's more I say it's more physical than mental. Sometimes I, sometimes I can be a bit more sensitive and cry a bit more but I think the symptoms are mainly physical.

0:5:13.320 --> 0:5:23.910
*Interviewer*
So in terms of you said, you need to maybe scale down training and performance, would you still race in that period? I know obviously we can’t dictate racing around our menstrual cycle.

0:5:24.0 --> 0:5:24.240
*P11*
Yeah.

0:5:24.100 --> 0:5:29.620
*Interviewer*
Would you still race, or would you just get on with it? Yeah, how would you adapt to that?

0:5:30.150 --> 0:6:1.190
*P11*
Yeah. So normally I wouldn't race in that week. So, I try to plan my racing in a way that I don't need to race in that week. I didn't used to do that. It's only the past year they've started doing it. Yeah, because like when, when I race, I like to feel like I'm, you know, 100% in good shape and yeah, and if I know they can't perform at my best I prefer to not race.

0:6:3.130 --> 0:6:9.180
*Interviewer*
In terms of performing at your best, is it mainly just that your power is not as good or do you have other factors that affect you?

0:6:9.730 --> 0:6:27.680
*P11*
Yeah, I think I think it's just that my power isn't as good, and I get tired quicker as well. So like, I tend to like, say in a one hour race I tend to like not have as much left at the end as I normally would.

0:6:29.100 --> 0:6:40.500
*Interviewer*
During like your actual period itself, so, when you’re bleeding do your symptoms ease or do you suffer any other different symptoms during I suppose the kind of first few days of getting your period?

0:6:41.420 --> 0:7:3.570
*P11*
So I get a little bit of cramping on the 1st and 2nd day but I used to have worse cramps, say like four or five years ago, they used to be a lot worse but now I found the medicines was called mefenamic acid, which I take the first two to three days of my period and I think that really helps to ease the pain.

0:7:5.820 --> 0:7:17.200
*Interviewer*
In terms of like being on your period does that affect how you train? Certainly, I know you focus mainly on Zwift racing now, but would that influence you're going out on the road or like in case you need a toilet stop or things like that?

0:7:18.10 --> 0:7:40.590
*P11*
Yeah, I think I, I mean I train mostly indoors anyway now, but even like when, when I when I was training a lot more outside, I prefer to stay indoors if I had that period symptoms, because then I knew that I'd be able to stop if I didn't feel well, whereas like it's more difficult to do that out on the road.

0:7:44.70 --> 0:7:56.710
*Interviewer*
Another question in terms of like throughout the menstrual cycle, do you ever suffer any kind of changes I suppose in balance, coordination, strength, so you mentioned a bit about power or flexibility throughout the menstrual cycle?

0:7:57.840 --> 0:8:23.320
*P11*
Yeah, I do feel because I do strength training as well and I think I lose a bit of strength during that week leading up to my period and sometimes I wouldn't say that I lose balance, but I lose focus, you know, I said that I get a bit lightheaded and dizzy, so sometimes I just lose focus on what I'm doing.

0:8:24.810 --> 0:8:31.20
*Interviewer*
In terms of if you say you don't feel as strong if you do strength training, do you modify your gym sessions in that week before your period?

0:8:31.510 --> 0:8:39.350
*P11*
Yes. So sometime to do lighter weights and fewer reps as well.

0:8:40.870 --> 0:8:52.390
*Interviewer*
In terms of all, obviously you mentioned you make a few changes to your programme around that week before you period. Do you discuss that with your coach? Do they, I think you said you had a male coach, do they accommodate that in your training program?

0:8:51.960 --> 0:9:25.420
*P11*
Yeah, so my coach is really good. I normally just tell him, like when I start feeling the PMS symptoms, I just tell him and I say that oh can we can we modify the sessions a bit and normally what he does is like he'll give me the same sessions with just lower the power targets and yeah and if that doesn't work he'll just you know put in sessions that are easier but he knows that I need to be more flexible in that week.

0:9:25.360 --> 0:9:30.990
*Interviewer*
Do you ever miss a days training or take more recovery in that week, or do you just see how you feel?

0:9:31.490 --> 0:9:43.810
*P11*
I just see how I feel. Sometimes I need to take extra rest if I'm really not feeling well which is it is not always the easiest thing to do, but you have to listen to your body don't you?

0:10:1.590 --> 0:10:4.750
*Interviewer*
Obviously we talked about you trying to avoid racing in that week before, would you do anything different, if you qualified for the worlds, you won’t get a chance to define when that is, would you do anything to help you prepare, if you did have to race during that week.

0:10:5.950 --> 0:10:36.640
*P11*
I think I'd probably like if it was a big race like the world's and I had PMS symptoms, I wouldn't miss it. But I think I, you know, I adjust my expectations and so, you know, I'd go into the race and just think, OK, well, instead of aiming for see a top 20, I'll just aim for, you know, maybe, say, say the top 40 or I could help a teammate instead. Or just try to adjust my goals based on how I feel.

0:10:38.80 --> 0:10:47.390
*Interviewer*
Do you speak to your teammates about periods or if you've got any symptoms? Obviously, you've mentioned that you let your coach know, do you speak to anybody else about them?

0:10:47.890 --> 0:11:18.190
*P11*
Yeah. No, I think my teammates are really good. So, we speak to each other quite often actually like you know if I have to miss a race, I'll just say to them, I'm sorry I've got bad period symptoms and then they're very understanding and because you know quite a few of them are in the same boat as me as well. So yeah, I think that's where that's really good that we can then you know talk to each other and help each other in that way.

0:11:20.90 --> 0:11:34.680
*Interviewer*
Have you ever had any apart from obviously bit of school and stuff when you first started your periods have you had any kind of guidance information around the menstrual cycle and particularly as an athlete and how you should train or manage the menstrual cycle?

0:11:35.580 --> 0:12:12.100
*P11*
I think I have, but not until recently. So, when I first started racing like nobody really talked about it, because this was back in 2011. I, yeah, I didn't, I didn't feel like it was something people spoke about openly. I think you know, it was probably only been maybe the last 2-3 years that I feel like I've had more help and support with, you know, with regards to my period and how to manage it in training.

0:12:13.260 --> 0:12:23.280
*Interviewer*
You mentioned obviously you had a year where your periods stopped altogether. Did you get any advice and support around that period? Did you realize that that was a bad thing and that you needed to?

0:12:23.860 --> 0:13:28.560
*P11*
No, I didn't at the time because it was my first year training with a coach, so before then I just, you know, done my own training. But that was my first year with the coach and he didn't seem to think that it was a problem. He just thought, OK, you know, she's getting light or she's getting faster. So, you know, not having a period, it's, you know, it's fine really and it's like, it's normal for female athletes, which is definitely isn't. But then I remember mentioning it to a good friend who I used to race with, and I said, you know, I haven't had a period for almost a year. And then she told me that, you know, it's not, it's not good, you really need to have one and she gave me a bit of a lecture about, you know, the consequences and you know why having regular periods is important. And then you know after that I realized that I had to get it back. And so, I managed to yeah, I managed to do that thankfully.

0:13:29.740 --> 0:13:35.640
*Interviewer*
Was that by focusing on making sure you had enough, like eating enough to fuel your training sessions was that?

0:13:36.370 --> 0:13:44.380
*P11*
Yeah. Is it? Yeah, exactly. So, I think that was the main thing. Like it wasn't during that year when I didn't have my period it's not that I wasn't eating, but I was just eating very clean because that's what my coach told me to do and because it was my first time training with the coach and because I was still new to the sport. I just, I didn't really know any better but I think it was, it was just making sure I had more, you know, calorie dense foods and like just fuelling for the training I was doing.

0:14:11.490 --> 0:14:36.130
*Interviewer*
You mentioned that kind of that in terms of people having conversations around periods, menstrual cycle is really like kind of got better over the last couple of two or three years. Are you? Have you been aware of any of the stories, or the elite athletes that have talked about their periods and maybe effects on performance. I am thinking people like Dina Asher-Smith or Eilish McColgan. Have you seen any of those articles? Has that made you reflect on anything?

0:14:36.810 --> 0:15:26.580
*P11*
Yeah, yeah. I mean I think it's amazing that world class athletes are sharing it with everyone. Because I remember, like, you know, back in the day when it wasn't very widely spoken about, I felt like there was something wrong with me when I didn't perform well in the race because of my period. And I would, you know, I would beat myself up about it, whereas I think, you know, the more people share, the better it is for female athletes because they feel like they're not alone. And yeah, and you know, I just, I just think it's good to read about their experiences. I mean obviously it's not nice when your period negatively affects your performance, but it's good to know that you're not alone.

0:15:31.460 --> 0:15:39.970
*Interviewer*
That's all my questions around kind of periods and the menstrual cycle. Is there anything else you'd like to add that you think I missed that I didn’t, or we didn't talk about?

0:15:41.80 --> 0:15:51.780
*P11*
No, I think you've covered it very well. Can't think of anything at the moment, but if I do by the end of the interview I'll let you know.

0:15:51.380 --> 0:15:53.800
*Interviewer*
Yeah, I'll ask. I'll ask you again at the end. So don't worry.

0:15:53.780 --> 0:15:55.510
*P11*
Ask me at the end. I might come up with something.

0:15:57.90 --> 0:16:22.190
*Interviewer*
So moving on to bike set up, equipment and stuff. And I think from your pre-interview questionnaire, you said you just kind of I suppose ride unisex bike and kind of unisex saddle, you haven't made any, your not women's specific. Have you made any modifications to your bike to help fit your position from when you bought it? So, I'm thinking like stems, crank length, handlebars.

0:16:23.120 --> 0:17:7.550
*P11*
Yeah, so I did, when I first bought my bike, my handlebars are very wide and which I think is quite standard like especially when you buy a unisex bike. I think my handlebars were like 40 or something, 40 or 42 which is way too wide for me. So, I got some narrower ones. In terms of my saddle, I mean basically when I bought the bike, the shop I bought at, like they did a bike fit for me and so they adjusted the saddle and the handlebars and everything but I haven't made any tweaks to it myself.

0:17:9.630 --> 0:17:18.390
*Interviewer*
Do you suffer from any cycling overuse injuries? So, I'm thinking lower back pain, knee pain, saddle sores, foot pain or hand or finger numbness.

0:17:19.240 --> 0:17:35.370
*P11*
Not that I can think of no. No, I don't. I think, I mean I've had saddle sores in the past with other saddles I've used them, but now I seem to have found one that works for me.

0:17:36.640 --> 0:17:44.0
*Interviewer*
Yeah, that's one of my questions. How did you find your chosen saddle? Was it trial and error? Was it recommendations? Bike fit?

0:17:43.650 --> 0:18:1.440
*P11*
Yeah, I think it was just, you know, basically trial and error and recommendations as well really because as I said like I've tried saddles in the past, that just didn't work out for me at all, you know, I be one the bike for five minutes and just get very uncomfortable.

0:18:2.990 --> 0:18:18.480
*Interviewer*
Obviously you do a lot of indoor racing which can be one of the I suppose factors that help make you have more saddle discomfort. Are you fine in terms of Zwift? Can you race quite a long time on the turbo? Does length of race have any effect on how you feel?

0:18:20.670 --> 0:18:53.790
*P11*
No. I never really get saddle sores like I do sometimes four or five hour turbo sessions on Zwift. And I never, apart from like the usual fatigue that you get from doing a long ride, I don't notice any saddle sores, it's no different than doing say 4 hours on the road and I have had saddle sores in the past but with this saddle I have now, I've never noticed it really.

0:18:55.240 --> 0:19:2.670
*Interviewer*
With previous saddles when you have had saddle sores have you had to take time off the bike for those? Or have you just kind of pushed through?

0:19:1.610 --> 0:19:11.790
*P11*
No, it's never been so significant that I have had to take time off the bike. I've had various different creams that I have put on the saddle sores which has helped. But you know, as soon as I've noticed discomfort, I've changed my saddle. It's just a bit annoying, like when you don't find one that works for you which I know is the case for many women.

0:19:34.420 --> 0:19:42.390
*Interviewer*
In terms of you said sometimes you treated them with cream, did you have like kind of chafing or was it more like kind of spots or little cysts that you treated?

0:19:42.460 --> 0:19:44.330
*P11*
It was more like a spot.

0:19:45.170 --> 0:19:45.430
*Interviewer*
OK.

0:19:45.20 --> 0:19:52.70
*P11*
I've never, I've, I don't think I've ever had chafing. It's just been like the little spots really.

0:19:53.190 --> 0:20:0.70
*Interviewer*
Where would you get those? Would it be kind of upper thigh, outer labia, more round your sit bones?

0:20:0.120 --> 0:20:8.150
*P11*
Yeah, I'll say outer labia mainly. Yeah, that's where I tend to get them.

0:20:10.360 --> 0:20:26.340
*Interviewer*
OK, I think because you're in a fortunate position where you don't suffer anymore from saddle sores and overuse injuries, I don't have any more questions on that, so unless you have something you think I needed to ask you about around this.

0:20:27.780 --> 0:20:39.350
*P11*
No, I don't think so. As I said, I can't come up with anything now and, but if I do, I mean I'll drop you an email if I come up with any other questions.

0:20:42.880 --> 0:20:54.540
*Interviewer*
Then my final question is, are there any areas you would like researching in regard to female cycling? So maybe menstrual cycle, training, bicycle setup, saddle choice, injuries.

0:20:57.680 --> 0:20:59.480
*P11*
Oh sorry, can you say that again?

0:21:0.170 --> 0:21:4.740
*Interviewer*
Are there any areas you would like researching with relevance to female cycling?

0:21:6.130 --> 0:21:41.420
*P11*
I think I mean just the areas you've mentioned really. Obviously there is more research in those areas now, but I still don't think there's enough really because I find with the coaching like many coaches, they just treat, they just say that women should train like men which definitely isn't the case. So, I think there's definitely more research needed in that area. Also like you know what you are doing at the moment with saddle sores and the menstrual cycle as well.

0:21:45.100 --> 0:21:50.30
*Interviewer*
OK, great. Is there anything else you'd like to add before I switch the recording off?

0:21:50.870 --> 0:21:54.280
*P11*
No, I don't think so, no.

**P12 – interview transcript**

***Interviewer*** 0:06
So I'll just start with a bit of background about you and cycling. So how long have you been cycling and what events do you compete in?

***P12*** 0:18
Cycling about 2.5 years and I compete in road races, duathlons, time trials and hill climbs.

***Interviewer*** 0:29
What are your goals for this season? Have you got any targets, racing or training wise?

***P12*** 0:35
I've got a couple of road races coming up and there's a national hill climb which my club is hosting. and a couple of duathlons, but nothing sort of major. I think I did a lot of that last year, but sort of, yeah, a little bit less focused this year to be honest with work and things sort of taking it over.

***Interviewer*** 0:51
Yeah, because it's quite intense. So, moving on to the menstrual cycle, so I know you said, you've mentioned previously you don't get regular periods, but I think on your form you did say you did start your period around 16, is that correct?

***P12*** 1:06
Something like I can't have exactly 15-16 ish.

***Interviewer*** 1:08
No, that's fine. So, what was it like for you then? Were you playing sport? Did it have any effect?

***P12*** 1:14
So I was rowing. I was a rower. I don't remember it having any particular effects and it wasn't regular at the time then either so, but I can't remember having an effect, but I mean I'm old now, so it's quite a time ago.

***Interviewer*** 1:31
Did you get any symptoms at the time, like cramps or anything like that?

***P12*** 1:36
Not really. They're always quite light, so I was quite lucky. I think occasionally I'd have like sort of a day of cramps and things, but I think generally I got off quite lightly.

***Interviewer*** 1:43
Around that time, did you get any education on periods, menstrual cycle or just the kind of standard school chat that you got?

***P12*** 1:53
It was at school, but I think they did quite a good job of going through things, both some sort of like a scientific level and from like a sort of more social pastoral level as well. We had quite small groups. So, our that's called like your form class, so that it was all girls and there was sort of eight to nine of us in there and so, you could have quite open discussions about it, we did. I do remember having those. So yeah, we did get quite good education about everything.

***Interviewer*** 2:18
As you've got older, how has your cycle changed? I think you mentioned you went on the coil in your 20s, is that right?

***P12*** 2:27
I did. Yeah. So, I went. I had the pill for a while which made things more regular and then had a Mirena coil for the best part 10 years, so stop things completely. Then have that removed coming up to two years ago now and nothing started again. That's a whole other story, but.

***Interviewer*** 2:46
In terms of like when you're on the pill to that give you any I suppose side effects? Was that one of the reasons you changed to the coil or was it just easier to move on to the coil?

***P12*** 2:59
Oh, it was purely out of laziness. Yeah, it was easy. It was more convenient for me, and I was not the best person to remembering to take medication. It didn't give me side effects as such, but it was just convenience really.

***Interviewer*** 3:13
I presume that the coil obviously stopped your periods, but it didn't give you any other symptoms or side effects? Any mood related things or?

***P12*** 3:20
No, I mean no, it was, you know, for the first few days having it put in was uncomfortable sort of cramping and things but that was it. And then when I had it changed the same and that was it.

***Interviewer*** 3:32
So presumably because if you haven't had periods that hasn't, it's never really been an issue for you around competing or training or having to factor any of it in?

***P12*** 3:41
No it hasn’t. The only thing I remember at school is that if you were on your period and there was swimming lessons, that was always a bit of a problem but that was it. That's the only thing I can think of, and that wasn't very common so.

***Interviewer*** 3:52
Just one question to ask in terms of like kind of menstrual cycle, periods not returning, has anybody ever suggested anything to do with exercise being an effect on maybe there not being regular or starting?

***P12*** 4:04
Absolutely, yeah, fully aware of that unfortunately. I've actually been. Yeah, I've had and lots and lots of tests. It's probably related to body fat levels and things like that. I have tried changing that, I've tried putting back the next. I mean, that's one of the reasons I've cut back in competitions and things. Not that, I particularly want that to go much further, but I assume this is I know this is confidential you've said,

***Interviewer*** 4.26

Yes

***P12*** 4:27

But it didn't change anything and actually now having treatment ovulation induction for the last few months, I think through that as well.But so, yes, I'm aware that that it's probably having an impact, but having changed the things that I could, it didn't change anything, it still didn't return so.

***Interviewer*** 4:44
OK. Yeah. No, that just thought I'd ask because, yeah, this is endurance cycling, and other participants have suffered similar.

***P12*** 4:47
Yeah. And of course, it's very, very relevant. And it's. Yeah, yeah, it’s a hot topic at the moment.

***Interviewer*** 4:53
So yeah, so I suppose in terms of that, obviously you've seen specialists. Have you had any support, I suppose outside of that kind of related, I suppose round nutrition and things to try and help with menstrual cycle or is it just been through kind of NHS and medical?

***P12*** 5:11
It's been through NHS and yeah, I mean, I don't know, probably getting cocky, but I like to think that I'm capable of understanding the science behind it being. So no, I haven't had any sort of formal support with this, no.

***Interviewer*** 5:24
Are you aware of I suppose it's become greater issue in the media around athletes talking about menstrual cycle, periods, also in endurance sport effect of RED-S thas that kind of raised your awareness of the issues or as you've done medical training were you already aware of it.

***P12*** 5:41
Yeah, I was already very aware of it I suppose.

**BREAK IN INERVIEW DUE TO INTERNET CONNECTION**

***P12*** 8:44
Sorry, you were talking about RED-S.

***Interviewer*** 8:56
Yeah, and I was just saying that I was asking about had, I suppose stuff in the media made you do some more in and I think, but obviously you're a doctor, so you probably well aware of kind of some of the issues, particularly in endurance sport, whether that had made you, yeah.

***P12*** 9:10
Yeah. Yes. Yeah, it's like we do cover it slightly at Med school. It's not a sort of talk about a huge amount, and I think it's quite subspecialist area, but I've been a runner from the age of about 20-21, obviously it's very prevalent in runners as well, so became much more aware of it through that. So, I have done a lot of reading about it myself I think more than through medical training, but you're right, it's become more and more, I think more, more hot topic and it's talked about more in the media and the news. So yeah, becoming more aware of it. I think other people are becoming more aware of it too.

***Interviewer*** 9:46
I think from your form you don't have a coach. Have you ever been coached in the past, does that ever been raised as a question?

***P12*** 9:53
So I have been coached in the past in sort of at school and at university, and actually at running we do have coaches as well, but it's never ever been raised or talked about. My coach has always been male. I don't know if that's had. I don't know. People find it awkward to talk about, but you know I coach has never spoken to me about it.

***Interviewer*** 10:13
Yeah, that's all my questions around the menstrual cycle is or anything else you think I should have asked you about or you'd like to add before I move on?

***P12*** 10:23
No, thanks

***Interviewer*** 10:25
So next moving on to kind of bike equipment and injuries. I think you ride a variety of bikes. I think your main race bike is just as suppose a unisex. Is that right? Is not women's specific?

***P12*** 10:39
That's it's a. Yeah, unisex, men's.

***Interviewer*** 10:43
Have you made any changes to that to suit your position, so I am kind of thinking, crank length, handlebars, stem, any of those things?

***P12*** 10:51
Yes, so it's got a small, I can't remember the measurement really. Sorry, I just have.

***Interviewer*** 10:55
No, I don't need to worry about the measurements.

***P12*** 10:58
It has narrow bars. The crank length is the same as it was, but it's a mens small or extra small.
It's a 51 anyway, and so cranks are already quite short. I can't remember what you have to forgive me. I think it might be 170 or one. It's the shortest one that's standard anyway, but so yes, have adjusted it slightly. Then obviously with the bike fit and had everything sort of moved around and handlebars dropped and things like that.

***Interviewer*** 11:24
Have you ever suffered? I know you've been cycling for about three years. Have you suffered any kind of overuse injuries? So, things like foot pain, knee pain. lower back pain and knee, hand or finger numbness.

***P12*** 11:37
Numbness. Yeah, in my hands which usually returns pretty quickly after the ride. But I remember when I first started and was riding a different bike actually, it didn't fit me as well I used to get numbness that would sort of last for a couple of days if I've done a long ride. Always came back and it was never sort of severe it was just reduced sensation but so I do remember having that. I've never had sort of any muscular injury from it, no.

***Interviewer*** 11:59
Have you done anything to help with the kind of hand and finger numbers in terms of position, gloves or anything like that?

***P12*** 12:10
Gloves. I've always got gloves, always. I go out it's 20 degrees and I have gloves just in case. Yeah, I have always got gloves. And then I think I've learned to try and move around a bit more. So, I feel my hands start to go numb and I'll sort of move on to the drops. I'll move towards the sort of centre of the bars, or just take them out of it and that helps. Yeah, and yeah, keeping them warm.

***Interviewer*** 12:32
In terms of saddle, I think you have got women specific saddle on your bikes, I suppose how did you arrive at those saddles? Was it trial and error or recommendation?

***P12*** 12:44
I did a lot of googling and then tried a few different saddles that were recommend on there and just getting on really well, well pretty well with the Selle Italia one that I've got now.

***Interviewer*** 12:57
I think you just ride a road bike. You don't ride TT bike.

***P12*** 13:01
I don't know. Not yet, anyway. We'll see if my husband lets but.

***Interviewer*** 13:06
Have been having ever had any problem with saddle sores, so either just pain, discomfort, chafing, spots, any of those.

***P12*** 13:13
Yeah, let's say, yeah, fairly regularly maybe like I’m riding a lot. So every couple of weeks or so. Yeah, there's always some level of sort of discomfort. But I do use chammie cream as well, and like Lanacane too, just anything that I can really try and prevent it. But yeah, it just happened. It's kind of just have accepted it as just part and parcel of it, I think.

***Interviewer*** 13:36
Obviously you said you ride quite a lot. Is that a factor that affects it, so length of time on the bike or are there other factors that influence it?

***P12*** 13:45
Definitely the length of time, you know, if I come back from a sort of 7/8 hour ride, I'm much more likely to have done something then if I just go out for a couple of hours. Also, I think it depends how hard I'm going. I think the easier I'm riding, the less pressure I'm putting through my legs therefore the more goes through the saddle, so the lazier I get, the more I'm sort of like putting pressure on the saddle and that tends to have an impact too, I think, but I don't know if I'm reading too much into that, but yeah, if I'm if I'm going quite hard and putting a lot of effort, so obviously my legs take more weight, I think. I don't know if there's anything behind that, but I think I think I've noticed that, but I haven't journaled or kept a diary about it or.

***Interviewer*** 14:21
Does racing have a difference, so sitting, you know, you just ride a road bike, but sitting in more aggressive road position have effect on whether you're more like to get a saddle sore? Or is it that not really a factor?

***P12*** 14:33
Haven't noticed it, but my skin suit has you know, the pad is thinner, so that definitely does have an impact. But that's like the race kit so you have got to wear it really. So, I have noticed that, but tend to move around quite a lot, especially if I'm racing, I'll definitely move around a lot, so actually I think in some ways that's better, maybe.

***Interviewer*** 14:56
In terms of the type of saddle sore you get. So, what type of saddle sore? Is it just pain, pressure or is it more chafing, spots, kind of cysts, that kind of thing?

***P12*** 15:06
Yeah, it like cysts usually and then sometimes some chafing as well, yeah.

***Interviewer*** 15:11
So what do you do if you get one? How do you treat those? Do you just take time off the bike, or do you use repair cream or something like that?

***P12*** 15:18
I don’t use repair cream or anything but I try to give it some time off the bike. Or if I'm on the bike, then I'll adjust my position to try and sort of give us a chance just resolve itself in a few days usually and with chafing just be really careful with using chammie cream and stuff like that.

***Interviewer*** 15:28
Have you ever had to get any medical attention for any saddle sores?

***P12*** 15:31

No

***Interviewer*** 15:38

So, in terms of where they actually occur is it sit bones, upper thigh, is it outer labia?

***P12*** 15:46
Sit bones

***Interviewer*** 15:47
Sit bones. In terms of trying to avoid it, I think you said you, kind of just accepted it. Have you giving up try to find another a better saddle or different shorts, chammie, that kind of thing?

***P12*** 16:01
This I found this saddle. I have tried different shorts. I go through phases of having favourite ones but yeah, I think the saddle I've got I'm generally quite happy with, it’s the best on that I've had. Maybe I should keep looking, I don't know.

***Interviewer*** 16:20
In terms if you get a saddle sore does it your influence enjoyment, as I said, do you have to take time off? Alter your training around it, or do you just kind of crack on regardless?

***P12*** 16:32
Yeah, I just crack on with it. I mean definitely affects the enjoyment. It's much more enjoyable if it's completely pain free, but yeah, I tend to just get on with it unless it's really bad, but.

***Interviewer*** 16:39
Yeah. Have you done anything apart from saddle, have you had, like, bike fits or anything if you mentioned it in that to try and help, or I think you said your current bike is more comfortable, like the one you ride.

***P12*** 16:53
Yeah. So, I have had a bike fit but haven't had anything changed since then and at the time that I had the bike fit it was I was fairly new to cycling anyway, so I hadn't mentioned that, there wasn't really any problem then. So, I haven't changed my position or anything like that.

***Interviewer*** 17:15
That's all my questions on kind of saddle sores is there anything else you would like to mention?

***P12*** 17:23
No

***Interviewer*** 17:24
My final question, are there any areas you'd like researching in regard to female cycling? So, anything around the menstrual cycle, female specific training, bike set up, saddle choice, injuries, anything around that?

***P12*** 17:38
I don't know. I guess I'm quite interested to know what sort of you know what sort of prevention of saddle sores I would be interesting to read advice on. And I guess you know the RED-S thing, but I think that's being very well researched and documented and you know I think it's just the ongoing awareness and conversations about it. I mean, you said like coaches ever talked about it and they didn't, but maybe it should be part and parcel of training to be spoken about openly, but I think that is changing already. I think things are things are changing so.

***Interviewer*** 18:13
OK, that's great. Is there anything else you'd like to add before I stop the recording?

***P12*** 18:19
No

**P13 – interview transcript**

*Interviewer*

So just to start, a bit of background about you and cycling. What events do you compete in? How long have you been cycling?

*P13* 0:13
So I started about seven years ago with getting a bike.Started spinning at the gym, actually, first of all. Then got a bike to do the coast to coast. I’d done it on a mountain bike before, mountain bike and just normal shorts, and that was not comfortable at all. So, we got road bikes this time and I got some padded shorts and that was probably, that was 2017 when I got the road bike. I did some spinning first of all, then did that, and then just kept doing recreational cycling, so cycling with friends or there's a little group I don’t know if you've heard of them? They're kind of they're not club, but there are groups that meet on a Tuesday night, go out for a ride with a different pub each time.And then just all go outside out separately and then come back together as a group.So, I just kept doing that and then I used to do a lot of running. That was my main sport.My husband joined cycling club, he really got into cycling, initially he got into running, got bored of running, so he joined a cycling club and kept saying when you going to join, when you join us, like, well, I'm just doing lots of running. I was doing Ultra running, so just didn't have something else as well.So, I said after I had a big ultra race planned and I said after that, then I'll join and then like less than a week after that race I'd agreed to come along to a cycling club.So, then that was last June that I first went with them. Didn't have particular plans, but I just I went long to time trials and watched him do things, I thought was quite fun.Like it's nice atmosphere. It's kind of thing anyone could have a go out and it wouldn't really matter.So, then I did a couple of races last autumn and really enjoyed them and was more successful at them than ever been running which kind of, quite appealing as well, I think.So, then I thought, well, I'll do a full season this year and just see how I get on. See what I want to keep doing. I had wanted to do maybe do some on the road racing some of the crits, but I don't like crashing, so I think, and there's no local crits this year either there was some early season ones last year but so, I think I'll just stick with time trials and then yeah, it goes.

*Interviewer* 2:10

How's your training and racing going at the minute?

*P13* 2.11

I have done a few sportives over that time when I was recreational cycling…But training, yeah, it's going well. It had a lot of, in that time, and while I was doing recreational stuff was when I kind of sorted out things like saddles and comfort and kit as well and so, I, so I suppose I was quite used to training from a running point of view, so, it's sort of fitted in, it's hard to.I prefer the, somehow running is a more social support, so like when we go to training then we're just all running together.I suppose cycling we still train together, but it's we don't we train in the same way as running club’s train. I think people do their own thing a bit more, so I have sort of missed that, but it's been nice getting to know some people through like through the races and things and through the get to know people a bit more is make it feel a bit more sociable as well.I keep trying to decide whether to get coach or not, or what to do, but I think I'll stick with what I'm doing at the moment it seems to be working for me for.

*Interviewer* 3:16
So moving on to talk about your menstrual cycle, thinking back to when you started getting your periods, what was it like for you? How old were you at the time?

*P13* 3:29
I think it was. I was asking my best mate from school because I really can't remember. She was 13.5 and says I was before her, so I reckon I was probably about 12. It was OK and I was in a girls school, so that made definitely, made it easier and it would otherwise been. They were quite, quite regular and not too heavy, so don't they didn't have a massive impact then, but they got heavier like as I got sort of in my, maybe in my 30s they started to get really heavy like pretty impractical and then I went on the pill, partly just from a contraception point of view, but partly also to try and see if that would help with that, which it did. So, then that over the next kind of seven years or so that I was on the pill that they kind of reduced down to nothing, that was much more practical, and had a lot less impact on things. Yeah, I felt like I suppose generally over the time that I was doing a lot of running when I was having normal periods before I would feel quite bloated, was probably the main thing and just felt a bit heavy and lethargic and not really up for doing anything too competitive, as well as have been impractical as well. Probably mood swings was probably the worst thing that other people would have noticed that I never quite put my finger on because it wasn't every month, so, if it had been a month then you could think that's what it is, but it was more like every maybe every four months or something that I would just feel like there's the end of the world and just nothing, no one could say anything, right? and it was just, you know, and then eventually it's figured it out, ah that's what that is.

*Interviewer* 5:01
In terms of mood swings was a certain part of your cycle was it like kind of that premenstrual phase before your period that was a problem or during the period itself?

*P13* 5:09
Mainly premenstrual and maybe the first couple of days of the period, yeah.

*Interviewer* 5:13
So just going back to when you start getting periods. Were you playing sport when you were in your teens? Did that have any effects getting periods on like, yeah, participating in sport, competing?

*P13* 5:26
I don't think it did really. I still did my running. Still did races. No, it was more like that sort of bloated feeling. Just not feeling as up for it in that time, but I still would do what I wanted to do. It didn't stop me doing anything I wanted to do.

*Interviewer* 5:43
When you were a teenager, did you get any advice around periods, menstrual cycle at that sort of time, maybe school, mum, anything like that?

*P13*

No, nothing at all. It was, you know, the magazine Just 17. So, I have a copy of Just 17 and it had a something that you could send away to get a free sample of lil-lets which is still used it now because of Just 17, so I sent away and I got my free sample of them and then they arrived and my brothers was like what are they? I'm not telling you if you don't know, I'm not telling you. And then, but no, me and my friend just figured it all out. Well she probably figured and I copied her.

*Interviewer* 6:21
Obviously you mentioned when you were running that you're kind of just maybe feel a bit bloated and get on with it. Did he ever make any kind of concessions to training, to train around symptoms, or did that not really have an effect?

*P13* 6:34
It didn't really register with something that anybody did or that I would have done, if you know what I mean. It's just kind of on this is didn't really talk about it much. My family don't talk about anything like that as well. And don't really talk about it and you just get on with it and so didn't. Yeah, didn't let it affect anything really. Even if it would be better to have done.

*Interviewer* 6:54
When you were doing Ultra running obviously you would be out for a very long time, did that ever have a problem if you were on your period during an ultra or did you get lucky and it never fell on a ultra.

*P13* 7:03
I was pill mainly for all of those. So, they, so that was really good because it would been nightmare the way it was before. I wouldn't have been able to do them because it was too heavy and needed to change the tampon too often, it just would have been, I couldn't have done it. So, I wouldn't have done, but by the time I was doing them, I was with the pill, it had pretty much fizzled out to nothing, really. So, it didn't need act, as long as.

*Interviewer* 7:28
Did you get any mood related symptoms on the pill or were you totally fine with that?

*P13* 7:37
Yeah, I was totally fine. Like the first time I took it, I had. I was that night. I was in absolute, I woke up feeling in agony, went in the bathroom felt so much pain, lay on the bathroom floor for a bit and then this is ridiculous I have got to get up. And since then, I never had any problems, so most have been a hormone adjustment thing, I don't know. Yeah.

*Interviewer* 7:55
Then obviously in the prequestionnaire you said you've come off it for breast cancer. How are your periods now, do they affect you in your cycling?

*P13* 8:08
So they've gone. I'm not sure quite what they doing, they're in a bit of a flux phase now, so they started to get, they came back and then they started to get heavy again. So, I think they'll probably go in the direction of how they had been before but then I started the tamoxifen, because that can apparently bring about a sort of chemically induced menopause. So similar symptoms to menopause. Your period apparently would still come back when you come off it. But so, I'm not sure what they're doing, which is why I was started tracking my cycle because I wanted to know. I didn't know what to expect. You know how heavy things were or anything to see if it has any theme. So yes, they've come back, but that's reduced them down to a manageable level. And I haven't really done any long stuff when I've had my period I don't think training wise, just the sort of four or five hour rides, but nothing kind of all day things and that's just been.

*Interviewer* 9:01
Yeah. Is that like a conscious choice to try and avoid long rides during those kind of maybe couple of days where it's more, it's heavy to avoid having to have a toilet stop?

*P13* 9:13
It's a difficult I would, I probably wouldn't, because, I only ride with other people because I can't change a tyre and I can't navigate. So by what people are doing for my riding. So I guess they would, if in theory, if it was as heavy as it can sometimes get, then there might be a day that I would say I couldn't go because I wouldn't, just the side of the road or anything like that. So yeah, and when you with guys you feel a bit more, they keep stopping for wee’s or whatever but I just feel a bit more conscious of, like, it'll take me ages to, like, get my kit off and get out and then on, so I just don't bother normally. But yes, I think it hasn't yet affected and if it's reduced it currently it's, I mean I had last period it lasted for sort of three weeks, but I wonder if that's it tapering off because it was then all but for three weeks so but so that level you can still ride with so I will see how it what they do now.

*Interviewer* 10:05
Have you ever had to take like painkillers during your period when it's heavy bleeding or do you not get symptoms of pain?

*P13* 10:12
Yeah, I have taken just paracetamol and I used to take Neurofen when I was younger, but just ibuprofen or just paracetamol now.

*Interviewer* 10:17
Obviously we have talked about long rides would you ever modifying every other sessions like turbo sessions around it. I know you said your period maybe is stopping like altogether, but would you modify any turbo sessions around that if you're on your period or would you just do as scheduled?

*P13* 10:37
I would just do a scheduled, yeah.

*Interviewer* 10:42
We have talked about a bit about when you're at school getting advice. Have you had any advice subsequently around the menstrual cycle and competing in sport and managing your training around that?

*P13* 10:54
No. No one's ever discussed it, really, and I haven't really read. Read stuff up on it either. No, it's just never kind of really featured on in my radar.

*Interviewer* 11:05
That was one of my questions. Are you aware of the kind, I suppose increased media coverage around kind of the effect of periods on athletes, so the likes of Dina Asher-Smith spoke out, Eilish McColgan, some of the GB cyclists have spoken out about it. Have you seen any of that or?

*P13* 11:22
No, that's all passed me by, I think. There's a lot of menopause chat around, so that's probably more something that I would have looked into or noticed, but I haven't, no, I haven't noticed that at all.

*Interviewer* 11:35
I think that is all my questions around the menstrual cycle unless you've got anything you think I should have asked you about, I've missed, or you'd like to say?

*P13* 11:45
No, I don't think so.

*Interviewer* 11:50
OK, so going on to your bikes, I think are you all women's specific? No, not women's specific bikes, but women's specific saddle sorry to get the right way round. Have you made any changes to your bikes if you've, I suppose you have bought a unisex bike? so talking handlebars, brake leavers, stems, cranks, have you changed any of those on your bikes?

*P13* 12:17
I had a bike fit, so that changed my crank length and handlebar widths as well, again it is one of those you probably speak to my husband, he’ll know exactly what we changed, what we didn't change. I think maybe the stem might have gone down a bit as well but off the back of that there were various things changed but not sort of specifically women specific just from what the bike guy recommended.

*Interviewer* 12:34
Have you suffered from any cycling overuse injuries? So lower back pain, knee pain, saddle sores, foot pain or hands and finger numbness since you started cycling?

*P13* 12:57
I would say saddle is probably the only thing that's really noticeable. Generally, I'm more comfortable than I was running aches and pains wise, but I think the saddle has been has taken quite a while to get to a manageable solution with saddles, bibs and things.

*Interviewer* 13:06
So in terms of reaching that, have you gone about if you just trial and error with saddles, bike fits, recommendations?

*P13* 13:20
So my first ever bike was a Specialized Alley, so I just rode the saddle that came with but then I had padded shorts and wore knickers with it as well, because I couldn't get my head around not wearing knickers when you’re doing it, so that was my first combo, which kind of got me by, but it wasn't comfortable at all. So, by 20 May 2018, that's kind of a year later, I went to Evans and I got a Fabric Scoop. They measured me and told me the widths that I should be getting, which was bigger than what I'd been riding. And they didn't have any insight into kind of women's stuff specifically, but he just recommended this Fabrics Scoop, which I bought and they said you couldn't change them like if you bring it back within a month or something, then you can. So, I bought that one and then, which felt better than the one I had before. So, I hadn't changed it in that time, so then I was stuck with it but over that period, next of couple of years I was riding with that and at some point, over that, I did research into whether people wore knickers or didn't, so I asked all the women cycled and like 50% and 50% didn't. So eventually I managed to accept that that was OK to not wear knickers, so that helped. Then it was May, somewhere in May that I had a bike fit and he said I needed a different saddle because he was looking at the pressure like the for the saddle I was on, but he wasn't very helpful at all in terms of knowing actually what to do. He didn't have any women specific advice.
So I came away a bit frustrated about that. OK, we've had got a problem and he has made all this money, so he should be helping me fix it. I think he was getting irritated. He was trying different saddles that he had and I was just I am not sure, I'm not sure. I'm not sure if that was better than the one before I was being really faffy and did the read about it. Just thinking we are running out of time. So that was that was May 2019 and then by May 2020, so that was like three years after I started riding. I knew I was looking all this up because I got no memory at all, but I thought I knew I needed to get something different, but it was mainly the pressure at the front was the problem. Like the sit bones were OK because the sit bones had been the problem before then and I would get saddle sore pressure areas and things but ultimately the thing that stopped me once I was wearing the shorts right and things, ultimately, it was the pressure at the front that was the problem, so I'd googled and getting loads of information and my husband said I was bleating on about channels according to him and like learn about from Google about amputees and the anatomy and things like how was no like, I've been asking all these people like Evans and bike fit and whatever, and no one mentioned any of this, but it all made sense. So, then I bought in June 2020, I bought a Pro Stealth from my research, but then sent it back and I can't remember whether it was the front or whether actually the back was worse or whether both was worse. But either way, I knew that wasn't going to be the answer. That was when I first decided it was worth, I’d spend any amount of money to have a comfortable saddle by then. I sent that one back and then the following month, so July 2020, I bought my Power Pro Elaston which is the best thing I've found so far. So, I've stuck with that. It didn't. They've stopped selling them now, so when I've got the bike, I've had to get the Mimic Elaston Mimic, but that seems to be OK as well. Then I bought my first bib shorts in 2020 as well. So again, it was three years later. So, it's kind of been a journey and it's been, it has been really frustrating because I felt like there's so many bike things and you just can't get the information about what to do about it and Google's limited. I learned stuff. I was like, this is interesting, it makes sense, but I've still not got an answer about what I thought the Pro Stealth from all my research was going to be the answer, but it just wasn't, so I guess everyone is different.

*Interviewer* 16:43
In terms of saddle sores are there anything that makes it worse or more likely to happen? So, kind of longer rides, turbo, like that kind of more aggressive position on the drops or low down on the hoods, any of those affect it?

*P13* 17:01
I think it's mainly the longer rides. I think it's time in the saddle thing. Yeah, that's the main thing I've noticed. On the turbo trainer, on the hoods, on the drops sorry, would be worse as well, but I don't do a lot of time like that, so that would be that would be more pressure on the front. But I don't notice that I'm racing. I think maybe your brains just not, maybe haven't done long enough races for that to be a problem or whether your brain just so focused on the pain in legs or elsewhere, but you don’t notice.

*Interviewer* 17:26
If you ever put a tilt on your saddle, so slight nose down to relieve pressure on the front, anything like that?

*P13* 17:38
Yes. Yeah, we've been in. My poor husband has been like constantly adjusting it like up and down, base, like, no, I'm sliding off the front now, so put it back, you know, like trying to get the right the right point for me. Yeah, I move it backwards as well.

*Interviewer* 17:53
In terms of like the type of saddle sores you get, is this kind of spots, little cysts, more like kind of pressure, chafing, bruising. What sort of saddle sores do you get tend to get?

*P13* 18:06
The ones in the back, like round sit bones is kind of sort of blisters, or like sort of little lumps where its sore, and you feel like it would ultimately wear through eventually. But at the front, it's more of a pressure, end up a bit swollen and just really tender, but it's more the pressure feeling for the front.

*Interviewer* 18:25
Is that kind of upper thigh, outer labia that you suffer the kind of pressure in terms of your saddle sores?

*P13* 18:34
It's not thigh, it's further up, it's more around your like private parts. Yeah, like, all the inside.

*Interviewer* 18:43
Is it just pressure or have you ever had any chafing from that or was it just kind of pressure and bit swollen?

*P13* 18:51
I think, uh, I don't think I've ever had chafing. It can be red, but from the sort of heat and pressure but I don’t I've ever had chafing there.

*Interviewer* 18:57
Yeah. I am in terms of if you get a saddle sore, how do you cope with it? Do you just crack on and keep riding, do you take time off the bike, have you ever had to take painkillers or user I suppose kind of the repair creams you can use if there more the spot, like cyst type.

*P13* 19:17
I found, we got a this this thing here [shows chammie cream], we got that free from something like in some or something, and that's really, really good. So, I keep using that and I just bought like bought another one to replace it, but that's brilliant for. Now I would use if ever I'm going out for a long ride, I put it on before, sometimes even just for turbo trainer session, for a race, just like any time before, but also just use it as a sort of therapy for cream, soothing cream. I found that to be the main thing that I would say that's made a difference treatment wise. I don't think I've ever taken painkillers for it. It would have been borderline whether I needed to, but I don't think I have.

*Interviewer* 19:59
Have you had to take time off the bike if you've had one?

*P13* 20:03
Not from the point of view. I wouldn't then get on another day, like I might have wanted to cut a ride shorter based on the fact that I'm not hacking it, but I wouldn't, I've never then stopped riding or not done a ride I intended to do, but just by whacking on the cream.

*Interviewer* 20:19
In terms of like training or competition does it affect you? Does it affect your enjoyment? You think it ever affects like your power output if you've got a saddle sore?

*P13* 20:31
No, I don't think so. But partly, I suppose, because I haven't been racing long, so I haven't had a chance to spend do much of the longer stuff or to see how. Yeah, like if I was doing 100 mile one for example like something, I don't know that I could be a whole different kettle of fish for pain. But I haven't. I haven't tried anything like that yet or yet, maybe never, who knows. But it is hard to know about power output again because I haven't, I've only I got power pedals, but only just like relatively, like last autumn maybe, so I don't. Haven't even yet quite figured out what my normal power is. It's all still a bit of a learning curve.

*Interviewer* 21:11
In terms of like saddle issues, do you ever obviously discussing with your husband, have you ever spoken to anybody else about them apart from maybe go to get a bike fit and mentioned that you've not had a comfortable saddle?

*P13* 21:23
Just some of the girls from cycling, like friends that cycle and then one of the girls in cycling club I think I mentioned as well.

*Interviewer* 21:31
In terms of, I think you've mentioned that I think you pretty much done all of these on the list. So, you've changed your saddle, changed your saddle position, got cycling shorts, use chammie cream. Is there anything else you've done to try and help alleviate saddle sores or prevent them happening in the first place?

*P13* 21:57
No, no, I think that's. Yes, it's trying to decide about shaving, whether to shave or not shave. That's the thing in my mind as a thing, but I haven't done anything about thinking about it or finding out more about it all working out what's the best plan? Because I know some people have said that some women's cyclists maybe don't shave, I think the guys were joking about it at one coffee stop that they don't.

*Interviewer* 22:18
The answer is don't shave that's the GB cycling advice from researching before the Rio Olympic Games.

*P13* 22:23
Right.

*Interviewer* 22:26
Yes, and I can share some stuff with you afterwards, like proper based advice. But yeah, no, don't shave particularly if you're prone to the spot type of saddle sores. In terms of, that's all my questions around that. Is there anything else? Again, you think I might have missed, or you'd like to tell me about in terms of bike set up, saddle sores, injuries.

*P13* 22:50
I don't think so. I think that's all the things I could remember, yeah.

*Interviewer* 22:56
Finally, are there any areas you'd like researching in regard to female cycling? So, the menstrual cycle, female specific training, bicycle setup, saddle choice, injuries.

*P13* 23:11
I mean, all of those above, definitely, yeah. Nutrition wise as well, I mean, don't know if there's any difference between, because my husband reads a lot of stuff, so I just follow whatever he says, but whether there should be a difference between me just following what he does or whether you know in most specific stuff. Even the psychology of stuff is interesting as well, isn't it? Like, where the again, the sort of everything you read or everything I read is via my husband, but it's all kind of just geared generically, so whether do different things from the psychological approach to cycling that would be useful from a female point of view for racing.

*Interviewer* 23:49
OK, great. Is there anything else you'd like to add before I stop the recording?

*P13* 24:03
No, no, I don't think so.

**P14 – interview transcript**

***Interviewer*** 0:06
So I'll just start with a bit of background. So, what cycling events do you compete in and how's your training going at the minute?

***P14*** 0:18
I'm mainly cyclocross, so that's always, it's usually in the winter. Then, yeah, training is going all right at the minute. I had quite a big break after the cross season, maybe like best part of two months. So, I've been training properly for a month and half I would say and it's going a lot better. In the summer I do bits and bobs. Whatever keeps us going for the for the cross season, tried a bit of mountain biking, a bit of road racing but yeah cyclocross is my main discipline.

***Interviewer*** 0:50
So when would the season start?

***P14*** 0:53
So it usually starts around September and then it finishes late February. So, it's quite a long season.

***Interviewer*** 1:03
So, I presume in terms of targets for this year, it's all geared up towards the cross season starting later.

***P14*** 1:11
Yeah, that's right. September there's like a block in England, so I don't need to be going too well, just that's like the starter block and then the start October, November time, so as long as I'm well for then, then that's good.

***Interviewer*** 1:24
OK, great. So, moving on to kind of menstrual cycle, so thinking back to when you first got your period, how old were you at the time and what was it like for you?

***P14*** 1:38
It was, maybe when I was 15/16, so I was quite late anyway, and I didn't actually have it that long maybe three years till I was 17-18, and then I lost weight, started cycling like a lot more, training a lot more, and then I just didn't have a period for many years but what I remember of when I did have them there were always very light. I've never really had periods.

***Interviewer*** 2:07
So in those three days were the fairly regular at the time before they stopped, or did they start to tail off before then?

***P14*** 2:14
Yeah, I would say they were quite regular and then obviously when I started training a lot more, they did just tail off.

***Interviewer*** 2:23
And around that time when you started getting your period were you doing a lot of sport then? Did it have any effect on you taking part in sport?

***P14*** 2:33
I did do a lot of sport, but not at the same kind of level, like I wasn't training like every day and pushing my body the way that I do now. I don't remember it really having an impact on sport then. I just remember thinking it was inconvenient. I didn’t really suffer with like, yeah, like feeling bad or cramps and all that kind of stuff when I was when I was younger.

***Interviewer*** 3:01
Did you get any kind of education or advice around periods at that time or was it just a kind of standard school chat?

***P14*** 3:12
Yeah, just school stuff, really and I have a twin sister, so we would discuss that stuff.

***Interviewer*** 3:16
When you said you, you probably had a period for about three years, and then they stopped, did you go at the doctors to seek any, to get that investigated at the time or have any treatment?

***P14*** 3:32
Yeah, I think it maybe after a year or a year and a half I went to the doctors, and they did a check up and said it was, they basically just say it's normal don't they. They say you're an athlete, the only way to not, to get them back is to stop cycling because even, like over my time of being an athlete, not having a period I've put weight on, and I haven't had them back, so I think it's because I'm, I think you just push your body so much.

***Interviewer*** 4:05
I think in your questionnaire you'd spoken about how you had been to see a sports gynaecologist and that led you to taking the combined pill I think you're on now. What was that what made it, prompted you after a period of time to go and seek more help?

***P14*** 4:21
I think just like over a period of time, you get ongoing injuries that probably have been affected by not having periods for so long and he explained that all to us. He kind of just said, look, I think if you're not going to get periods when you're pushing your body like this, being an athlete, then you should probably try and get them with the pill, because then you'll have all the hormones and everything going on, your body will still be like working and he just said it's a good thing for athletes to do that. So, I thought I'd give it a try.

***Interviewer*** 4:58
Obviously you've raced for some pro teams have any of them ever asked the questions around your periods or questions about your health or done any health checks around that.

***P14*** 5:07
I've had, like, DEXA scans and stuff, and yeah, health checks, but nothing really related to periods actually or like help to try and I don't know get them back.

***Interviewer*** 5:23
When you've had a DEXA scan has bone density being OK, because obviously that is one of the things that can be influenced by periods.

***P14*** 5:30
Yeah, that was I think it was pretty normal which was a good thing.

***Interviewer*** 5:33
Yeah, definitely. I think, in your questionnaire as well you mentioned you had tried the progesterone only pill like a few years ago was that recommended by the GP to try that?

***P14*** 5:42
Yeah, it was actually for my skin they didn't know that I had hidradenitis then. They just thought I had just bad skin and apparently sometimes taken that can help with your skin but actually I think it made it worse and I didn't agree with it really it wasn't good for us.

***Interviewer*** 6:04
I think you said it affected your mood. Is that right?

***P14*** 6:07
Yeah, I was not myself. I went really negative, and I couldn't control like my emotions very well but with this one, I don't know the first like month I struggled a bit because I think all these like new hormones were inside of us and was a bit overwhelmed with it but now I feel like I'm on top of it, so this seems to be working.

***Interviewer*** 6:31
In terms of now on the pill, do kind of are you getting fairly regular periods? Well for the few months you've been on it. Is that a regular? Do you get any other symptoms like cramps or anything to go with it?

***P14*** 6:44
Yes, it's very regular now, but just I think the week before and maybe like the few days before, I'll be really bloated and just feel I am getting tired but then I remember last time after it, I felt really, really good and then I felt good for a few weeks after that.

***Interviewer*** 7:06
So I think you said that, kind of terms of mood wise it's settled down, is that right?

***P14*** 7:13
Yeah, it definitely has. I feel like I can manage my emotions a lot better, and at least I know now that if I'm feeling sad or if I've got things going in my head, I know, like, oh, probably like hormones. So, I think that's a good thing as well.

***Interviewer*** 7:32
In terms of, obviously it's not long you've been on it. Have you ever had to do any kind of modification of training or stuff around it, or is it just all fine to continue your normal training programme?

***P14*** 7:43
Yeah, I think just when I'm, so the last, I will have had two periods now since I’ve been on it and before them, I've just felt a bit terrible and I've just not done as much, I've maybe made my sessions a bit less intense. I still feel like I can ride as long, just not, I can't push my body as much so I've just done that and then when I feel better, I can add intensity in again.

***Interviewer*** 8:09
I think you said you've currently got a coach, is that right? You have gone back to your old coach, have you discussed any of this?Like your periods, going back on the pill with your coach or is it not that kind of relationship?

***P14*** 8:22
I haven't actually discussed it with him yet, but I kind of for two months after the cross season, I didn't, I was, I didn't really do much, so I just kind of wanted a bit of a break, so it's really only the last month and a bit like I said that I've started again, so I might discuss it with him, maybe in the next few months or so, but it's just a gradual getting him back into coaching us because I've had not lot of structure for a while.

***Interviewer*** 8:43
Have any of you coaches in the past have they ever asked any questions about periods or how you are going, or have you got any symptoms?

***P14*** 9:00
Not really. Maybe, I worked with, do you know Helen Wyman?

***Interviewer*** 9:04

Yes

***P14*** 9:05

I worked with her husband, but she kind of coached us as well. So, but I didn't have periods, so it's, I've never really needed to discuss like anything with them because I've never had them while I've been like a professional.

***Interviewer*** 9:24
And have you ever had any again education or guidance from either your team, or I suppose British Cycling around periods or menstrual cycle?

***P14*** 9:32
Not really, no, no.

***Interviewer*** 9:36
In terms of are you aware that I suppose there's been a bit more chat around periods in the media with some athletes like Dina Asher-Smith, Eilish McColgan have maybe spoken about it. Have you seen any of that? Has that made you go and seek help around not getting any periods?

***P14*** 9:54
Yeah, I think there is a lot more chat about it now. I think like where everyone's starting to realize that it is actually a good thing to have your period, it's not, you have more negatives by not having it then you do. So, I think, yeah, probably all the discussion that's going on and I know Evie Richards has done a lot about it on her, she will post about it on Instagram, and that's probably spurred us to go and yeah, seek help as well, I guess.

***Interviewer*** 10:16
Ok great. Do you have anything else you want to add around periods or menstrual cycle that I haven't asked you about or I think it's relevant?

***P14*** 10:33
No, not really. No, think that's all good.

***Interviewer*** 10:39
In terms of like bike, I think you obviously ride a team issue bike. Is that just, I think you said Ridley is standard, Ridley, it's not women specific?

***P14*** 10:51
I don't think it's women specific, no. I mean it's pink or it's purple. I don't think.

***Interviewer*** 10:55
But in terms of the frame size, like.

***P14*** 10:57
No, It's just the smallest, smaller size.

***Interviewer*** 11:01
Have you made any modifications to that to suit your setup like stem, handlebars, cranks?

***P14*** 11:08
Yeah, I use 165 cranks. So smaller cranks and then I think, I think I might have put shorter stem on it. Then I changed saddle as well.

***Interviewer*** 11:19
In terms of cycling overuse injuries, apart from saddle sores, have you suffered from anything else like knee pain, lower back pain, hand, finger numbness, foot pain?

***P14*** 11:32
Just knees. I had a really bad knee injury. It was maybe four years ago in my left knee and then it came back last year but I've got it sorted. They usually, it usually lasts for like a month and then it went.

***Interviewer*** 11:51
What do you think causes? Is it bike set up or just or?

***P14*** 11:55
Well, I think the one four years ago, I think it was something to do with my pedals. I can't really remember, but I think I like used. I was using SPD pedals for long miles and it was my cleats weren't like tightening or something to my knee was moving, but last year it was, I think like real severe tightness in my quad and then it, I didn't stretch it or get it massaged and it was just pulling on my kneecap for ages and then once I got a needle in it, in my quad to release the tension, the knee pain went away.

***Interviewer*** 12:11
Moving on to saddles, on your thing you said you've been through quite a lot of saddles, and you've just finished with the team and managed to change on to a Specialized saddle. How have you kind of gone through that process of your different saddles? Is it just trial and error?

***P14*** 12:52
Yeah, a few years ago when I first started getting really bad saddle sores, I went through a whole thing of ordering new saddles and trying them out. And then I managed to find the Specialized one that fits really well. And then, yeah, then I moved teams, and I couldn't use it.

***Interviewer*** 13:12
That really surprised me that you were not allowed to change saddle for like health reasons.

***P14*** 13:17
Yeah, teams are like, can be like that though, yeah.

***Interviewer*** 13:22
And in terms of saddle sores, what type of saddle sores have you suffered is it pressure, chafing, spots, little cysts, pain?

***P14*** 13:32
Like more like, mine are like little spots, and then they go, they get bigger and go into boils and then they get infected, so mine are quite severe ones.

***Interviewer*** 13:44
Whereabouts do you suffer from those? Is it sit bones, upper thigh, outer labia?

***P14*** 13:49
Usually right on my sit bone. The one that I had on going for ages was that like, right on my sit bone, like, right in the middle, so that wasn't in a great place.

***Interviewer*** 14:10
And obviously if they've got infected I presume you've had to seek medical treatment for, has that being antibiotics or draining them?

***P14*** 14:18
I've had a few drained actually, and then I've been on various antibiotics, but now I'm on a longer-term antibiotic and I have been on it two years. It's called Lymecycline but then now they've changed it to a different one because they said I might have become resistant to it, so, I'm on a one called, I think diclofenac now, and I think that's just one a day, so it's very low dose but it just keeps my skin at bay.

***Interviewer*** 14:44
Because you use normal chammie cream, or do you have to do something special if you've got some problems with your skin?

***P14*** 15:00
Do you know double bass cream? I use that cause it's got like nothing in. It's basically just a, just like a cream.

***Interviewer*** 15:12
In terms of if you've had a bad saddle sore, I think you mentioned in your questionnaire has that resulted in time off the bike in the past. And yeah,

***P14*** 15:21

Yeah.

***Interviewer*** 15:22

Did you say you'd to had a kind of couple, like couple of months easier? What would you do in the race season? Would you have to miss a race, or would you try and race through it?

***P14*** 15:34
I've tried both. Usually when I have a flare up or a saddle sore its because I'm running down and it's because I'm tired or I'm stressed or, so I've found if I do just take time off, it does help it go away quicker because then I get less run down, but I have also trained through them before as well. Just I've done lots of out the saddle like turbo sessions. Lots of running. Actually, I did like 2 weeks of cyclocross training like out saddle which wasn't great, and I managed to race through it and stuff. So, you can. I can do that but probably just means that it stays for longer because I'm not getting, my immune system's probably even lower.

***Interviewer*** 16:25
When you've raced through it, have you done anything to help? Have you had to take painkillers or anything to help?

***P14*** 16:29
Yeah, pretty much. Painkillers, lots of caffeine before the race. Usually in a race I wouldn't feel it in a in a cyclocross race, in a road race I would.

***Interviewer*** 16:47
In terms of like a flare up you have mentioned obviously being run down is a greater risk of it.
Is there anything else that affects it, like long rides or certain positions?

***P14*** 16:56
Yeah, I think probably long road rides would be the most and if I get wet, that's so like today in the rain, made sure I had lots of waterproofs on and yeah, probably just that. Lots of sweat, that's usually bad as well, and I rarely go for coffee rides because I don't like to sit in my shorts.

***Interviewer*** 17:14
I was going to ask you that if you try and avoid sitting in them.

***P14*** 17:22
It's like a rule I never, I never really go for coffee on rides. I'll go and get a shower and then go back out for coffee.

***Interviewer*** 17:28
I presume based on what you said that if you get a saddle sore that it does influence your enjoyment of cycling and training and competing.

***P14*** 17:44
Yeah.

***Interviewer*** 17:45

Since it sounds like you have had a grim period. Do you discuss if you've got saddle sores with your coach? I presume to modify training if needed.

***P14*** 17:55
Yeah, yeah, definitely I do. I actually I had an English coach. It was two years ago now and he was really, really good with them, like he changed all my like training. So, I could do like out the saddle turbos and just he was just really creative with his sessions and I really like that.

***Interviewer*** 18:16
Apart from changing your saddle, have you done anything in terms of I suppose saddle position like tilt or change your bike setup to try and help with saddle sores?

***P14*** 18:27
Yeah, always have a tilt. It's like 2 degrees, I think so, yeah. Tilt, that's essential if I don't have a tilt, then it's just it's uncomfortable. I probably ride with a little bit too low of a saddle but, yeah, if it keeps saddle sores away then that's fine and I do think 165 cranks helped as well.

***Interviewer*** 18:54
And is there anything else you've done in terms of kind of managing it, or in terms of kit I presume you get no choice on cycling shorts and stuff, you just have to wear whatever you’re provided.

***P14*** 19:09
Yeah, pretty much, but I've been lucky with the last two years I've had Vermarc kit which has got a really nice chammie, but now I'm moving to Bioracer which isn't as nice cause it's, like I like a one that's just flat., just no profile anywhere, just smooth all the way around, but Bioracer has got like bridges, so I think I'm going to have to get go to a seamstress and get a few of my Vermarc pads sewn in to the Proximus kit, because otherwise yeah, I don't think it would be good to race.

***Interviewer*** 19:49
That's all my questions on kind of overuse injuries, saddle sores. Is there anything else you think I haven’t asked you about that you'd like to mention?

***P14*** 19:58
Only that for me personally, I usually get more sores when I'm stressed, whether that's physically because I've done more training or whether it's mentally as well but yeah, that's just one thing that I've noticed over the years.

***Interviewer*** 20:15
And as a rider have your saddle sores be managed by a team doctor, or have you had to just put up with the GP and try and manage it through that.

***P14*** 20:25
I've had help over the last few years with the team doctor, but I actually went to see a specialist dermatologist lady in Scotland. Who was she was actually through GB, so it was through cycling actually. But it's tough doing it through the NHS and cause it's not like, it's not, I don't know what the word is, it’s not like a life or death thing.

***Interviewer*** 20:50
Have you got anything else you want to add about that are do you think we have covered about everything?

***P14*** 21:01
Think that's everything? Yeah.

***Interviewer*** 21:03
Finally, are there any areas you'd like researching regard to female cycling? So, the menstrual cycle, female specific training, bike set up, saddle choice, injuries.

***P14*** 21:16
Is there anything? What?

***Interviewer*** 21:17

That you would like researching, like us researchers to look into around female cycling and training and bike setup?

***P14*** 21:19

I mean, I am interested in the whole saddle sore side of things because I've struggled with it for so long. So, if there was any, anything else that yeah could help with that and yeah, I find that all quite interesting only because it's personal.

***Interviewer*** 21:34
OK, great. Have you got anything else, you'd like to have before I stop the recording?

***P14*** 21:49
No, I think that's fine.

**P15 – interview transcript**

***Interviewer*** 0:11
What events do you currently compete in and what are your goals for this year?

***P15*** 0:18
So I do triathlons predominantly, sprint distance triathlons. So that's the 20 kilometre bike ride and since beginning of last year, I'm also part of an indoor team, so, on Zwift. We tend to do weekly team time trials and I joined Zwift Racing League over the winter season as well.

***Interviewer*** 0:33
OK, so do you do you compete regularly on Zwift then? Is that kind of a weekly thing?

***P15*** 0:46
At least during the winter season, it's a weekly thing. In the summer, it's a little bit more hit and miss, obviously depending on if the weather is good enough to ride outside. But I would say from probably late September through to the end of March, it's a weekly thing.

***Interviewer*** 1:01
For this summer, have you got any triathlons that are your target races, or are you just seeing how it goes?

***P15*** 1:07
I have a Triathlon in July, which is my annual target event. I try and get a little bit better every year.

***Interviewer*** 1:18
How's your training going at the minute?

***P15*** 1:20
Not too bad. I need to get back in the pool, really. It's been beautiful weather. So, a lot of cycling, a lot of running, but I need to get back in the pool.

***Interviewer*** 1:29
OK, great. So, moving on to the menstrual cycle, thinking about when you started getting your period, what was it like for you? How old were you at the time?

***P15*** 1:40
I was quite young, probably 11 or 12. I have always struggled with quite extreme cramps and so I went on to the combined pill very early on. Probably when I was like 13 more to combat those symptoms than anything to do with contraception, and I still struggle with cramps these days. It's a lot better because I moved from the pill onto the IUS, so the hormonal IUD. But yeah, I still usually have probably a day, day and half during my cycle where paracetamol or ibuprofen is absolutely my friend.

***Interviewer*** 2:25
Have you ever had the kind of really bad cramps investigated, like with a gynaecologist or you just managed it through the pill and then the coil?

***P15*** 2:32
I've just managed it through the pill and the coil.

***Interviewer*** 2:38
In terms of when going back to when you started, you said, obviously went on the pill very early at 13. Were you playing sport when you started getting your period? Did that have any effect on whether you wanted to do sport?

***P15*** 2:51
Not that I particularly remember, apart. Well, I guess more the social aspect of it, you know, with particularly at school, when you're having to get changed and you're on your period and it's all a little bit awkward and you don't really want anybody to know about and things. So, I guess it was more reluctance from a social side of things rather than that I noticed any symptoms or anything that would have stopped me doing sports.

***Interviewer*** 3:17
Yeah, and going back to that time, did you get any help or advice from anybody? Obviously, you would have seen the GP, but outside of that like kind of mother, school, any kind of education around periods and the menstrual cycle.

***P15*** 3:31
I mean, most of it from my mum to be honest, but I had one of my first PE teachers was actually one of the first ladies that told me that as much as it feels awful doing exercise helps with period cramps and period pain and that is still something that I find true to this day. So usually getting out and doing some exercise even if I'm not very motivated, tends to help on the more extreme days when I've got a lot of cramps and pain.

***Interviewer*** 3:59
In terms of that, obviously you do exercise, but do you modify those sessions? Do you kind of keep them steady or do you just go with whatever's scheduled at the time?

***P15*** 4:10
I try and go with whatever scheduled at the time. I do tend to notice that I am much stronger, so I tend to do things like weightlifting. I meet with a personal trainer once a week and we do you know more strength-based stuff, that works really well. The cardio tends to go horrendously wrong when I'm on my period. I really, I find it a lot tougher, so I'll try and do the same workout, but I find that I'm running slower, or it feels like a much more difficult workout when I'm focused more on cardio stuff.

***Interviewer*** 4:46
Also if you get bad cramps, if you do triathlon, do you ride in the kind of time trial position with the extension bars or do you ride a standard road bike for triathlon?

***P15*** 4:56
I ride a standard road bike. The Sprint distances are usually, sort of everyman event, so you're not allowed the triathlon bars.

***Interviewer*** 5:02
Do you find if you are getting bad cramps and stuff that has an effect in getting in the supposed the more aggressive position down on the drops or low on the hoods or do you not really, or is that not something really consider?

***P15*** 5:16
It's not something I've noticed to be honest that it makes a difference.

***Interviewer*** 5:20
Obviously mentioned you get bad cramps. Do you get any other kind of physical symptoms? Do you get heavy bleeding that goes with that or just the coil regulate that?

***P15*** 5:31
The coil regulates most of it. I tend to get sort of lower back ache as well and I'm prone to headaches and sometimes migraines, and I do tend to find that I get headaches more frequently during my period. So yeah, more, you know, usually it's just if I haven't had enough water but during my period I noticed that I tend to have a headache most days.

***Interviewer*** 5:54
Do you take paracetamol or ibuprofen for that or do you just kind of push through it?

***P15*** 6:01
Paracetamol usually.

***Interviewer*** 6:04
During your period in terms of like managing it, do you go out for long rides or would you because you'd need to find a toilet if you need to change sanitary protection, do you avoid that or you are mainly Zwifter and stay indoors?

***P15*** 6:18
I mainly a Zwifter and I do find that longer rides are more uncomfortable when I'm on my period. So, I live in Germany. I cycle to work as well, like, I commute, which is a slightly different bike, and I find that in general I'm a little bit more tender and the saddle is a bit more uncomfortable when I'm on my period. So, I yeah, I don't tend to do longer rides at that time.

***Interviewer*** 6:47
Do you get any kind of mood related symptoms throughout your period or at different points in your cycle? Or is that not something that affect you?

***P15*** 6:56
Usually the couple of days before I'm due on my period I am a little bit more impatient and irritable, or I find things annoying me that I would usually be able to brush off and that's usually a good sign that I'm that I'm due.

***Interviewer*** 7:16
So you are on the coil, do you get kind of regular bleeding or periods with the coil?

***P15*** 7:22
Yes. Yeah.

***Interviewer*** 7:24
Obviously, if you particularly if you racing Zwift, which I know a lot of people do the race week in, week out through the winter, would you ever not race because you're on your period or would you just race anyway as part of your team?

***P15*** 7:35
I would race anyway. It's an all-female team. So, we tend to be quite open, and we'll say, you know, I'm not feeling 100% today. So, I'm here, I'm turning up for the team, but I might sit at the back a little bit more or, you know, I might take it a little bit easier. So, we share quite openly.

***Interviewer*** 7:54
So you're quite comfortable in terms of with your team talking about these kind of things?

***P15*** 7:58
Yeah.

***Interviewer*** 8:03
In terms of, we have talked a bit about it. It doesn't seem that you really make any kind of modifications to your training around your periods, apart from maybe you kind of cardio performance is a bit down, is that right? And that maybe focus a bit more on strength training during your period is that correct?

***P15*** 8:16
Yes. Yeah.

***Interviewer*** 8:24
In terms of competing say you are doing a triathlon, has that ever had an effect when you've been competing being on your period or in that kind of few days before when you might be a bit more irritable?

***P15*** 8:37
Interestingly, actually last year when I was doing the Hamburg triathlon, I was on the last day of my period, I think. And surprisingly, I had a best ever performance which I was not expecting. So, I, you know, I have done a little bit of research into it, and it does seem to be that you tend to be stronger towards the beginning of the period and then a little bit more sort of cardio and a little bit faster towards the end. And you know, I don't know whether it's sort of now that I'm aware of it, but I am sort of seeing those patterns. So yeah, last day of my period last year and I had a personal best so.

***Interviewer*** 9:13
Excellent. In terms of kind of the menstrual cycle and relating to training and competition, have you ever had any advice or guidance around that, or have you done any research yourself?

***P15*** 9:27
Just a little bit of reading myself. Like I said about, you know, strength training being a little bit better at the beginning of the period but no official advice, no.

***Interviewer*** 9:40
You mentioned you feel comfortable talking to your teammates around kind of if you're on your period or you're not feeling quite up for it, are you generally quite comfortable in having conversations around this, is that if you had to with anybody else or is it kind of just, I suppose close teammates?

***P15*** 9:57
I mean, it's easier with the team because it's all ladies. That’s kind of the purpose of why the team got set up, and actually they're the ones that put me onto your research as well, so that does make it easier. But I have, my personal trainer is male and I've been training with him for about three years and I've had conversations with him as well about, you know, when I'm really struggling with cramps and sore muscles that we sort of see how the session shapes up and he can adjust it as necessary.

***Interviewer*** 10:28
Yeah, and do you, when you go out on the road, do you ride with men or a club, or is it a mixed ride? Or do you tend to go solo?

***P15*** 10:37
Yeah, I'm an antisocial triathlete, so I tend to ride alone because drafting things aren't allowed in triathlon. So yeah, I'm more an antisocial triathlete, yeah.

***Interviewer*** 10:43
No, that makes sense. In terms of being well, obviously in the UK anyway, I'm not sure what it's been like in German sport, but there's been a few elite athletes have started to talk about if they've had a bad performance or race that is maybe related to their period and other kind of talking about symptoms has that happened in Germany? Are you aware of kind of supposed greater conversations, awareness on the topic?

***P15*** 11:14
I don't know if it's German specific because obviously I watch a mix of English and German things like I am much more aware of things like the Global Cycling Network on YouTube have done a couple of things about it. There was a famous marathon runner that ran and sort of was free bleeding and things. So, I'm aware of it in a general conversation. I couldn't say specifically whether that's Germany or just globally. Although actually to think of it, my local gym cafe, the lady that runs it, she's doing a PhD, and her topic is menstruation and sport, so I did a similar survey for her, but hers is more generally about sport performance and not elite athletes as such, but more sort of day-to-day. So, I guess there is there is more interest in it for sure.

***Interviewer*** 12:07
Yeah. That's all my questions on the menstrual cycle. Is there anything else you'd like to add?
You didn't think I asked you about that’s relevant?

***P15*** 12:18
No, I don't think so.

***Interviewer*** 12:21
Going, moving on to the second part. So, kind of bike set up and injuries. I think from your questionnaire you ride two women's specific bikes, but you don't ride a women's specific saddle from my understanding, it’s a unisex saddle, so for men and women?

***P15*** 12:31
Yes.

***Interviewer*** 12:37

In terms of your bike, obviously it's women’s specific, but have you made any other adaptations to it, like handlebars, crank length, stem, or have you kept it as it was?

***P15*** 12:37
Yes. Yeah. My indoor bike I put a riser stem on it, so the handlebars are a little bit higher up because I found I was getting a lot of issues with my neck and shoulders. For my outdoor bike because that's more geared towards racing and shorter distances and obviously being aerodynamic, I haven't got the same riser stem in. So, for the moment I've just got it as it comes out of the box, so to speak. So, the only adaptation really is the saddle that's obviously very specific to me.

***Interviewer*** 13:27
How did you come on that saddle? Was that a process of trial and error? Did you have others before that you didn't like?

***P15*** 13:34
I tried a couple of different saddles and then a very good friend of mine in the US races with the team, indoor track and everything else, and I went to visit her and she had this saddle and we went out for a ride and it was like that may actually be the most comfortable saddle I've ever sat on, which is, I mean, you know, it's always still relative I find with race saddles like they're not, you know, I'd rather sit on the sofa, sort of thing, but yeah. And it's uh, it's made by a physiotherapist based in California, and he's done a lot of research into sort of, you know, the best shapes and things for saddles. So, it's not a cheap piece of kit, but it's definitely helped in terms of me being able to ride longer distances.

***Interviewer*** 14:26
In terms of cycling, overuse injuries you mentioned you'd put an increase the height of your handlebars for shoulder and neck injuries, have you had any other ones like knee pain, hand or finger numbness, foot pain, lower back pain?

***P15*** 14:39
I used to have a real issue with my toes going numb in the shoes. So, I've experimented a lot with where the cleat sits on the shoe and just making sure, so that fingers crossed seems to have been sorted now, but yeah, predominantly it's neck and shoulders from just holding that sort of more aggressive aero position.

***Interviewer*** 15:02
In terms of, obviously you mentioned on, you don't have high handlebars on your race bike. Is it for kind of I supposed to Sprint length triathlon is it well comfortable enough? It's a relative term sitting in aero position. Is it comfortable enough that it's manageable for the kind of 20K? Or do you think you could make it?

***P15*** 15:21
It's comfortable enough, but I've also with my personal trainer been putting a lot of time and effort into sort of core strength and strengthening those muscles and being able to hold the position because I was finding that I was putting a lot of weight through my arms and then obviously it's, you know, it's putting all the stresses there. So, it's sort of a combination of, it's comfortable enough for the distance, but I'm also trying to work on building the muscle to be able to hold that position for longer.

***Interviewer*** 15:49
OK. In terms you mentioned that your new, current saddle is allowed you to ride longer distances. Is that one of the triggers that may be cause you to get uncomfortable in the saddle length of time on the bike? Is it more aggressive I suppose aero position? Are there any factors that yeah?

***P15*** 16:08
I mean it's definitely those two things is sort of saddle comfort, and you know I do tend to move around on the saddle as I'm riding and at some point you get to the point where it's like I haven't found a position that I haven't sat on yet, so, we're done. So, it's that combination of sort of neck fatigue and at some point, the saddle just becomes uncomfortable irrespective of how good it is.
And then I need to take a break.

***Interviewer*** 16:37
In terms of suffering from saddle sores, is it just kind of pain, discomfort or do you get any chafing or little spots or cysts or anything like that?

***P15*** 16:49
No, it's predominantly sort of it, it goes numb or it starts to ache a little bit, but I don't really have any issues with saddle sores as such.

***Interviewer*** 16:59
And where is it that gets? So, is it like sit bones? Is it more kind of upper thigh? Outer labia.

***P15*** 17:08
It's usually sit bones.

***Interviewer*** 17:10
In terms of you said, if it starts to get sort and you need to take a break is if you've is that in terms of kind of length of ride you would then kind of try and cut it short after that point or would you take a few days off the bike?

***P15*** 17:27
No, it's more that I try and break up the ride so I will, depending on the length of the ride, so sometimes just stopping at a traffic light is enough to sort of like get the blood flowing back and then continue on. Or sometimes, if I'm planning a longer ride then I'll take half an hour, sit in the sunshine, and then continue the ride. So, I don't think I've ever cut a ride short because of it. It's just then trying to find a way just to not sit on the saddle for, you know, 5-10 minutes and then be able to go on again.

***Interviewer*** 18:01
In terms of your longer rides, how long would these be like in terms of hours?

***P15*** 18:08
I guess the longest rides I do tend to be somewhere around the 80 kilometre mark, 60 to 80 kilometres, usually on a week to week basis I'm more doing sort of 30-40 kilometres, because obviously I'm targeting 20K race, but you know if the weather's nice, I'll go out for 80 kilometres, but when I, if I'm planning a ride that long I will plan breaks in for comfort.

***Interviewer*** 18:24
Do you ever have a problem when you're training indoors on Zwift or anything with discomfort or are the sessions short enough not to cause a problem?

***P15*** 18:44
No, we did well, I say we the team definitely complains. So, 25 to 30 K races, which are about an hour, is doable. Anything over that starts to get uncomfortable. Just because you don't have the same level of movement, obviously the bikes more fixed, so it's harder to shift around. You don't have a traffic light or a reason to stop or something else, so you know you're sort of sat and then you're sitting. So, anything over about 30 K, collectively the team starts to complain that it's uncomfortable.

***Interviewer*** 19:19
In terms of kind of saddle discomfort, does this affect your enjoyment of cycling or wanting to go out and ride your bike? Or do you just kind of just get used to it?

***P15*** 19:33
I think the adjustments I've made in terms of the saddle that I use now have definitely made me ride more. I think an element of it is just as with anything, it's endurance, it's training your body to get used to it. So, it's definitely held me back in the past, but I'm again, I feel like I have done enough that I am now willing to accept a certain level of discomfort to get the longer rides in.

***Interviewer*** 20:03
In terms of when you're racing, you said you race on Zwift or triathlon, is it for that length of time is it kind of manageable that it doesn't really affect your performance?

***P15*** 20:14
Yeah, because I tend to do shorter races, so then it's perfectly manageable, yeah.

***Interviewer*** 20:19
OK. That's all my questions on kind of bike set up. Is there anything else you'd like to add or any other issues you've had that have not asked you about?

***P15*** 20:33
No, I don't think so.

***Interviewer*** 20:35
Then final question, are there any areas you would like researching in regard to female cycling, so things such as the effect of the menstrual cycle, female specific training, bike set up, saddle choice or injuries or anything else?

***P15*** 20:53
I mean, I think the cycling world has come a long way in terms of being more female specific in terms of, you know, bikes and races and things like that. Realistically, because I don't work with a coach, I don't know how much. Well, no, actually, let me let me turn that around. It would be interesting to understand more in terms of how much I should or could improve my training by adapting to where I am in my menstrual cycle, because at the moment it's just sort of, it happens and I crack on with the training that I had planned and try and make the best out of it. It would be really interesting to understand if I could get more out of it by adapting my training plan to where I am in my menstrual cycle.

***Interviewer*** 21:39
OK, great. Is there anything else you think we need to do research in, around female cycling or is that the main area?

***P15*** 21:49
I think that's probably the main area.

***Interviewer*** 21:53
OK, great. That's all my questions. Is there anything else you'd like to add before I stop the recording?

***P15*** 21:58
Nope, I think that covers everything.

**P16 – interview transcript**

***Interviewer*** 0:05
So just to start with, a bit background about you in cycling, how long have you been cycling and what events are you currently competing in?

***P16*** 0:18
So I started when I was about 8 years old, just joined a local club and started doing cyclocross races, road races. Then moved on to the track at 10 years old. Became a sprint track cyclist, probably as second year junior and that's what I've probably done, yeah, that's what I've done for the since I was a junior, basically, so track sprints cycling on the velodrome. Very short, explosive, powerful efforts…...

***Interviewer*** 1:08
Obviously I know you've mentioned you've been having exams, but how's your training going at the minute and what competitions have you got coming up?

***P16*** 1:19
Yeah, it's going well. So, I'm currently doing three track sessions a week and two gym sessions. So, the track sessions are obviously your speed and power-based stuff, and then your gym is your strength, not like too sure yet when all my competitions are but there's definitely going to be a couple over the summer. So yeah, just waiting for dates for those.

***Interviewer*** 1:40
OK and moving on to your menstrual cycle. So, thinking back to when you started getting your period, how old were you at the time and what was it like for if you were playing sport?

***P16*** 1:54
So I was 13 years old when I first got mine, I think. I say to start with it didn't really have much of an impact. And then I think as the training stepped up, the intensity stepped up, the volume stepped up, then it became a little bit more challenging at times.

***Interviewer*** 2:16
And at that time did you get any advice or information around periods just in general, kind of school stuff or anything related to sport?

***P16*** 2:28
I think in school we had a little bit of education, but nothing was focused towards sport and then it probably wasn't until, probably quite recently actually, that I have had sport related menstrual cycle education.

***Interviewer*** 2:46

And when you said obviously once the training stepped up it became, your period became a bit more of an issue. What sort of symptoms did you experience?

***P16*** 2:55
I think just when I well, when I kind of the training stepped up, obviously, I was road cycling at the time to kind of going out on the bike for longer hours. You know if you’ve got your cramps and you're not feeling, you know, fully energized and then you know, needing the toilet more frequently, just little things like that.

***Interviewer*** 3:11
When you, obviously I think you've shared you on the contraceptive pill now, but before you were on that, did you ever think you had like heavy periods? Was that, or all bad symptoms? Was that anything related to?

***P16*** 3:29
Probably say I had an average bleed, but I did used to suffer with cramps quite bad and so quite painful and kind of aggy at times which that didn't disrupt the training but you know, you could feel it when you were training, which wasn't a nice feeling.

***Interviewer*** 3:42
Have you ever experienced any kind of mood related symptoms as well? Like kind of irritability, or has that not being an issue?

***P16*** 3:56
I think sometimes, like, tiredness and engagement. I think when I lack energy, I sometimes switch off a little bit and perhaps not fully focused in training compared to normal.

***Interviewer*** 4:00
Obviously you now take the contraceptive pill, do you experience withdraw bleeds on these?

***P16*** 4:18
Only in my break. Not obviously, yeah.

***Interviewer*** 4:22
Does that bother you in terms of training and competition or is it quite light?

***P16*** 4:29
It's quite light compared to what I used to experience, so I find it better for training and competition.

***Interviewer*** 4:34
In terms of decision to go on the pill, was it around birth control, managing symptoms or another reason?

***P16*** 4:43
It was more to start with to manage my period, so if I had a competition, like a major competition that I didn't want effecting, I could control that period when I had it.

***Interviewer*** 4:46
Would you just run the pill back-to-back?

***P16*** 4:56
Yeah.

***Interviewer*** 4:58
When you made the decision to go on the pill were you given, I suppose, choices of different hormonal contraceptive options or is this what was suggested by the GP?

***P16*** 5:10
So I went to the GP, I was given a few options. I was advised that the pill would be most suitable for me and at the time I knew a lot of girls that were also competing on it. So, I thought that was probably the most suitable option.

***Interviewer*** 5:24
Do you get any symptoms on the, when you're on the pill or have they kind of they settled down once you've started taking, once you've been on the pill?

***P16*** 5:38
First day or two, I still get cramps. Not bad as they used to be, but then after that it it's pretty fine then. Yeah, nothing too bad.

***Interviewer*** 5:46
Would you take painkillers for the cramps on the first couple of days or?

***P16*** 5:53
I normally would, yeah, the day before and the day of it starting, yeah.

***Interviewer*** 5:56
Yeah, I suppose you can, because you're on the pill, you know, it's coming. So, you can kind of pre-empt.

***P16*** 5:59
Yeah.

***Interviewer*** 6:02
And on those first two days, if you were competing, is that ever an issue or do you just take some painkillers and get on with it?

***P16*** 6:09
Because it's a lot lighter, I kind of just tend to get on with it. Normally the painkillers just kind of ease it, and then when I'm competing, I kind of forget about it anyway.

***Interviewer*** 6:18
In terms of training, do you make any differences to your training, I know you've mentioned you've got a coach, but do you or your coach make any changes around those first few days or would you just kind of run through as normal?

***P16*** 6:32
So most of the time I crack on as normal and there has been times where, say, the volume in the session might have to be slightly changed, but that's not a regular occurrence. I'd say I can pretty much just crack on as normal.

***Interviewer*** 6:43
You mentioned that you've quite recently had some advice around menstrual cycle and sport and what was that, who was it from?

***P16*** 6:57
Yeah. So, I'm at University, so as part of one of my modules. A physiologist from Sport Wales came into the session with us. And then I also had another one with her as part of, like, a more sport related one. So, I've had a couple of sessions with her.

***Interviewer*** 7:15
Have you found that helpful in terms of, yeah, anything to do, have you made any changes based on that kind of device or is it just kind of nice to know?

***P16*** 7:26
I think it was more just like understanding, you know, not why it's happening, but she kind of went through, you know, if you run the contraceptive pill, this is what happens and that's not something that I've actually seen before. So, it was more just like the education side.

***Interviewer*** 7:42
In terms of you mentioned that sometimes a very occasionally you maybe reduce volume in a session are you comfortable talking about the menstrual cycle with your coach or with any other support staff I suppose that kind of Welsh Cycling?

***P16*** 7:57
Yeah, that wouldn't. Yeah, wouldn't bother me mentioning it.

***Interviewer*** 8:00
Obviously over the last few years, there's been I suppose more discussion in the media around periods, menstrual cycles so some big athletes have spoken up like Dina Asher-Smith, Eilish McColgan. Are you aware of, I suppose the kind of media that made you maybe reflect on your menstrual cycle, seek support or anything like that?

***P16*** 8:23
Yeah, I have seen a lot more recently on social media about the menstrual cycle and, you know, being open about it and it's not something to be secretive about. And yeah, I've definitely seen a lot. I think it's encouraging as well for if you did want to speak up about it that you know, you know, it's OK to do that.

***Interviewer*** 8:40
And obviously, I think you mentioned you've got a female coach, does that make a difference in terms of comfort, speaking to a female coach over a male coach or is it this was not really been an issue because you've had a female coach for quite a while now?

***P16*** 8:48
Yeah, I think because I'm so comfortable with my coach now, I wouldn't second guess ever mentioning anything. But I think if it was someone more new or maybe a male, I'm yeah, I'm. I'm not sure if I would bring it up initially. But, yeah, I haven't really experienced that because I've had my coach for so long now.

***Interviewer*** 9:13
That's all my questions around kind of menstrual cycle is there anything you think I've missed or should have asked you about or you would like to add?

***P16*** 9:30
No, I think that's good.

***Interviewer*** 9:32
In terms of moving on to bikes and overuse injuries, and I think from your questionnaire you have obviously a track bike and a road bike, but neither of these are women's specific, is that right?

***P16*** 10:01
So the track frame itself is not women’s specific, but then I think the saddle I have is. But the frame itself is just a generic frame that yeah, males and females would use.

***Interviewer*** 10:06
Yeah, and I suppose track slightly different because you often build up the frame. Have you adapted it, I am thinking of things like narrow handlebars, shorter cranks. I know some of these now relate to performance, not just for women, but have you made any changes or same to your road bike?

***P16*** 10:24
So for comfort and aerodynamics on the track bike, I've got narrower bars now and a longer stem, just so I've got a bit more space. My road bike is probably a similar position as well.

***Interviewer*** 10:29
Have you suffered from any cycling overuse injuries? So, thinking lower back pain, knee pain, hand or finger numbness, foot pain.

***P16*** 10:51
I have had back pain previously, but that was more gym-based not from the bike. No, I don't think I've suffered with any overuse injury on the bike.

***Interviewer*** 11:02
So in terms of like lower back pain and the gym, was that just a case in modifying gym-based training? You didn't make any changes to the bike.

***P16*** 11:11
The only thing we change in the bike was my standing start session just because they are very, yeah, they target your back a lot. So that was reduced slightly whilst I had the back injury but it was mainly gym focused.

***Interviewer*** 11:24
In terms of saddle, you mentioned you have a women's specific saddle on your track bike. Do you also have a women's specific saddle on your road bike?

***P16*** 11:38
So on my road bike I've got an Adamo saddle, which would typically made for women. I know some men do use them, but yeah, they were typically aimed at women.

***Interviewer*** 11:48
Do you ride the same on both bikes, or because the sprint track is such shorter efforts do you ride the different saddle?

***P16*** 11:55
So because the track you get into a lot more of an aero aggressive position, I have got a different saddle just so I can kind of come slightly more forward on saddle, whereas the one I've got on my road bike is more for comfort. So, I could sit on it for an hour, two hours without pain.

***Interviewer*** 12:10
You mentioned obviously on the track that you're in a really aggressive aero position. Have you put a tilt on your saddle to help with that?

***P16*** 12:18
So my saddle isn't tilted, but I do naturally tilt to the left and just because of the shape of the banking, but no, the saddle is flat.

***Interviewer*** 12:28
Have you ever suffered from saddle sores?

***P16*** 12:32
Yes. So, I have in the past, especially during my endurance days when I was doing a lot more hours on the bike.

***Interviewer*** 12:38
So in terms of what factors, was it just time on the bike that affected that, you getting a saddle sore?

***P16*** 12:46
Yeah, I think it was time on the bike and I think it was perhaps maybe bike position positioning as well because I'd say now as I'm older, there's been a lot more put into you know, the position on the bike and not as such a bike fit. But you know, looking into that aerodynamics position, whereas before you, kind of just hopped on the bike perhaps wasn't fully set up for you, maybe you know needed a few slight changes.

***Interviewer*** 13:06
Do you ever suffer from any problems on the track because obviously you put huge forces through the saddle at speeds you're going at as a sprinter?

***P16*** 13:22
Yeah. So sometimes I'll often get like a little bit of soreness. Not necessarily a saddle sore. Because we're not doing as much volume on the bike, but you can sometimes feel after a session with a lot of efforts where you've really kind of dug deep a little bit of kind of soreness or tenderness.

***Interviewer*** 13:43
Is just more like pain? Is it like, a bit of kind of bruising from the pressure or?

***P16*** 13:49
Yeah, a bit of, not discomfort, but you can see where almost like the skin has like chaffed, like chaffed a bit and yeah, a little bit of irritation, but not necessarily like a saddle sore.

***Interviewer*** 13:55
Where is this? Is this sit bones, upper thigh, outer labia?

***P16*** 14:07
Sit bones, yeah.

***Interviewer*** 14.08

OK, so from pushing back into the saddle. And do you ever have any problems on the road bike when you go out for like an hour or two now?

***P16*** 14:19
No. I tend to be fine now. I don't do as much on the road anymore compared to what I used to do.

***Interviewer*** 14:23
In terms of if you've ever had a saddle sore, have you ever had to do any treatment, or have you just taken time off the bike or have you just kind of riding through it or had to use painkillers or repair creams or anything like that?

***P16*** 14:41
So for most of them I've just kind of ridden through it and maybe sometimes had to miss a session or two. Once I had to go to the GP just because it was quite painful and to see if they could give me anything for it and I was given antibiotics.

***Interviewer*** 14:55
So did it get infected?

***P16*** 15:01
I didn't think it was like infected because you couldn't see on the surface, but they basically said they weren't sure if underneath had got infected. And so, I was given those to clear it and it did clear it to be fair.

***Interviewer*** 15:11
In terms of, now I suppose if you don't suffer anywhere near as badly on the track, do you ever modify it with those lots of efforts or you just take as sometimes you have to do a big session where you might be a bit sore afterwards.

***P16*** 15:29
Yeah, I wouldn't say I'm modify it. Say I've got a bigger session than usual. You will feel sometimes a bit sore afterwards, but it's something that kind of just kind of will go down overnight or the next day. Nothing, you know, major, that requires me to see a doctor or miss a session.

***Interviewer*** 15:50
Do you anything to help with that? Like you know, sprinters often like to wear double shorts on the track. Do you ever do anything like that?

***P16*** 15:58
I only wear one pair of shorts, but I've got my preferred pair of shorts, so some are more comfortable than others and the chammie is better quality on some of them.

***Interviewer*** 16:05

Because that was what I was going to ask if you do anything else for prevent it. So yeah, wearing different shorts, do you able to control that in terms of Team kit or Welsh kit is?

***P16*** 16:23
Yes, well, are Welsh kit is pretty good quality to be honest. I think that was something that they made sure that it had a good chammie in for us and then obviously as long as we've got the jersey on, we can kind of if we find another brand works better for us, you can just buy black shorts in a different brand, yeah, they're pretty flexible with, yeah, comfort on the bike.

***Interviewer*** 16:29
Have you ever had to discuss saddle sores with your coach, I suppose either to modify a session or have an extra rest day?

***P16*** 16:53
Yes, there has been times in the past where I felt perhaps a bit sore, so we've had to slightly modify a session or maybe do an off bike warm up to reduce the time on the bike and just to yeah, basically stop it flaring up more.

***Interviewer*** 17:03
Would you say it has a much effect on your ability to train and compete? Or was it fairly well managed?

***P16*** 17:16
I'd say it's fairly well managed. I don't get them often. And I'd say when I do there not like you, you can still ride with them and it might be a little bit uncomfortable or not pleasant, but I've had nothing that, you know, I thought I can't race today because I've got a saddle sore.

***Interviewer*** 17:26
That's all my questions on that as again is there anything you think we should ask you about or you'd like to add?

***P16*** 17:47
I think, yeah, I think you have covered everything.

***Interviewer*** 17:51
Final question. Are there any areas you like us to do research on regards to female cycling? So, for example, menstrual cycle, female specific training, bike setup, saddle choice or injuries.

***P16*** 18:08
I think there probably is research out there, but I don't think there's enough on female specific saddles and positions because I know when I wanted to change a saddle recently there was a couple of articles and videos online, but there was nothing with clear research which showed what saddle style is best for women. It kind of said that it was all very, you know, you just need to kind of sit on it and try it. It's very broad. And so, I just thought that could maybe be narrowed down, but I know it's very specific. Yeah, that would probably be my only question.

***Interviewer*** 18:50
OK. That's great. That's all my questions. So, if there's nothing else, you'd like to add, I will stop the recording.

**P17 – interview transcript**

***Interviewer*** 0:03
So just to start with a bit of background, what events do you currently compete in and how long have you been, I suppose cycling?

***P17*** 0:14
I complete predominantly long course triathlon. And I've been, I'd say cycling properly since about 2020.

***Interviewer*** 0:26
And how's your season gone this year?

***P17*** 0:29
Good, good., yeah, really good season actually. Yeah, I've had really good results across the board, I think.

***Interviewer*** 0:39
So moving on to your menstrual cycle. So, thinking back to when you started getting your period, how old were you? What was it like for you at the time?

***P17*** 0:50
So I think I was 16. And I only had a couple. Don't remember it being that traumatic to be fair, but I remember the pain it came with. Then they all of a sudden stopped and I never gave it anymore thought. And I just thought, oh, well, I'm lucky because everyone else is in pain all the time, but I don't have anymore. And then they stopped until I was 25.

***Interviewer*** 1:19
Do you know what caused that? Or was it just unexplained?

***P17*** 1:24
It was a weight thing, and I've got polycystic ovaries but I didn't know that at the time. I was chubbier when I had periods and when I wasn't chubby anymore the periods stopped.

***Interviewer*** 1:39
OK. And when they started again at 25, had you put in a little bit of weight? Do you think that is what brought them back?

***P17*** 1:49
I think it was stress. I think that's the only thing I can attribute to, so I got told in the January, I couldn't have children because I didn't have periods. I think didn't realize I would be bothered but was obviously bothered. Had a big period and then got pregnant the next month.

***Interviewer*** 2:11
Wow.

***P17*** 2:13
I am a tributing that one period to being like stressed and like worked up. And then having kids - normal periods.

***Interviewer*** 2:23
And I suppose if you only had a few when you're younger, that didn't really affect you doing any sport at the time, did it?

***P17*** 2:31
No.

***Interviewer*** 2:32
Were you sporty when you were younger?

***P17*** 2:35
Not really, it was more casual - go in the gym, going for a jog, yeah.

***Interviewer*** 2:40
And when you were younger, did you get any advice around periods at the time? Was it just standard school chat?

***P17*** 2:47
Yeah, just the, when you about nine when they put the tampon in the wine glass in front of you, that was about the extent of it really.

***Interviewer*** 2:55
I presume you sort some advice from the GP when they stopped did you at the time? Or were you just kind of not really bothered?

***P17*** 3:03
No. I can't remember ever noticing or. I never really thought.

***Interviewer*** 3:12
And you said since you've had your first child that your periods regular again, so do they, do you get any symptoms with those?

***P17*** 3:20
Yeah, all of them.

***Interviewer*** 3:22
Yeah. I noticed your form, like everything.

***P17*** 3:25
Every single symptom you can imagine, so I ended up getting the coil after a while. But yeah, the cramps, the going to loo, the headaches, the tiredness, the mood swings, like clockwork.

***Interviewer*** 3:41
Is that during a period or was that like kind of the premenstrual - a few days before?

***P17*** 3:46
Days before.

***Interviewer*** 3:47
Yes. So, is that when your symptoms are worse, would you say?

***P17*** 3:50
Yeah. And you know what? Every month, I never realized what's going on. I'm like what is getting into me. I should know by now.

***Interviewer*** 4:02
So does that affect you training at all or you just kind of crack on through it?

***P17*** 4:08
Yeah, yeah. So, I think for maybe the two days before my period and the first day of coming on, I'm useless in terms of energy, feel heavy and motivation is low. And then like the second day of being on, seems to becoming out of it.

***Interviewer*** 4:34
And do you have to take any pain killers for the pain? Is that just the first kind of day or day before?

***P17*** 4:37
Probably 3 days. Probably 2 days before and the first day, and then I'm generally alright.

***Interviewer*** 4:50
Do you get any mood related symptoms?

***[short break for childcare]***

***P17*** 5:07
Yeah, so, just the tiredness I think doesn't help and like the motivation.

***Interviewer*** 5:21
Do you have any periods during your menstrual cycle where you feel more energetic and more up for training or have you not really noticed?

***P17*** 5:28
Yeah, I think it's probably a week or so after I've finished my period. I feel lighter. No headaches. Just an all-round, nicer person.

***Interviewer*** 5:52
So in terms of being on your period, do you say you'd suffer heavy periods?

***P17*** 5.55

Yeah

***Interviewer*** 5:56

Is that a problem around training? Would you not go out on the road during that time, or do you not ride the road much anyway?

***P17*** 6:05
No, I would. It doesn't bother me so much in training because when I am doing a hard effort, I've noticed this from being on the turbo, I don't really bleed. It's like it stops, you know, like when you get in the bath it stops. But it has worried me during racing before because I have to wear a tampon and a pad normally. Not affected me, but it's definitely something I consider when I'm racing.

***Interviewer*** 6:39
Has it ever fallen on an Ironman because obviously that's a massive distance to race on?

***P17*** 6:47
Trying to think. I don't think of full ironman it has. A half Ironman I have been on my period and I've not used anything and been all right because like I said, it's the effort. But I still got my cramps and things like that as I'm going.

***Interviewer*** 7:04
Would you do anything differently if you're racing and it's on your period, or would you just take a few painkillers and get on with it?

***P17*** 7:11
Yeah, I don't do anything differently really.

***Interviewer*** 7:15
Yeah. And in terms of affecting kind of those that first day, couple of days before, do you ever talk about that with your coach? Did you ever plan that in or?

***P17*** 7:23
I don’t actually, no. My boyfriend thinks I should. But no, I don't ever tell him.

***Interviewer*** 7:33
Would you ever write a comment on training if you use Training Peaks or anything like that? Would you ever write a comment?

***P17*** 7:37
I do use Training but no, I never say anything.

***Interviewer*** 7:41
OK. Do you train with power? Have you noticed any effect on your power data or is it just how you feel? Or is your power data OK?

***P17*** 7:49
I don't think. I can't say I've looked to be honest. I definitely run slower. So, my power may well be, either my power is down, or the effort is higher I imagine. But I definitely know when I'm running on my period, I'm slower and what not.

***Interviewer*** 8:10
This is quite a specific question. Do you ever notice any changes in kind of your core strength, coordination, balance throughout your cycle at any points?

***P17*** 8:20
I can’t say I have ever noticed.

***Interviewer*** 8:21
No, that's fine. Some people do. Some people don't. In terms of kind of competing, would you make apart from you said you don't really make any changes. Would you plan any kind of racing schedule around that? I know you can't set races or do you just kind of do the ones you want and if it falls on your period, you just get on with it?

***P17*** 8:39
Yeah, I'm not very good at playing ahead, so there's been times I've done a race and come on during the race. I'm like, ah, that explains a lot. Like I say, I seem to forget every month.

***Interviewer*** 8:52
Because I think you said you Garmin reminds you. Is that right? You track it on your Garmin?

***P17*** 8:55
Yeah, it does. But sometimes it's out of sync, so it'll tell me like two days after I've started, your period's due. Like the 10 mile one we did, I felt awful, I didn't want to go, I was in a terrible mood and then it felt really, horribly hard. A lot harder than a normal 10. And then I went to loo to get my number off my suit, and I was like, oh, then.

***Interviewer*** 9:15
So would you say if you end up racing on a period does it negatively affect your performance then or is it just more like motivation?

***P17*** 9:29
Yeah, I think it does, yeah.

***Interviewer*** 9:30
It just all feels harder?

***P17*** 9:32
Yeah.

***Interviewer*** 9:34
Obviously you've mentioned you don't really discuss it with your coach. Have you ever had any advice around menstrual cycle and being a female athlete and maybe how to train or compete around it?

***P17*** 9:44
No, I can’t say I have. It’s not widely spoken about really is it?

***Interviewer*** 9:51
No, I don't think so. I think it's starting to change, but most people don't, particularly if you've got a male coach. In terms of kind of leading on from that, how comfortable are you having conversations about the menstrual cycle? Would you talk about with your teammates or anybody else?

***P17*** 10:09
Yeah, definitely the girls. But I don't know. I'm quite a quite solitary in terms of my training and things, so I don't usually discuss it with an awful lot of people, but not through not being comfortable. Just doesn't come up.

***Interviewer*** 10:29
And are you aware of more elite athletes discussing the effect of periods on kind of competing? So, there's been the likes of Dina Asher-Smith, Eilish McColgan? Are you aware of any of those conversations?

***P17*** 10:42
Not those in particular, but I've spoken to Ally Dixon about it. The runner. And she I remember she told me that she feels better once she comes on, like the first day, her period starts. She's worse before. She says, she just stays hydrated and carries on, yeah.

***Interviewer*** 11:01
That's all my questions around the menstrual cycle. Is there anything you think I should have asked you about or I've missed that you want to say?

***P17*** 11:16
I don't think so. I just, no, I don’t know, yeah.

***Interviewer*** 11:25
So moving on to kind of bike equipment, I think on your questionnaire you said you have a Planet X road bike that you hardly ever ride and then you've got your Liv TT bike and that they're both women specific. Is that right?

***P17*** 11:39

Yeah.

***Interviewer*** 11:40

Have you made any modifications to those to suit your set up? So, thinking about crank length stems, brake leavers, anything like that.

***P17*** 11:49
Yeah, a lot of stuff's changed on my TT bike, and both of them have got different saddles. My TT bike has got, you know, I don’t know what the names of all the bits are. I don't do it.

***Interviewer*** 12:03
Don't worry.

***P17*** 12:06
Like the flat handlebars, and the bit that me TT bars sit on.

***Interviewer*** 12:10
The riser block?

***P17*** 12:15
Yeah. My so that one's that way with the bars on top and then the one that's that way, that's changed. I've got different actual extensions. What else have I got? I'm sure the bit underneath my seat is modified because I'm more comfortable further forward and it was to bring my saddle. So, I don't know what it is, but I'm sure something's different on that because I'm more comfortable, right on the tip of my saddle.

***Interviewer*** 12:54
And to make those changes, did you have a bike fit with your coach, is that how you arrived at those changes or do them yourself?

***P17*** 13:02
No I did them myself for comfort. I have had a bike fit a long time ago. And then all the little modifications have just been gradual along the way.

***Interviewer*** 13:15
And have you ever suffered any overuse injuries: knee pain, back pain, hand or finger numbness, foot pain, anything like that, saddle sores?

***P17*** 13:27
Terrible saddle sores. All the time, do you?

***Interviewer*** 13:29
Yes, one of the banes in my life yeah.

***P17*** 13:31
Yeah, I'm terrible with them. I've had a few hip tears. 3 tears in my hips. Don't think about anything else from the bike, wouldn't say, no.

***Interviewer*** 13:48
So in terms of the hip tears, are they a bike related injury or are they a running related injury or you're not sure?

***P17*** 13:52
Both. I think it's an overused thing, but once you get one, you're more susceptible then.

***Interviewer*** 14:00
Have you changed your setup to try and avoid that or is it been more rehab?

***P17*** 14:06
Rehab, rehab. And a general awareness when it is starting to hurt. But it doesn't hurt at all while I'm cycling. It hurts as soon as I stop and try and get off the bike, I realise something's going on.

***Interviewer*** 14:26
Is there anything that makes that more likely? Is it time trial position or time length of time on the bike or you're not sure?

***P17*** 14:33
I couldn’t tell you, I'm not sure. I think it's overuse. So, if I up my training, yeah.

***Interviewer*** 14:42
I think in terms of your saddle you said you changed them and I think you've got the Adamo saddle on your bikes. How did you arrive at that saddle? Was it just trial and error or did you get some advice?

***P17*** 14:53
Yeah. So, before I was kinda into, not that I'm completely into kit now, but my friend's getting rid of one and I only had a dead thin, sort of like a stock, whatever comes with the bike on both of them and my god the pain. So, I just thought I would try his because it looked a bit more cushiony than mine because he worked for 10 years, it was fab, and I just bought 2. I've got the same I think on both. They're very similar anyway.

***Interviewer*** 15:23
Because I think from your questionnaire you spend most of your time in time trial position in terms of training on the bike, so more front loaded. Do you have a tilt on your saddle or is it flat?

***P17***
Completely flat.

***Interviewer***
Yeah. And in terms of kind of saddle sores, you said you suffer really badly from them. What makes that worse? Is it TT position, length of time or was it just?

***P17*** 15:48
Got no idea. Honestly, I've got no idea. It's constant.

***Interviewer*** 15:54
Was it bad during the Ironman? The recent Ironman, or do you not notice when racing?

***P17*** 16:00
I didn’t notice on the bike but I noticed as soon as I start running. But I use in the Ironman, actually use the removable chammie. Yeah, so it was godsend.

***Interviewer*** 16:13
I've never knew that was a thing, but that sounds brilliant for running.

***P17*** 16:17
I know. It was a Zwift. It was a Zwift event years ago. One of the fellows.

[break due to poor connection]

***Interviewer*** 16:39
Sorry, I think that, I'm not sure when mine cut out. So, you're just talking about your removable chamois from a Zwift event?

***P17*** 16:47
Yeah. So I saw the fella that was leading took a chammie out of his Tri suit like, oh, so that’s a good idea. So we got one from Ryzon for like the for six hour plus rides. They got gripers in. So put it in before the swim and then we wiped it out before the run, which is great because they are expensive tri-suits.

***Interviewer*** 17:10
In terms of where you suffer saddle sores, is it upper thigh, sit bones, or is it like outer labia, inner labia?

***P17*** 17:19
Mine are more, like the crease between my thighs, and my bits, so right in there. I used to before changing the saddle it felt like I'd like squashed the inside.

***Interviewer*** 17:26
So, it's like a bruising?

***P17*** 17:35
Yeah, but then since swapping saddle I don't get that now. Now it's more like sores on the outside.

***Interviewer*** 17:41
And is that just from chafing or?

***P17*** 17:43
I think so. Bloody hurt when you get back on, if you've got one and then you get back on your bike. Spicy, isn't it?

***Interviewer*** 17:53
Ah yeah, it’s not great. Do you use chammie cream or anything to help with saddle sores?

***P17*** 18:00
Never, no.

***Interviewer*** 18:01
And if you get one, if you've had some chafing. Do you do use anything to treat that? Do you use sudocrem or anything like that? Or do you just wait for it to heal?

***P17*** 18:11
No, if it's really bad I put sudocrem on before bed. But generally, no.

***Interviewer*** 18:18
Have you ever had to get any medical treatment for a saddle sores or have you just managed it yourself?

***P17*** 18:24
No, never had any.

***Interviewer*** 18:27
And I presume this is probably answer is yes to this. Does getting saddle sores affect your enjoyment of cycling?

***P17*** 18:33
Ah, yeah, yeah, definitely long rides.

***Interviewer*** 18:38
So, what would your long ride be? How long? Like hours wise, would you do for your training for an Ironman?

***P17*** 18:49
Well, what I perceived to be a long ride and good training is different to what I actually do. So, sort of five hours is a good training ride. Make a good few of them leading up or 6 hours, anything over sort of 80 mile, a few of them. But for this Ironman I did one. I did one long ride, and all the others were about two hours. I feel like when I'm on my bike once I'm getting towards like 2.5/3 hours, I'm waiting for everything to start hurting then.

***Interviewer*** 19:27
In terms of saddles sores, is it both sides or you like kind of one-sided imbalance?

***P17*** 19:33
No, I get them on both.

***Interviewer*** 19:34
And do you ever mentioned them to your coach if you need to adapt training around them, or do you just get on with it, whatever's planned?

***P17*** 19:41
Yeah, just do it.

***Interviewer*** 19:43
So you wouldn't take a few days off the bike if it was bad?

***P17*** 19:46
No, I wouldn't take a few days off the bike if my legs were coming off.

***Interviewer*** 19:53
So apart from like obviously changing saddle and you've got a removable chammie for when you do the long-distance triathlons, have you done anything else to try and help with saddle sores or reduce the incidence of getting them?

***P17*** 20:05
I bought suits and I've worn different suits so. I've worn the Nopinz speed suit before for both triathlon and duathlon because that's the comfiest thing. I don't get any sores in that at all. But it’s too tight to wear for an Ironman, so bought a different try suit like a long distance specific one.

***Interviewer*** 20:29
Yes.

***P17*** 20:33
Still garbage.

***Interviewer*** 20:35
Yes. Have you got anything else you'd like to add around kind of bike fit, saddle sores, that kind of overuse injury?

***P17*** 20:47
No, I don't think I'm particularly interesting candidate for you to be fair, but.

***Interviewer*** 20:55
No that’s fine.

***P17*** 20:58
The only thing that I notice is with the periods thing before, so I always argue that see 3-4 days a month maybe over a year. You're talking like over a month's worth of training that's inhibited by symptoms of your period. I just think us girls are all on the back foot from the start, aren't we? And I say I'm probably quite lucky with just 2-3 days. Other girls are missing, well, more than what I am. Well, certainly not maybe not missing the sessions, but the quality. So, you losing a month minimum, your quality's gone down, hasn't it?

***Interviewer*** 21:44
And my final question, are there any areas that like us researchers to do regarding kind of female cycling? So, thinking around menstrual cycle, female specific training, bike setup, saddle choice, injuries?

***P17*** 22:03
It'd be interesting to see the effect of heart rate. Your heart rate goes up when you're on your period. Be interesting to see what effect that has, or if any on your training long term. And so you it's not like you're doing heart rate like high intensity training and getting the gains from that. You're doing low intensity with a high output for such low returns. It would be interesting to see what effect it has, if any, if it has the same like residual effect as an extra months high intensity training, I don't know.

***Interviewer*** 22:44
Yeah. Is there anything else you think or is that the main one?

***P17*** 22:48
That’s it really. Are we being ripped off or are we getting bang for our buck during those?

***Interviewer*** 22:50
OK. Is there anything else you'd like to add before I turn the recording off?

***P17*** 23:01
No, no, thank you.

**P18 – interview transcript**

***Interviewer*** 0:05
So just before I start and bit of background about you and cycling, so how long have you been cycling? What events do you currently compete in?

***P18*** 0:15
So I've been racing for 25 years. So, I started back in 1999 doing mountain biking, and so I've seen the sport change an awful lot….. Had a couple of years out. Like I was done. Hating the sport. Got injured. I've come back into it, so, from 2012 onwards, so yeah, basically that and I do road and crit racing at the minute. Done a bit of cyclocross. So yeah, that's quick, that's the quick one.

***Interviewer*** 1:00
So how has this year gone? Obviously, before the recording you mentioned had COVID, but before that, how had your season gone?

***P18*** 1:08
Yeah, really good….

***Interviewer*** 2:17
So in terms of your season because it's getting towards the end of the road season, is that kind of you done for this year and then focus more back onto next year or you going to do any cross in the winter?

***P18*** 2:27
So I've got, we got a couple of UCI races out in Belgium, and we've got two National series left and I got a crit in Cambridge like just a, I think it's nat A, but it's like I've just done it because it’s local. I'm on the start. I'm on the first reserve for a 7 day stage race in the South of France in September called de l'Ardèche did it last year, so I'm probably going to alter my training towards that with heat adaptation in the next 5 weeks and be right with that. Yeah, not quite finished yet.

***Interviewer*** 2:58
So moving on to the menstrual cycle. So, thinking back to when you first started getting your period, what was it like for you? How old were you at the time?

***P18*** 3:07
I think I'll pull you around about 13. It was traumatic. I didn't like the fact that I was having a period like for me it was really big issue. Like I kind of I'm quite, I was tomboyish, grew up like in cycling with boys and it was horrific. I just hated it. I just wanted to ignore it. I couldn't deal with it very well and all of that. So yeah, that was it. I think it's about 13. And then when I was on the British cycling talent team, they recommended I went on to the pill, so that I could control it for national championships, which looking back I was like, you know that's not good. So, I was staying on the pill, so that it would, it would not interfere with my national champs on the track.

***Interviewer*** 3:53
In terms of going on the pill, was that would you run it back to back so you didn't get any period? Was that how what they told you to do?

***P18*** 4:00
Oh, I can't remember.

***Interviewer*** 4:01
Don’t worry if you can't remember. It's a long time ago.

***P18*** 4:06
Yeah, I think I still had a period. And then when I was 16, my mum had cancer, so they had to change the pill. So, I wasn't allowed a certain type of pill, so they've changed that. And then I was on that Cerelle whatever it's called, and I've been on that forever then until I was 30.

***Interviewer*** 4:19
So obviously you said in terms of when you playing sports was it affecting you? Was that why British Cycling advised as a quite young teenager to go on the pill?

***P18*** 4:35
Yeah, I think it was more. Yeah, it would have been. Yeah, I think it was more of a case of trying to stop it for certain events, controlling it like that, if that makes sense, that was that was literally the only reason. Obviously, I wasn't having sex or anything. So yeah.

***Interviewer*** 4:53
Before you went on the pill. Were you getting symptoms like cramps and things like that? Any mood related symptoms or do you not remember?

***P18*** 5:01
I remember just mood swings. I don't remember anything else, so I remember more of the being bitchy and horrible to my mum.

***Interviewer*** 5:10
Do you think it had any effect on your performance at the time?

***P18*** 5:14
I think it did purely because that's why I went on the pill. I think it I it must have done at that point, yeah.

***Interviewer*** 5:21
In terms of obviously going on the pill quite young, were you given any options, or were you just suggested the pill? Were you given any options about other any hormonal contraceptives? Was it informed choice or was it?

***P18*** 5:37
I don't so back. I've never. My mum would have known about the coil like that's what she used to have, but I think back then we're talking sort of 20 odd years ago. I don't think there was as much information out there as what there is now or as much choice. So, I think it was a case of it's this one or this one, let’s try this one and that's it. Like literally, I don't remember having much. Yeah, and it was the thing to do at the time, it was to go on the pill. Yeah.

***Interviewer*** 6:02
Did you get any symptoms from the pill when you first started? Like, often more mood related, things are often.

***P18*** 6:11
I honestly don't remember. I remember struggling with weight though. Always struggling with weight. But I my mom always said I was pretty moody, so judging from that I would suggest it would have been my moods. Yeah, would have been quite impactful if that makes sense.

***Interviewer*** 6:29
Obviously you said you changed from your about 16 because of one had a greater risk of cancer. Did that change anything in terms of how you responded on the pill or?

***P18*** 6:41
Not that I remember no.

***Interviewer*** 6:43
And have you always taken it where you have one week off, so you get a withdrawal bleed?

***P18*** 6.47

Yeah.

***Interviewer*** 6:43

And in terms of, obviously you said you got advice from British Cycling to go on the pill. Was that from the doctor at the time, or was that kind of more coaches just saying what your GP?

***P18*** 7:02
Yeah, more coaches go to the GP. Then the GP is like, yeah, OK, then that's a good idea. Yeah, sort of thing. Yeah, no questions like, yeah.

***Interviewer*** 7:10
And around that time, did you get any apart from maybe being advised on the pill? Did you get any advice for education around periods, menstrual cycle, either school related or anything to do with sport?

***P18*** 7:22
Sport wise, no, it definitely was not talked about within cycling with coaches, never tracked, never asked nothing. School wise, you go through the usual sex education and that was it, yeah.

***Interviewer*** 7:35
I don't see you said you came off the pill when you were 30. How has that affected kind of symptoms?

***P18*** 7:46
It’s changed my life. I feel like a completely different person. I feel that my brain works better, so I struggled with, so this is really I, and I'm sure it is contributed because I struggle through school. I couldn't concentrate. I hated school. It wasn't for me. At the age of 30 I came off the pill. I enrolled on a uni course. I've got a three year degree in sports therapy rehabilitation, right, so for me, there were a lot of things contributing to it. I really had to do a lot of learning on. I have moved like I feel the change each month within my body. I'm more attuned to it because I feel like I'm more natural. So yeah, it I personally would recommend anybody I know to come off the contraception pill. Like if they could, and I'm really lucky that I've been able to do that because I feel it's really changed my life. Yeah.

***Interviewer*** 8:44
In terms of obviously sounds like it's been a really positive experience, but in terms of do you ever get any negative symptoms around your period or that premenstrual phase or anything?

***P18*** 8:55
So I don't know why, so, one month I'm absolutely fine. I don't even feel like I'm coming into my period and like oh it started absolutely fine. No pain, no nothing. Then the second, like the month after I got sore breasts. I've got achiness. I'm a bit emotional. So, it's like one month, fine. And then one month, a little bit worse. It's like an alternative. Bizarrely, I don't know what that is. There are days where I would sit and cry, but I know why. I did a lot of research into the hormones and what's what and everything. So now I have an understanding. I really understand why, and I can deal with it if that makes sense. So yeah, it is what it is. Some months are worse than other months. I can certainly feel things changing. I can feel my hormones changing. I feel my body temperature changing. My heart rate increases, you know, all of that. I get all of it, but I don't get the so occasionally I might get the bad cramps, but it doesn't bother me because I know what it is if that makes sense, yeah.

***Interviewer*** 10:01
Do you ever have to take any painkillers for those or they're not that bad?

***P18*** 10:05
No.

***Interviewer*** 10:06
I think you mentioned on your questionnaire that round about I think, is it ovulation, you have noticed a slight drop in power. Is that just for like a day or two around that ovulation?

***P18*** 10:16
Yeah. Bizarrely, it's usually about 24 hours or like for. I know myself that if in my training, I track if I've got efforts that day I either say to my coach, can I just have endurance or I just suck it up and know that I'll lose 30 watts, like I can lose up to 30 watts and it's really it took me years of like this one day, I'm like oh the world's falling apart like because I feel really emotional, so I tracked back and interestingly through my Training Peaks on pretty much around about same time, bang on the month, there'll be a negative comment in my Training Peaks. The only negative comment I'll ever have will be on and it's like oh, that's weird. That's on that day, huh? That's on that day. And there's a real, I can look back three years and I can see that cycle. So, I'm like oh you know? So, I plan around it, yeah.

***Interviewer*** 11:07
OK. Are you quite comfortable talking with your coach about that to plan around it?

***P18*** 11:12
Yeah, he's really good. He's coached me for the last of 4-5 years now and he's yeah, he's super like he'll see within my the way that I comment in Training Peaks he'll know. Like if I'm if I'm sort of like about to start with period or something like that and he understands he's coached lots of women. So, we're pretty open.

***Interviewer*** 11:32
In terms of do you ever make any other kind of changes in terms of training or competition around your menstrual cycle?

***P18*** 11:40
No, because I refuse to change my training. I really dislike these people on social media that say you need to change your training in and around your period, your menstrual cycle. I don't agree with that. I feel that if it makes you feel good, go and do it and I feel yeah. So, I don't I don't change any training. I'll be aware of that day. I know for a fact that as soon as I start my period that I can push myself harder. So, I'm like, yes, let's race, you know, if it falls in within race day, fabulous. I know like in my head I'm kind of like my pain thresholds better. I'm just going to go like I'm going to have a good day. Kind of thing. So, I trying to spin it on a positive way. If it falls on that bad day, I'll deal with it and that's that. I understand why if that makes sense, but I've never changed racing. I won't change training. The only thing I'll probably alter is my diet and that's it.

***Interviewer*** 12:32
Yes. So how would you alter your diet then?

***P18*** 12:35
So we'll probably increase my carbs and I'll probably increase like I do increase my sort of fatty fats sort of thing, like my fishes and stuff like that. You know, I just alter it slightly and if I do crave something then I'll eat it.

***Interviewer*** 12:54
So this is around you period itself. You'll increase your carbs and kind of.

***P18*** 12.55

Yeah.

***Interviewer*** 12:56

In terms of would you say you get heavy periods, does this affect you if you go out for, I think you mentioned you got to six hours on the bike, do you ever have to plan around that?

***P18*** 13:06
No. I use a menstrual cup which is an absolute game changer. So, as I'm concerned that is the best thing since sliced bread. So, I don't. I'm fine. Yeah.

***Interviewer*** 13:17
This is quite specific question, but do you ever feel that you get any changes in kind of core strength or coordination or flexibility around your menstrual cycle?

***P18*** 13:27
No, and I think purely that is because of the sport that I do. I think if I was doing like a ball sport or like a different sport, I may well find that, like football for example, I know that the studies out there that people find the difference, but as a cyclist, not really my brain's OK, you know, I mean I don't get brain fog or anything like that. So yeah, no.

***Interviewer*** 13:48
I think you've mentioned you track your cycle. Is it the Flo app you use?

***P18*** 13:52
Yeah.

***Interviewer*** 13:53
So do you use that just to track the phases or do you put symptoms or anything else in there or just stick them on Training Peaks if you feel a bit rubbish?

***P18*** 13:59
Yeah, I don't pay for Flo. So, I all I have, all I'm able to do is track. Then in Training Peaks if I feel my period has affected my training, I put it in. But other than that, I'm not bothered like I know. I know what's happening, you know.

***Interviewer*** 14:17
Obviously you mentioned that when you're younger on the British Cycling Programme you didn't get any advice, have you had since had any education or advice around menstrual cycle in sport or have you done some research yourself?

***P18*** 14:28
I think because I did my degree at 30, we did, there's like a little bit of a module in there and I coach as well. So, I feel I took the responsibility to find out for myself and I you know, I can navigate Google Scholar kind of thing, you know, and see things like that. So yeah, I think I certainly invested a bit of time into that because obviously I'm female. I needed to because I wasn't on any contraception and felt that my cycles were natural and I needed to understand why I was feeling the way that it was.

***Interviewer*** 14:58
Have you ever had anything through any of the teams you've been with? Have they ever gave any education? Do they ever, I suppose, ask about it, monitor it in terms of for health reasons?

***P18*** 15:13
So no, but the team, so the team I'm on at the minute, I help run with my partner and I, the other year, a female nutritionist got in touch, she was really big in advocating eating around the menstrual cycle. So, we have had certain zoom calls with this nutritionist who has gone like try and eat this. This is why, this is this. She was really big into the energy availability and stuff and RED-S. We therefore then had another nutritionist work with us this year, George from British Cycling and British triathlon, who again will be like right, energy availability, RED-S, these are the signs. So, we do and we have access for the riders if they feel they need to if that makes sense. But it's also within the team it's down to the coaches as well, which are individual to the team. But because I really think it's important I try my best to bring people in to talk to the girls or they ask me questions if that makes sense, yeah.

***Interviewer*** 16:12
I think you've talked that you're pretty comfortable about having conversations with this with your coach, I presume with your teammates as well, try and encourage open conversations around this topic. Are you aware I suppose over the last couple of years there's been more elite athletes talking about the effect of periods, maybe on their performance of like Dina Asher-Smith, Eilish McColgan has that had any effect on you maybe doing more research into the area, being more aware of it?

***P18*** 16:44
I feel that I started looking into this a little bit before it became so online. I also have had experience of a coached rider who got RED-S with the previous coach, so I've for the last two years I've had to learn how to how to support her and change her life basically because she was pretty poorly. Because I've seen the dangers of it. The mental side of it all, you know how it impacts people, I am very open like, and I will call out anybody that says anything else. So, what are my coached riders went to the doctor's two weeks ago because her pill, she was bleeding, she's missed a period or something like that. And the doctors like, yeah, don't worry it's normal for athletes to miss your period. I hit the fuckin roof. Excuse my language.

***Interviewer*** 17:36
Oh no, I don't blame you.

***P18*** 17:36
I was just like this is not acceptable and I, she's like, alright, I didn't realize. I'm like, you know, he's. Oh. Anyway, so doctors as well are just as bad. Like oh, it's normal for an athlete to lose their period. I'm like it is not normal. No way. So yeah. I'm, yeah, I'm really passionate. Sorry. I'm just like.

***Interviewer*** 17:54
Oh no, I'm with you on this because yes, the information that comes out of doctors is sometimes yeah, because I used to be a runner and obviously RED-S is massive in runners.

***P18*** 18:00
Yeah, it is even now. So, I know that you see people filtering out on social media about it. It's still rife, like it's still there and then I know if you riders might I coach are still a bit like, no, no, it's OK. It's OK. I'm like when was your last period? Oh well, you know, when I had to really force it out some riders so yeah.

***Interviewer*** 18:23
No, I always thought it got better, but it doesn't seem to be.

***P18*** 18:29
No. Uh yeah.

***Interviewer*** 18:32
I think that's all my questions around periods and the menstrual cycle. Is there anything else you think having asked you about you'd like to say or add?

***P18*** 18:43
No, I don't think so. I don't think so.

***Interviewer*** 18:47
OK. So, moving on to kind of bicycle equipment and setup. I think obviously you ride for teams, so you get a team bike and I think you mentioned on your questionnaire, you're not a fan of women's specific bikes. When you get a team bike, do you make any modifications to that like handlebars, stem, crank length, any of those?

***P18*** 19:07
So I have a very trusty bike fitter that I see twice a year, so every year I'll go down with my winter bike, change my winter bike because my body changes. A changed the saddle. I changed like, it depends on the, the bike does come as far as setup wise, i.e., when we order the bikes that manager goes what length stem do you want? And I'd be like, well, the bikes this big, it'd be like a 110.
So I have a set of, so yeah, it basically comes, I change the bars, I change the stem, I change the cranks. I really got a thing about women not riding 165 cranks. Change the saddle and then I have and we're all on the same pedals, but I always use the same pedals as well.

***Interviewer***	19:48
So if you don't ride 165 cranks, what do you ride instead?

***P18***	19:52
No, I do ride 165.

***Interviewer***	19:54
Ah, you do. OK. Yes.

***P18***	19:55
Yeah. Sorry, I do and I'm a big advocate of that as well for women, yeah.

***Interviewer***	19:58
You obviously been cycling for very long time. Have you ever suffered from any cycling overuse injuries so back pain, hand or finger numbness, knee pain, foot pain?

***P18***	20:08
So this year I've been diagnosed with spondylosis in the spine. So pretty much arthritis all the way through my spine and back, and the only time I'm not in pain is when I'm on the bike, but it it's kind of stemmed down to just degeneration and riding horses as a kid's kind of thing. So, no on the bike and I don't feel that that, yeah. So, no overuse stuff. I've just got that really poor spine, which I think is kind of natural my age and all of this sort of thing. And regards to injury, I've never had any injury specific to cycling unless I've crashed.

***Interviewer***	20:47
Yeah, well, that's really good.

***P18***	20:51
I do put that down to the fact I've been riding a long time. I don't just. I'm like, I just. I can't. Yeah.
So yeah, I just think I'm just used to riding that all the time and you get used to it if that makes sense.

***Interviewer***	21:03
What type of saddle do you use, and do you use the same one on all your bikes?

***P18***	21:11
Yeah. So, I did a lot of work with my bike fitter because I had issues with my saddle well for quite a while I went to a particular saddle and I was still quite unstable on the bike and I did a lot of chatting to my bike fitter and a lot of playing around with things and now for the last four years I've ridden a well, three or four years now, I've ridden an S-Works 3D printed mirror saddle, so I feel that technologies change an awful lot. So, I have, I use the same saddle now and it's, I put that on both bikes. So yeah, I did a lot of talking and adjusting and explaining to a male bike fitter and it's sorted now. But yeah, it I've gone through a little bit but not unbearable enough to not ride if that makes sense. So yeah.

***Interviewer***	22:05
When you said you felt unstable on the bike, was just that pelvis rocking or?

***P18***	22:09
Yeah. So, have you heard of Adamo saddles?

***Interviewer***	22:12
Yeah, yeah.

***P18***	22:12
So the two prong ones. So, I started writing them back in 2015 and that stopped my saddle sore, although it made me swell up on the one side which is never gone down, but I was comfy. I wasn't bleeding from any saddle sores stuff like that and but yeah, rocking because you just not stable on the bike and the way that the seat was like the saddle was designed was that I was not getting my weight over the bottom bracket in the right way. So, ended up riding this, so I used to rock like, so instead of sitting on side like that, I'd rock forward, so that it would keep my spine neutral, but now I'm sitting more upright, more comfortable on this S-Works, but these saddles have only just come out recently, in the last couple of years, so its technology changing if that makes sense as well, which has helped me then be comfortable on the bike if that makes sense.

***Interviewer***	23:05
Do you ever put a tilt on your saddle to kind of help with that, or do you have a flat saddle?

***P18***	23:09
It's pan flat, like this flat as you can.

***Interviewer***	23:12
I suppose probably if you were rocking too far forward, the last thing you want is a tilt to kind of tilt your pelvis.

***P18***	23:16
Yeah, yeah, yeah. So, I'm definitely more stable. I have more of a, I sit more on the saddle but the problem with women specific bikes and this is why conclude to is that for some reason they make the front ends too high. So, I prefer being, I've slammed my bike down, I prefer the aggressive male style, which means that I can sit how I want to sit on the saddle and be really low and aggressive, so I've had to adjust things because of women specific equipment which really annoys me. Like yeah, I can't get the position I want, but because of these bloody bikes. But yeah, anyway sorry, I rant. I'm really. I'm sorry, I do rattle along.

***Interviewer***	23:55
No, no, no, it's great. And you said you got swelling on one side was that like the outer labia on one side swelled up or?

***P18***	24:01
Yes. So, on the, it's the left-hand side, and that really stems from being made to ride saddles that didn't fit me or didn't work. So, for example, I had to, I wasn't allowed to change saddles, and I've, and there weren't that many saddles on the market for women back when I was racing as a junior. So, it stems from that, it's never gone away. It's no bother. It's slightly bigger than the other side, doesn't cause me any issue now because I have got a proper bike fitter that talks like listens to me with my weird requests, you know. So, it's taken a long time really to find the right equipment for me that fits, but it's now there and I'm super comfy if that makes sense. So, it's trial and error. It's having the money to buy different saddles and trying them and having people that listen, you know, I've never had that as a junior. We just, women, nobody cares, you know, just get on with it. You know that.

***Interviewer***	24:59
Yeah, and getting one sided issue did anybody suggest like an imbalance or twist on the saddle that may be cause one side.

***P18***	25:08
It was more to do with the fact that and. Well, no, we never, we never really got to that. It was just the fact that I was so uncomfortable on these saddles that I was sitting off and it just swelled up one day, got bigger and bigger it. It started to get really big, and I was like, I have to do something about this. And so that's when I had a radical change to the Adamo saddle, which did reduce it. But then I ended up having a lump down there, going to the doctor and they were like, oh, it's tendinopathy, no, it's a subcutaneous cyst. Went to go get it removed, the doctor said no, it's tendinopathy. So, I don't know what it was in the end, but it's gone but other than that, like, yeah, that. Yeah, I'm fine. Like yeah, it’s from past issues and I had to have a significant change to stop it from like being bad, if that makes sense, yeah.

***Interviewer***	25:59
Yet did you have to take any time off the bike during that period to let it go down, or did you just keep riding and it was just change in saddle.

***P18***	26:05
We’re bike riders, we keep going.

***Interviewer***	26:08
I know we do. So when you got medical attention for that was it just through the GP? It wasn't any specialists?

***P18***	26:16
Yeah, because I wasn't on any federation, I wasn't with GB then I was just racing for a team.

***Interviewer***	26:22
In terms of is that the main saddle issue you've had - kind of swelling, have you had any other stuff like spots or cysts or anywhere else, or pressure?

***P18***	26:30
No, because I I'm super. So again, I have this conversation with my coached riders when they're like oh I've got cyst down there. I've got spots. Well, what do you do? I shave it with a razor. Well, don't. Like use beard trimmers if you want to cut the hair underneath there. So, I know how to handle myself down there if that makes sense. I know if I shave, I will get lumps. I'll get spots, so I use beard trimmers and then if that's what I want to do. So, I'm aware of what I need to do so I've not had in a very long time any spots, any issues. I get out my chammie straight away. I get showered straight away. It's personal hygiene as well, like that helps, so yeah.

***Interviewer***	27:10
I presume your shorts, your kit and stuff is pretty good that doesn't cause any problems with seams or anything?

***P18***	27:18
No, I'm pretty robust with that sort of thing as well. Like in the last four years, I have not had one issue since I changed saddle and I've made the effort to really invest in a good bike fitter. I've never had any issues whatsoever.

***Interviewer***	27:33
I presume obviously quite open about other stuff, you are quite happy talking to your coach about this if anything happens and you had to take some time off or modify anything?

***P18***	27:41
Yes, I give him a warning. I'm like, are you ready for this? He's like, yeah, that's fine. I don’t go into too much detail, he doesn't need to know some of the details, but he does know. Like if I have an issue, I'll be like, I can't ride. I've got an issue, and he'll be like, yeah, cool. He understands, you know, he's bike racer as well, so yeah.

***Interviewer***	27:58
So I presume in terms of now, since you've managed 4 years without it, you kind of nothing really affects you getting a saddle sore if you're pretty good with all the kind of getting out your kit post ride and all that kind of thing. So I think that's all my questions in terms of saddle sores because you've kind of you've done most of the things that, yeah, maybe would suggest you do, and I don't think you don't ride TT or anything like that which?

***P18***	28:29
I try not to unless it's the stage race.

***Interviewer***	28:34
So is there anything else you'd like to add around that kind of bike set up, saddle sores?

***P18***	28:41
Yeah, I think. I think for some riders, so there aren't many female bike fitters right? So, I feel that when young riders, especially female riders, go to a bike fitter, they won't speak up and they say, well, he said that's right. And I'm like, well to me, it depends whether you got an inner or an outie you know theres so many factors that men will not understand, and I try encourage all my riders to be really open and honest. If it's not comfortable, you have to say so. I went through with my bike fitter. He's great. You know, I've. I've seen him twice a year for the last five years. Literally went in there and said right, I have this issue. I feel like this on the bike. One side is a little bit. I get sore if I do this or this that the other and I want to sit like this. OK, then explain this to me and I be like, well, if I sit like this, it squashes everything. So, you have like, so yeah, it's really interesting. I'm a really big advocate of men learning and understanding as well, but the sport is changing for the good and I feel that there are a couple of women fitters out and about. How good they are I don't know, but yeah, it's really interesting. But yeah, I’ll rattle on, but yeah, it's I try and get my riders just to speak up.

***Interviewer***	29:58
My final question, are there any areas would like researching in regard to female cycling? So female specific training, menstrual cycle, bike setup, injuries, addle choice.

***P18***	30:12
Sorry, say it again

***Interviewer***	30:13
So that any areas you'd like researching around female cycling that you think we should do some research into?

***P18***	30:13
I think yeah. So, I think how women sit on a saddle, like, literally like the different shapes of people because they're so it, like people are so different. It's not like a man sitting on what men sit on. There's so much there and I don't know how you would research something like that, but I really feel that there needs to be more research in the effects of women when they sit on everything because I've done races where I've just sat in a tuck position and I've gone completely numb down there, you know, and I know men do when they do TT’s and stuff like that, but I just feel that, yeah, I've not seen any research on. I mean, I'm really. I always have saddle mapping. For example, when I have my bike fit, so I see the pressure points and we just adjust but I don't think I've seen any research on how women sit on saddles. Maybe that's just me.

***Interviewer***	31:13
No there isn't.

***P18***	31:15
So, there you go. I think, I wouldn't know how you would study that, but yeah, I think that everybody's so different that it would be really hard to sort of study that but I do feel that, you know, I know. So, growing up, when I was on GB, I know a couple of high profile riders that have had to have surgery and had a designer vagina. You know they they've had bits adjusted down there so that it can ride like I'm talking like Olympians, you know. Should that be happening? I don't know.

***Interviewer***	31:43
No.

***P18***	31:43
Like you know, should it be happening? So yeah, I know those are riders that are needed surgery because of it. So yeah, and again menstrual cycle we just need to keep pushing that RED-S, low energy availability, everything, and I think that I just. I think when it comes to like contraception and hormone replacement and all these sorts of things, when you kind of look at the bones of it all, it's it was really haphazard I think when it first came on board and very little research into it, are we causing damage in the long run, you know, all these things; I just think that that messing with your hormones is not ideal. You know, the more research that comes out and the more you learn about it. And I'm like, oh. OK, then then like I've read a really good book. Ah man, I can't even know what's cool, but basically, she at the age of 30 came off the pill as well, and her life changed, you know, and I just feel people just get stuck into it because they feel that that's what they have to do. And I feel that lots more research needs to happen and a lot of research male all based, you know, when you look at any sport, any study, focus group and all that sort of thing. So yeah, we're getting there slowly, but I feel that that's really important.

***Interviewer***	33:02
OK, great. Is there anything else you'd like to add before I stop the recording?

***P18***	33:06
No, I feel like I've rattled on enough.

***Interviewer***	33:07
No, no, no, it's great.

**P19 – interview transcript**

***Interviewer***	0:12
Just to start with a bit of background about you and cycling. So, when did you start cycling? What events do you compete in?

***P19***	0:23
I guess I bought a bike 10 years ago after other sports and having niggling injuries and things, and started to cycle more because it was a little better.

***Interviewer***	0:36
What events? I think on your questionnaire, you said you're not competing obviously as much at the minute, but what events have you competed in in the past?

***P19***	0:47
In the past. Anything that's going on really. From cycle point of view, time trials probably where I started and got involved with crit racing, a bit of road racing. Actually, cyclocross racing was probably the first because I accidentally bought a cyclocross bike first instead of a road bike and thought we should use that for it's appropriate use.

***Interviewer***	1:13
Yeah, in terms of, like, I suppose, training at the minute, how's that going? Are you doing any competitions?

***P19***	1:20
I haven't got anything lined up specifically at the minute. I do enjoy like the team time trials and things which are more probably springtime starting again. I haven't got plans at the moment for cyclocross over the winter, but I know that one of my teammates was looking to have a go, and I might go along just to help out a little bit. Yeah, but I don't expect to be at the sharp end at the moment.

***Interviewer***	1:47
So you're not doing any specific training for the cross season?

***P19***	1:51
No, other than just general maintenance doing a session here and there on the bike, getting out of weekends, just as a sort of ticking along at the moment, yeah.

***Interviewer***	2:02
Great. So, moving on to the menstrual cycle, so thinking back to when you first started getting your period, what was it like for you? How old were you at the time?

***P19***	2:13
Yeah, I don't know if this is something that girls remember, but I have no idea how old I was at the time, and I was in middle school somewhere. I was alright to start with and then I remember in high school, it started to get quite bad. I would generally pass out on the first day. Get sent home. Yeah. Yes, it was a bit crappy for a little while.

***Interviewer***	2:43
Yeah, yeah, that's pretty crap. In terms of you passing out, was that related to pain or really heavy bleeding or did the doctors not know? Was it just kind of an unexplained, I suppose side effect to starting your period?

***P19***	2:58
Probably a general pain. I wouldn't say pain because it wasn't like level 10 pain. But just like a dull sort of, was enough to just send me over the edge, I think. It wasn't heavy bleeding or anything like that.

***Interviewer***	3:16
Did you suffer like any kind of light-headedness or anything that went with it that may be contributed to passing out?

***P19***	3:22
Yeah, I would just generally feel that I was going to pass out for a while until the point I'd be on the deck, or I’d go and sit down on the floor. It's not just there, if I injure myself it happens. I kind of know when it's coming these days, and I just sit on the ground or lie on the ground if necessary, you know, I know the drill.

***Interviewer***	3:46
And when you said you had a dull ache was that like kind of stomach, lower back?

***P19***	3:53
Stomach. Lower back. Yeah, just generally crap. Yeah. And I wasn't competing in cycling at the time I was doing judo. There was an event that just timing was bad, and it just wrecked it really.

***Interviewer***	4:09
And did you use any painkillers or anything for that?

***P19***	4:13
Yeah. So, ibuprofen seemed to just settle it, yeah.

***Interviewer***	4:19
Yeah. And was that why you went to? Did you go at the GP? Is that why you moved on to taking the pill?

***P19***	4:24
Yeah, just, you know, it didn't feel normal, so went and had a chat. That was his suggestion, and yeah it did help. It helped a lot actually, yeah.

***Interviewer***	4:40
And when you started taking the pill, would you run the packs back-to-back, or would you have that week off and get a withdrawal bleed/period in between?

***P19***	4:50
Early on, I know they recommend that you don't run them back-to-back. I think the doc basically said, you know, it's fine to do that. You know, because for sports and just managing life really. So yeah, I do that a lot of the time. Where it's convenient I'd take a break.

***Interviewer***	5:18
Yeah. And going on the pill, did you have any side effects from that? Any mood related symptoms or headaches or anything related to going on the pill? Or was it kind of pretty symptom free?

***P19***	5:28
I didn’t notice at the time. Don't think so. They did try and switch brands at one point and I just had quite bad depression to be honest. So, I stopped. Yeah, yeah. But yeah, nothing at the beginning, but I probably because I was so young, wouldn't have associated the two, you know?

***Interviewer***	5:51
In terms of you said they tried to change the pill you took, was there any reason for that, or was it?

***P19***	6:00
Just guidelines at the time and one doctor just thought I should. Was quite sort of forceful on it. Really wanted me to change to a different one because of the just lower risk, I guess. Yeah, and it just didn't suit me at all.

***Interviewer***	6:19
And then you just move back onto I think was dianette you took, is that right?

***P19***	6:22
It seems to suit me well and helps with sort of acne issues and things as well which do come back.

***Interviewer***	6:33
Yeah. And I think you mentioned you didn't before you went on the pill. You didn't necessarily consider yourself having heavy periods. It was more just the kind of passing out elements and not feeling very.

***P19***	6:44
Yeah. And just feeling absolutely horrendous.

***Interviewer***	6:49
Yeah. And obviously you said I think you've been on it and since you've been on it, is that allowed you supposed to train and compete without really having to consider your period or your menstrual cycle?

***P19***	7:01
There obviously has been sort of considerations in the past about can you perform better just without taking it. But then you've got the sort of the side effects of, you know, just things falling on the wrong day. So, I chose to just take the sort of level approach, so it was had a consistent sort of training schedule and that worked for me. Whether it's the right thing, there's probably research coming out that might suggest otherwise in future, but it wasn't available at the time.

***Interviewer***	7:38
Yeah. And when you were obviously you competed at judo at a high level. Did you get any advice from the doctors around that then or was it all just through the GP?

***P19***	7:50
Just the GP. I didn't go and ask for it actually. They're probably towards the end, sort of round 2000 and onwards that probably was additional support if I'd sort of approached them to discuss it, but
I didn't realise there was a need to, to be honest, yeah.

***Interviewer***	8:07
Yeah. And in terms of the choice, obviously things really changed since you first went on the pill, did you get I suppose an informed choice about what type of hormonal contraceptive to use. Or was it just suggested this pill and that worked for you, and you stayed on it?

***P19***	8:26
Just because I had acne issues as well and that was the best one at the time. And obviously they have tried to switch things around but weren't as effective and had other associated issues. So ended up going back to that, yeah.

***Interviewer***	8:45
So in terms of cycling, would you just if you had a competition, would you just run the packs back-to-back or would you to try and avoid that kind of withdrawal bleed if you had a race, or do you kind of have that regular week off a pack every month?

***P19***	9:05
Yeah, I would probably just run it through.

***Interviewer***	9:07
So you don't need to worry about any kind of whether it's going to affect training, long rides or racing?

***P19***	9:14
No, I've just never really analysed the impact of that to be honest.

***Interviewer***	9:21
And I think you've kind of covered this, but we haven't really had any specific advice around menstrual cycle in sport. It's all just been, I suppose, through the GP?

***P19***	9:34
Yeah, other than just. I think I probably did this few times if it fitted. I guess if you're not taking the pill, it's kind of be more effective, but I would just sort of time taking things off, taking time off and sort of gaps around competition. So that I’d start taking it again just before competition, that sort of thing. So, I would just see if there's a benefit from that sort of point of view.

***Interviewer***	10:13
I know you don't have a coach now, but if you've had a coach in the past, have you ever discussed your menstrual cycle/periods with them or was it not a topic that's ever come up?

***P19***	10:24
Not really, not in any great depth, just that a bit I guess really. Yeah. I guess just the research wasn't there, other than if you, if you're not taking it, there was a general awareness that you probably would be at your strongest immediately after.

***Interviewer***	10:50
In terms of, I suppose, comfort in having conversations around periods/menstrual cycle, would you ever talk to teammates about it? Any other support staff?

***P19***	11:01
Yeah, I guess I would. Probably not my first topic of conversation, but.

***Interviewer***	11:11
Like most people.

***P19***	11:11
Yeah. Yeah, it's not something that comes up, but it's sort of general, although recently it has, I think there is a bit more awareness and people started to discuss a little bit more what works for them, what doesn't. But that's just in the last few years I've noticed that.

***Interviewer***	11:30
Yeah. So that's kind of my next question. Are you aware there's been great conversation by some elite athletes, particularly likes of Eilish McColgan, Dina Asher-Smith who talk about periods in effect on performance in the media? Has that had any influence on you maybe reflecting on the effect on your performance or training?

***P19***	11:50
I haven't. It's more of a news article. I haven't taken it to heart and changed anything I'm doing as yet.

***Interviewer***	11:57
OK. That's all my questions on periods in the menstrual cycle. Is there anything else you would like to add? Think I've missed, haven't asked you about?

***P19***	12:08
I don't think so.

***Interviewer***	12:12
Moving on to bicycle equipment, I think looking from your questionnaire, you ride a mix of, I suppose, unisex or male bikes and some women's bikes. Have you made any changes to those to suit you? So, I'm thinking kind of crank length, stem handlebars, brake levers?

***P19***	12:36
I've got quite big hands. And my leg length kind of generally suits the men's models. So, I'm kind of lucky in that way for you know, choice point of view. But I have got shorter cranks just to improve the bike fit. And obviously change the saddle. That's it really. My hand has, you know, I've got big hands, so the standard levers and things work for me OK.

***Interviewer***	13:07
Yeah. And when you said you've got shorter cranks, is that a 165 crank or 170?

***P19***	13:11
Yeah, I would have gone 160, but they're just less available yet. So, I went 165.

***Interviewer***	13:18
And you said that was to improve the bike fit. Is that related to any injuries or was it just to be more comfortable when pedalling?

***P19***	13:27
More comfortable but driven from a sort of niggling back injury I've had on one side. That was making me sort of wonky like, well, actually wore a hole in a saddle once on one side. So, had a fit from different people and they both agreed that the shorter crank lengths would work better for me anyway. So that that's why I chose to do that. I think it has helped a little.

***Interviewer***	13:57
Yes, that is my next question. Have you suffered from any cycling overuse injuries? Obviously, you mentioned lower back pain. Have you suffered anything else? So, knee pain, hand or finger numbness, foot pain, saddle sores?

***P19***	14:11
Yeah. So generally, all of the above. Neck and hand pain just generally I've managed to sort out myself with little adjustments of levers and sort of just angles and things. I'm OK with that but getting the right saddle and saddle height and tilt my body just seems to be very, very particular on what works. So, I really have struggled and spent a lot of money on that and had some awful sores over the years. Just an ongoing back injury that I've kind of had for years and it's been sitting niggling I thought was a from the bike but actually I think it's more it's a niggling injury that I've transferred to the bike which hasn't helped and it's off the bike work that I probably need to be doing rather than just relying on a fit to fix it.

***Interviewer***	15:16
Yes, that was one of my questions. Have you modified anything like bike position wise to try and address that? Or is it kind of more related to physiotherapy, strengthening?

***P19***	15:27
For a long time I thought it was the bike that was doing it. Tried all sorts, shifting the stuff around, loads of money on bike fits different saddles. Never really solved the saddle sore issues to be honest, but have a little bit more comfort fit with the back thing that could kind of manage, but it's off the bike work, strength and stuff that I know is the answer to that one.

***Interviewer***	15:54
I think in terms of your pre interview questionnaire, you ride four different saddles. How did you arrive on those? Yeah. Because as I say, you've got mix and match. Is it based on some of them the difference is it based on the type of position between I suppose TT, cyclocross, road that you have different ones?

***P19***	16:14
Yeah, that well, the cyclocross I pay less attention to because I'm on it less, spend less time on it and events are shorter. So, I've used the one that kind of used to work on another bike and I can get away with that there. The TT bike, I tried quite a few different saddles, and I've got one that works, and actually is probably the most comfortable out of all of them. And I don't touch it. I don't lend it to anybody. I don't move it off. It stays where it is. Road bike have struggled a lot and just gone through lots of saddles. Had quite an expensive bike fit a couple years ago and they recommended a saddle. Got it into the most optimum position, but it was never, still wasn't perfect, yeah.

***Interviewer***	17:01
So in terms of you mentioned you had a bike fit there, but in terms of I suppose getting on those saddles you ride now, is that mainly been trial and error?

***P19***	17:11
Yeah, if you accidentally clean your bike, take it apart a bit you've nacked the exact position it was in and finding again I find really difficult.

***Interviewer***	17:24
Do you put a tilt on your saddle or you flat?

***P19***	17:28
So it is a slight tilt down. I probably ride quite aggressively, as I've been told.

***Interviewer***	17:35
And was that a recommendation from bike fit or just something you discovered worked better for you?

***P19***	17:41
Probably bike fitter helped with that initially, but equally you know if I've tilted it back up to have sort of moved it around to the place that is best I can find at the moment.

***Interviewer***	17:57
And you've mentioned you have suffered from saddle sores in the past, some quite bad. Where would you get a saddle sore? Would it be sit bones, upper thigh, outer labia, inner labia, that kind of area? Or is it all of them?

***P19***	18:13
I've had all of those. At the minute it's probably sit bone area.

***Interviewer***	18:23
And what type would it be? Kind of cysts, pressure sores like pressure sores, pain, spots, whatever, chafing.

***P19***	18:33
Yeah, all of that. So I found it, like using chammie cream I was using more and more which I think had a reverse effect which is block the pores, caused more chaos. I stopped using it all. A recent sort of trip, saddling 6 days in a row. I would use some light cream on the shorts with some sudocrem on the sores day-to-day.

***Interviewer***	19:00
Help repair them.

***P19***	19:02
I'm starting to think why do I do this at all? But yeah, they yeah, it comes up in like little boils.

***Interviewer***	19:12
Have you ever had seek medical advice for those, or do they with rest and sudocrem heal themselves?

***P19***	19:13
Yeah. Sometimes I pop it. Sometimes I know that was a bad thing to do. Sometimes it just you kind of know it will heal quicker. It's just, you know, trying to work out what's coming up, what you're planning on doing over the next couple of weeks to see what's going to be the best way to let it heal.

***Interviewer***	19:41
But in terms of what you've done, you've never kind of suppose sought medical advice about the best way to manage them.

***P19***	19:49
No.

***Interviewer***	19:51

And you've never had to take antibiotics, right?

***P19***	19:52
No.

***Interviewer***	19:55
And is it, you obviously mentioned on a recent trip you had a bit of a problem. Are there any factors that make it worse, such as time trialling, time length of time on the bike, number of rides back-to-back, days riding?

***P19***	20:08
I think shocking roads in the UK actually, just the bumpy roads. Just are significantly different. Did notice that when I came back. I'd gone out with just a bit sore, and it probably stayed about the same over the time, which I was lucky with. It didn't get worse which I would have done if I'd done that sort of riding over here.

***Interviewer***	20:35
Yeah. And what about time trialling? Is that an issue in terms of developing a saddle sore or was that not a problem?

***P19***	20:44
Oh, that was horrendous as well. I went through a number of saddles, but the saddle I had. I've done 100 miles on it and been more bruised at the end rather than having sores.

***Interviewer***	20:58
So it's more just kind of bruising just from the length of time sat there.

***P19***	20:58
Yeah.

***Interviewer***	21:04
And is that on your current saddle? I think you ride the cobb. Is that right?

***P19***	21:07
Yeah, it is. It wasn't the one I wanted. I picked up accidentally. It's ugly, a sin, but. Yeah, I think cobb Max. I think I had a look on it and put it down on the form.

***Interviewer***	21:22
Obviously mentioned you use sudocrem to help repair them and maybe pop them. Have you ever had to take painkillers or have an extended time off the bike for a saddle sore?

***P19***	21:32
Yeah, I’ve taken, I just chose not to ride rather than an extra session because I thought that would be better, more benefit. Yeah, yeah, definitely. I think I've probably tried ibuprofen a couple of times which you know, unless it's a bruise and swelling, and sometimes it is. It's not going to do a lot, yeah. Cold water, I found actually quite good.

***Interviewer***	21:57
Yeah. If you can stand it. Yeah. And it sounds like the saddle sores have affected your like, I suppose enjoyment on the bike and ability to train and compete.

***P19***	22:08
Yeah, yeah, yeah, a little bit. I did just try to ignore it completely for a while and made it quite bad. Depends what's coming up.

***Interviewer***	22:22
And you mentioned like doing some of the really long distance TT’s. Do you just accept that some pain is part and parcel of time trialling that distance?

***P19***	22:31
Yeah. I've got an old neck injury from judo in the past as well that I know will also crop up on a 50-100 miler and it's just accepting it's a long race, so will be slightly uncomfortable. I would need to do a lot more training to make my body get used to that sort of distance comfortably, you know. I haven't got time.

***Interviewer***	22:57
Yeah, I suppose with the sadlles you've got now when you kinda just accept and that's the best that's gonna get. Or would you have another go to try and find a new saddle?

***P19***	23:07
If there was another way to find your perfect saddle without spending a fortune, yeah, I would generally be interested. I don't think it's perfect, but particularly on that bike it's. Yeah, I know. I've done all sorts of things in the past and I haven't touched it, so it's staying.

***Interviewer***	23:26
We have obviously talked about saddles and you kind of got there by trial and error and you talked about when you use too much chammie cream wasn't a bonus. Have you done anything else?
Obviously, put a slight tilt on your saddle, but if you get saddle sores and you've got a lower back pain, is any of it related to get one sided saddle sore in terms of if it's a one sided lower back pain, does that cause a problem?

***P19***	23:51
Yeah. So, for a while, I was convinced because a chiropodist told me that I needed an insole when I was running that that was possibly it, you know. So, I started putting shims in. Had a bike fitter to help me with that and I think it actually just caused a lot more problems and enhanced the imbalance that was a lot of one sided. Now my issues do tend to be symmetrical, which weirdly I'm happier about.

***Interviewer***	24:20
Have you ever find influence of shorts, the choice of shorts, do you ever only ride a certain type of short or with a certain type of chammie in it? Or is that do you not find that really has much influence?

***P19***	24:39
I kind of said my body feels the slightest change. Weirdly I used to race an old skin suit I had that was almost threadbare because it had a really fine pad in, really thin pad, which was sort of didn't seem to make sense, but could get away with it. So, I'd quite often turn up to races in old kit because I knew that was better for me. But I have been using ASOS shorts for quite a while, but I'm starting to think actually that pad maybe it isn't actually the most optimum for me, so I'm looking around again.

***Interviewer***	25:19
And whenever you've been, have you. When you've been a cyclist, have you ever had a coach or have you always self-coached yourself?

***P19***	25:27
I had a chat with the coach, but I never actually sort of signed up with them officially. They just give me some guidance on the tools to use and how to measure performance and training a little bit of it. A little bit of it was just so close to my old judo coach, who I didn't pay. It feels disrespectful to pay for a cycle coach when I'm just sort of, not competing at such a level so that's a part of the reason why I've never actually signed up to be coached fully.

***Interviewer***	26:02
Yes, you've never discussed your saddle sores with anybody. If you mentioned them when you've been for bike fit.

***P19***	26:08
Yeah, yeah, definitely mentioned them at a bike fits. Even went to one of those expensive ones where they put you want to sort of electronic pad, so they can see where the pressure is. And even like after trying 30 saddles, the one I had looked the best, it's the best he could find, but you could still see in the picture it wasn't perfect, so I thought I'll try it and maybe see, you know that was, you know, just fine tuning, but yeah it was never perfect.

***Interviewer***	26:44
That's all my questions on kind of overuse and saddle sores. Is there anything you think I've missed or would like to add around this?

***P19***	26:52
No, I don't. Hopefully there was something useful in there.

***Interviewer***	26:57
Yeah.

***Interviewer***	27:00
My final question, are there any areas you think would like us to do research in not just me specifically, but scientific community in regard to female cycling? So menstrual cycle, female specific training, bike setup, saddle choice, injuries.

***P19***	27:17
Yeah, I think it just feels like there needs to be a bit more work on saddles for women. Yeah, the men do not have the same conversations about problems, it's very once in a while, whereas just groups of women, they all suffer from, you know, a range of issues. So, there's clearly more work needs to be done there. That's the main one for me, I guess. Just more research on effective training for women and their menstrual cycles because there just isn't enough information on how it affects them.

***Interviewer***	28:00
OK. That's great. Is there anything else you'd like to add before I turn off the recording?

***P19***	28:04
No, don't think so. All good.

**P20 – interview transcript**

***Interviewer***	0:05
So just to start with a bit of background about you and cycling. So how long have you been cycling and what events do you compete in?

***P20***	0:17
I started in 2015, and I got a bike on the cycle to work scheme so that was really how I started, and I was riding to work every day and I just got addicted. Didn't have a clue what I was doing really, but I did used to run a bit before then and I just kept getting injured and I got into spinning. And then I finally bit the bullet and got a bike on the cycle to work scheme and yeah, that was it really. Then it escalated quite quickly because basically my husband and I, we signed up to this event which was an ultra-duathlon across Scotland and looking back it was in complete naivety really about what it entailed….

***Interviewer***	1:37
Yeah, Scotland.

***P20***	1:38
With a westerly headwind the whole way. But yeah, I ended up doing quite well because not many people finished, but I did manage to finish, and I ended up coming 2nd and I think that inspired me quite a lot. And my mum encouraged me to enter an Ironman off the back of that, which I then learned to swim for really because I haven't done any swimming in adulthood. And I got because I couldn't swim, I had a coach to help me swim and then he was a triathlon coach, and then he ended up giving me triathlon training for cycling, and then he encouraged me, like he saw my potential and encouraged me to invest in time trial bike, which I was lucky to get the support of my dad to help me do that. And when I started riding the time trial bike, it just yeah, I just took to it really quickly and then I did my first Ironman in that same year I competed in my first time trial, so that would have been 2018, so just three years after I got my bike on the cycle to work scheme. And then yeah, I was completely hooked. So after then I was doing an Ironman like every year and then doing time trial races around that. Then during COVID because lots of things were cancelled apart from the time trialling, I decided to enter a 12 hour time trial, which I'd only really learnt about the concept of that a year prior. Yeah, when I did that event, it sounds weird, but I just really enjoyed it and I felt like I found my thing and then the following year I did another 12 hour and that happened to be the national championships….

***Interviewer***	3:57
Yes.

***P20***	3:58
So and then that obviously I got a bit of attention off the back of that, and I was approached in 22 to ride for …. a women's team because they hadn't previously had a women's team and yeah, so that's what I did. This past season, this past year has been the first time I've just focused on cycling, so last year I did, last year was my first year for the team and I was focusing on cycling, but earlier on in the year I was training for an Ironman, but then I got injured and then I was like, oh, I'm just going to stop running because I just keep getting injured. So, I focused on cycling, but then I had a last-minute surprise entry to Kona at the Ironman World Championship. So, I did do that last year. So last year was quite mixed, but this year I've just been doing the cycling bit. I've learnt that actually my body feels a lot better doing all three things. I thought I thought it would be, I'd get loads stronger and I wouldn't have any injury problems but then you get a whole new set of problems, because I'm, like, crunched over like that all the time and my hips are closed all the time. So yeah, I've started doing triathlon training again, which is good.

***Interviewer***	5:22
So, how's the season gone in terms of competitions?

***P20***	5:27
I haven't had the best year actually..…..

***Interviewer***	7:57
Is that through the GP that you're getting help with that?

***P20***	8:01
I am at the moment, but I have been given recommendations for going privately as well. But I'm going to see how my experience is with the GP in the first instance.

***Interviewer***	8:18
So, moving on to your menstrual cycle. So, I also thinking about to when you first started getting periods, what was that like for you? How old were you at the time?

***P20***	8:31
I was 13 and I don't really remember it being anything that was too horrific. Like, I think they were quite regular, they weren't particularly long, like, they were uncomfortable, but not massively so. I was quite sporty when I was younger, but not in running and cycling, but I used to do, I was basically horse mad, horses when my life and I did a bit of athletics and some team sports at school. I was quite active. I never remember it stopping me from doing anything. But then I went on the pill about I think I was 17 or 18. And I remember at the time I was, I don't really remember why I did that like looking back. Maybe I had like skin problems, or. I remember the doctor encouraging me to go on it. And now when I look back, I just wish I'd questioned that more or looked into it more. Because then you know I was on the pill for quite a long time and then when I came off it, I didn't have any periods for a really long time which I had explored medically. And couldn't really get to the bottom of anything and at the time I was told if I wanted to have children that I would have to go down the IVF route which I didn't do. And then they just came back, like, eight or nine years later which is quite interesting. But yeah, I don't know if that was related to periods or whether it was related to other things, sorry the pill or whether it was related to other things, I don't know because I remember I always pronounce it wrong. Is it amenorrhea when you don't have any periods for a long time?

***Interviewer***	10:47
Yeah.

***P20***	10:49
I remember the doctor. I was having tests for osteoporosis and polycystic ovaries and I got recommended to go and have acupuncture for stress and so I don't, I don't. I did have quite a stressful job at the time, so maybe that was related to it, and I was really, there was a period I was really into fitness as well, but probably, you know, in a weight loss way, not that I was particularly thin but I don't think that was great either, like I used to do fasting, intermittent fasting and yeah, I used to train a lot, like that was before I discovered cycling and triathlon, really. So yeah, maybe that had something to do with it as well.

***Interviewer***	11:40
Yeah and going back to when you first start getting periods. Did you get any advice around periods,
menstrual cycle or was it just kind of standard school chat when they started?

***P20***	11:50
Standard School chat was quite funny because I went to a girl’s school, so a lot of us would have our periods at exactly the same time. I don't really remember it being discussed other than you know everyone being quite highly strung for certain periods of the month.

***Interviewer***	12:12
Yeah. And obviously you said you weren't, you can't really quite remember why the GP recommended you go on the pill at the time, did you have much pain or mood symptoms with your periods in your teenage years, or would you just put that down to being a teenager?

***P20***	12:27
I don't really remember it now if I'm honest, but I really don't remember, so it couldn't have been that bad. I don't remember it being like a really big traumatic thing and that I needed help with.

***Interviewer***	12:38
And then when you were on, I think you on the combined pill, did you have like a kind of week's break each month and have a withdrawal bleed or a period when you were on that, or did you run the packs back-to-back?

***P20***	12:55
I can't remember. It's such a long time ago.

***Interviewer***	12:59
Don't worry if you can't.

***P20***	12:59
It must have been back-to-back. I don't remember there being a gap.

***Interviewer***	13:04
So you didn't have, so you didn't have any like kind of periods/bleeds during your 20s while you're on the pill?

***P20***	13:12
I did, so I must have had a break, because you come off it and then you, yeah, that must've been how it worked. Yeah, and it was just very short.

***Interviewer***	13:21
And that didn't cause you any problems in terms of sport, or participation, anything like that?

***P20***	13:30
No.

***Interviewer***	13:32
And then you obviously said it took you quite a long time for your periods to return when you came off the pill and when they came back, how, how have they been, obviously changes this year but before that?

***P20***	13:44
So at first it was really irregular and very heavy and painful, like, it would really like wipe me out. And then over time they got more and more regular to the point, now they're pretty much like clockwork. There's been an odd occasion. Often that has been when I'm training for the 12 hour TT or something like that there's been some delays, yeah, a very slight delay, but typically it's been super regular, both in terms of when it comes, how long it lasts and how heavy it is.

***Interviewer***	14:31
In terms of physical symptoms, you get period pains with them. Do you have to take painkillers for that?

***P20***	14:39
I do, yeah.

***Interviewer***	14:41
How long does that do you? How many days do you have to take painkillers for?

***P20***	14:45
It's the first day, really, the first day to the second day. I find that the first two days it's awful, and then it pretty much goes, and it's doesn’t last very long, it's like three to four days long.

***Interviewer***	14:58
And does that affect it? If you're racing or training around those first couple of days to make any changes for that?

***P20***	15:14
Not really, other than psychologically giving myself a break, so I'll be like, OK, leading up to my period that week up to it, I know that I'm not going to feel great and I'm going to feel like I haven't quite, you know, got the energy to do stuff or the strength to do stuff. I'll crack on and do my training session but then if I'm really struggling, I just, you know, well push the pedals a bit lighter or you know, and I won't beat myself up about that because I know what's going on. And occasionally I have my period has come on the day that I've been racing and it's I've just felt, yeah, not good. The next day I feel amazing, in terms of my strength, but the actual first day of the period, my strength just it definitely isn't there.

***Interviewer***	16:06
So does it just feel harder? Would you describe it just feels harder and you feel more tired, is that?

***P20***	16:19
I think I'm a bit, my body temperature's higher slightly. I mean, I don't know that for sure because I don't wear like an oura ring. Yeah, I don't wear anything like that, but I feel like a bit hot in myself and it's almost like there's a limit. It's almost like something stopping me, like I literally can't push past a certain point. And it is not even the feeling of like when you're training normally and so, you have really hard effort, you do a really hard effort to when you just essentially just fail at outputting that power. But on the first day of my period, it feels different to that, it almost feels like I don't know, it's like this invisible thing that's just stopping me from even getting my heart rate that high, or even getting to that level, if you know what I mean. And it was a bit like that in the 12 hour this year actually, it was almost like my body was just going, no, you're not doing anything more than that, you know.

***Interviewer***	17:27
How does sitting in time trial position affect if you get, I think obviously get cramps and I think you said lower back pain, is that a problem sitting in aggressive TT on the first day?

***P20***	17:43
It does feel a little bit uncomfortable, but it's not something I've had to do a lot because if I'm training on that day, I just sit up, you know, I won't worry about it too much. It's been a very rare occasion that I've had to actually race in that position.

***Interviewer***	18:02
Do you get any kind of mood related symptoms with your periods? Maybe that premenstrual phase?

***P20***	18:11
Yeah, the week before, I'd say that my patience is definitely thin. And yeah, I get agitated at things that normally I just wouldn't bother me at all.

***Interviewer***	18:24
In terms of training around your periods and you said they're obviously regular now, would you say you have heavy bleeding or is it just very normal?

***P20***	18:35
It’s heavy for two days and then it's pretty, it's really light for two days and then that's it, it's all over.

***Interviewer***	18:42
So does that affect you going for a long ride in those first couple of days if it's really heavy, if you need toilet stops or whatever, or are you OK?

***P20***	18:49
I'm OK. Yeah. I mean, I haven't. I can't remember having to do any more than like 4 hours in that situation.

***Interviewer***	19:01
So you wouldn't modify your training around that kind of first few days of the period if you had a scheduled road ride or anything?

***P20***	19:09
No.

***Interviewer***	19:11
And do you ever mention your periods to your coach to discuss that, like mention, if your power is down or you feel a bit rubbish?

***P20***	19:18
Yeah, I do. I always put it, I use Training Peaks, and I always put it in Training Peaks like whether it's leading up to it or the actual day and there's, you know, a note in there so that he can see like when it's happening as well.

***Interviewer***	19:33
Does he make any changes around kind of training program around your periods or do you just kind of keep it as normal?

***P20***	19:42
No, he doesn't, keeps it as normal, but then if he knows that I'm feeling a bit rough, he'll just say, ah, go easy, you know, do whatever you feel like doing. He always puts it on me to say, do what you feel comfortable doing. I mean the reality is because of like how I am mentally, I'm like, I'm going to do it anyway, but I just I give myself a break. I am not like, you know, oh, I can't do it, it's terrible. Or I just like, you know, take it a little bit easier or knock it back a little bit.

***Interviewer***	20:14
Do you have any times of the month where you feel better, like more motivated, stronger?

***P20***	20:28
Yeah. I would say typically that first week after my period. Something that I've noticed this year is the mid cycle, which I guess is when I'd be ovulating I also feel a bit rubbish. So, it feels like actually I only feel really good like the week after.

***Interviewer***	20:57
And do you track your cycle on anything or?

***P20***	21:05
No, just calendar really. I just keep track of it mentally and because it is quite regular, it's quite easy to do that.

***Interviewer***	21:15
Yeah, and this is quite a specific question, but do you ever feel experienced any changes in core strength, balance, flexibility throughout the menstrual cycle?

***P20***	21:28
Yes, I am quite a clumsy person anyway, but I would say that around the time of my period I think I'm worse and I think my balance is slightly off then as well. I haven't noticed my core strength change because I don't know, I haven't really thought about it before. Maybe that does affect me in the pool, but I'm not the best swimmer anyway, so it always feels like a struggle so.

***Interviewer***	22:01
So in terms of like balance, I think you said you do like at least one S&C session a week. Is it more like in that sort of session, you'd notice?

***P20***	22:09
Yes. Yeah.

***Interviewer***	22:11
Would you make any changes around that, or would you just do as programmed?

***P20***	22:16
I would just do as programmed.

***Interviewer***	22:20
In terms of racing, I think you said you've only had a period fall on race day a few times. Would you do anything around that? I know we can't change race days. Would you not race or miss a race or schedule it around that?

***P20***	22:36
I wouldn't really. Like it has happened on some really important races before, so I've had one Ironman and thankfully it the period it was day two of my period rather than day one, and when I won my first National 12 title, that was on day two of my period as well. And I mean, it's not going to stop me from doing it, but what I have done is my nutrition, I've definitely taken on board more.

***Interviewer***	23:15
Yeah. Is there anything else you do to help or is it just kind of, yeah.

***P20***	23:21
Making sure that I'm really fuelled and when I did the 12 hour, I took painkillers but I didn't do that in the Ironman.

***Interviewer***	23:30
And have you ever had any advice around like the menstrual cycle and sport at all?

***P20***	23:40
Only book that I've read myself. I haven't had any proper advice on it.

***Interviewer***	23:45
So is this book by other athletes or coaches that you’ve read?

***P20***	23:50
So I have forgotten her name. Look up on my thing. Stacey Sims. I've read Stacey Sims - Raw and Next Level, which I found quite useful.

***Interviewer***	24:05
Are you aware that more kind of elite athletes are discussing the effect of periods in the media? So, the likes Dina Asher-Smith or Eilish McColgan, have you seen any of those stories?

***P20***	24:21
I actually haven't seen their personal stories, but I have seen other athletes. So I saw the whole thing around Emma Pallant because I think her period came during a race and it was actually visible that her period had come, and I thought it was really good how she spoke about that afterwards. I have noticed the lady who won the Ironman World Championships in Nice this year, Laura Phillips. I heard her interview like pretty much immediately after the race and she talked about how the timing of the race, she actually mentioned I knew the timing was good, that I would be feeling strong in my menstrual cycle, and I thought, you know, well for her to say that like after she just finished, I thought that was really good. It made me think, oh, you know, they are thinking about these things, so I am aware that it's becoming more talked about than it used to.

***Interviewer***	25:21
Has that any influence on you in terms of thinking about your menstrual cycle or whatever?

***P20***	25:27
I think the reality is I don't think it would change my plans to race or not to race. It would just make me, you know, because when you're in a race you're thinking about, oh, how do I feel? Can I push this a bit harder and hold on to it? can I, you know, you're thinking about so many things, and it would just be those thoughts would be thrown into the mix of I'm not quite feeling like I've got the edge today but that's probably why, but just, you know, try and manage your expectations around that or manage yourself around that.

***Interviewer***	26:06
In terms of having conversations about the menstrual cycle, obviously you said you mentioned it in Training Peaks to your coach. Do you talk to your teammates at Team Bottrill about it or?

***P20***	26:16
I do. I've spoken to them about it a lot. There are five of us in the women's team and two of the girls wear an oura ring, which they talk about a lot. My view like you've got this information but what are you doing with it? You know, and they're very aware of their body temperature and where they are in the month and blah blah blah. And we do talk about like how we felt with regards to that and in certain in like the big championship races, those are the only races that we all go and do together and there will be talk about who's where in their cycle. So yeah, which is it's nice really, because it's like, oh, yeah, everyone's in the same boat, aren't they?

***Interviewer***	27:08
In terms of obviously you put notes in Training Peaks, but do you talk to your coach about your periods or is it just more like notes, you're not feeling great today?

***P20***	27:19
I talk to it in relation if my ability to hit certain numbers or not, and how strong I'm feeling. We've also talked about perimenopause. It was quite funny actually, I because I had been thinking it because I'd had discussions with friends at the same age as me. I've been mulling it over for a while, but I haven't really mentioned it to him, and he brought up with me at one point and he was so worried about doing it, I could tell, but it was good, like we're on the same page on it so.

***Interviewer***	28:12
That's all my questions around kind of periods and menstrual cycle. Is there anything else you'd like to add? Think I've missed asking you about?

***P20***	28:21
No, not really, no.

***Interviewer***	28:24
So moving on to kind of bike equipment and set up, you obviously got a road and TT bike I think. I can't remember, whether you said the women's specific or not. Have you made any adaptations to those to suit your set up? So, thinking kind of cranks, handlebars, saddle, etc.

***P20***	28:43
So the road bike is sold as a woman's bike, and I think the handlebars are just slightly narrower than the men's bike I think that's the only difference. And yeah, other than that, I probably need a different saddle on it, in fact, I'm fairly certain I need a different saddle. I haven't ever really invested in a bike fit on that bike. I think I had one when I originally got it and then and then that was it. The time trial bike is a bit different because I've had a number of bike fits, and I know it's been adjusted to me and whether that is a woman thing or it's just a me thing. It's setup for me.

***Interviewer***	29:32
And with the time trial bike, I presume when you've been setting up some of that is about being as aerodynamic as possible, more than comfort?

***P20***	29:40
It is, but it's being comfortable because if you're not comfortable, you're not going aerodynamic you’re not going to be able to hold it. So, comfort is a big thing, particularly with the type of racing that I do and Matt, who runs the team that is, you know, he's thinking about that when he looks at your setup.

***Interviewer***	30:01
And have you had any cycling overuse injuries? So, thinking knee pain, lower back pain, foot pain, hand or finger numbness, saddle sores.

***P20***	30:10
When I was cycling to work a long time ago, I had carpal tunnel but I haven't had that for a really long time, not since then, really. Well, when you're doing, like, ridiculous training for a 12 hour time trial, you are going to get tight shoulders and you're going to get tight hips. But during the training, I think there's only been a few times when I've had lower back pain. Yeah, maybe my shoulders have been particularly tight, I find that actually leading before my period that might be a bit more likely than it is afterwards, so yeah, but nothing too significant. So, I've always been. I've always invested in my setup to, you know, eliminate those problems.

***Interviewer***	31:17
Yes. So, I suppose, yeah. In terms of things that make it worse, I presume, just mainly the really long stuff, if you're doing a lot of training for the 12 hour, it's more likely to get feel a bit tight. Is that right?

***P20***	31:28
Yeah, because you're just in the same position for hours and also, you're on the road so, you're like tense from the danger of the situation as well.

***Interviewer***	31:35

In terms of your saddle, how did you choose your current saddle? Was that trial and error or did you just get lucky with what you had on?

***P20***	31:48
So it was recommended to me based on the bike fit and Matt does saddle pressure mapping as well. So, I feel that in the past for a number of years I had a different saddle on the time trial bike and the setup I've got now is so much better.

***Interviewer***	32:12
And do you ride a totally flat saddle, or do you have a slight tilt nose down?

***P20***	32:19
I don’t actually, know. I think it's totally flat.

***Interviewer***	32:24
And I think you said previously that you have suffered from saddle sores. What makes this worse? Is it the long stuff, time trial position?

***P20***	32:35
Yeah. So, when I did my 1st 12 hour. I, yeah, I did get some problems down there. I had an infection, and I think essentially it was an ingrown hair that then got like massive and infected, and I just felt really ill, and I was put on antibiotics.

***Interviewer***	32:57
Was this post race?

***P20***	32:59
Pre-race, because I remember I didn't want to go on the antibiotics because I was worried about how they would make me feel before the race, but the doctor said if you don't take these, you're not going to be doing anything. So, I got on and did it. But yeah, I learned quite a lot then about how actually it's good not to shave down there and, yeah, just making sure, because I was also thinking to myself, oh, I'm going to try and put up with the discomfort, because then I'll feel loads better during the race when it's really long so I was like not using chammie cream and things like that. Whereas I've just learned that that's really important. So yeah, I haven't had a problem like that since then.

***Interviewer***	33:50
So did you. Was it just a GP you saw when you had that bad saddle sore before the 1st 12?

***P20***	34:02
It was, yeah. I mean when I have done a 12 hour like because I've now done four and after each one you are sore down below like there's no, you know, there's no escaping it, but it's more like the creases of the top of your leg and groin area I find. And yeah, I have had like swelling down there after the main race, but I don't remember it being bad in training for some time now.

***Interviewer***	34:43
So in terms of after the time trial is it just swollen, feel bruised from the pressure of sitting in time trial position?

***P20***	34:49
Yeah.

***Interviewer***	34:51
And after since the first one, if you had any problems with kind of the ingrown hair or anything getting infected, or if you've been OK since then?

***P20***	34:51
Yeah. I think I did. I think I've had it happen twice since, but after the actual main race, but it didn't get to a point where I needed to go to the doctor. It was fine like I just, you know, used sudocrem and yeah, just didn't do much for that week and then I was fine.

***Interviewer***	35:23
Yeah. So, is your problem mainly right at the top of your thigh? Do you get problems with sit bones or are they OK?

***P20***	35:31
It's mainly at the top of my thigh, yeah.

***Interviewer***	35:35
And you said obviously you've learned that you need to use chammie cream. Is there anything else you've done, you've changed your saddle? Does kit, the type of chamois, the shorts, does that play a role in it? Have you changed that?

***P20***	35:47
Well with the skinsuit, you're quite limited with, you know, it is what it is really. What I have started doing for the main race is in addition to the chammie cream, I would also use sudocrem as well. So I've got that like antiseptic thing going on already. But yeah, I think my position now is better than it's ever been before, so that definitely helps.

***Interviewer***	36:16
And is it a problem for all time trials? Is it mainly just the really long stuff that you do?

***P20***	36:21
It's the really long stuff.

***Interviewer***	36:23
So you'd be OK for a 10 or 25 mile?

***P20***	36:27
Oh yeah, definitely. And I do find as well that you know, the more training you do, the more, you know you just get, your skin and whatever, it just gets used to it. Because my teammates, they don't really do a lot of long stuff, but they will do the 100 mile time trial, the National 100 mile and that's like it's going to take them three hours 45 to four hours. And all of them are like complaining about how sore they are as a result of that, whereas that doesn't bother me at all, because normally around that time I'm already like, really well into my 12 hour training.

***Interviewer***	37:11
I'm presume if you done quite a few 12 hours now and you all can end up sore the end of it, you just kind of used to tolerate in the pain and you know, you can just manage it mentally.

***P20***	37:20
Yeah. I mean, yeah, it is what it is, during the race actually I don't actually feel the saddle issues at the time really. There might be a couple of periods where like, it's more from the pressure but the soreness in the saddle area comes after the race. Whereas during the race you've got like the shoulder tension, but then also my feet have historically been a big problem as well and I have had adjustments made to help that, but the thing that always sticks in my mind is the foot pain.

***Interviewer***	38:04
Is that just a 12 hour thing or is that or does that affect you?

***P20***	38:08
Yeah. It's just a 12 hour thing really, because you've got carbon shoes on your feet for that long, and the race is in August typically, so, it's going to be warm and yeah, there's like, there's no relief. You're pushing those pedals the whole time, and if your feet swell with the heat, dehydration, you're going to get problems. This year, it's the best it's ever been but last year I had my shoes broke just before the race. So, I had like these shoes which weren't quite right for me and oh my God, like after I finished, I couldn't stand up for a really long time and my feet were burning like I had that hot foot, burning nerve damage thing going on. It was awful. Just thinking about it now almost makes my eyes water like the pain was just unbearable. But yeah, I didn't have that this year.

***Interviewer***	39:10
And in terms of that, you've mentioned in terms of if you get a saddle sore, you might put a bit of sudocrem on there you haven't had to do antibiotics again. Do you do anything else, or do you just take a bit of time off the bike to let it heal?

***P20***	39:22
I take a bit of time off the bike because the reality is those feelings are happening after I've done a 12 hour race, so I won't go near a bike for a week anyway if I have saddle sores or not.

***Interviewer***	39:33
And do you ever mention saddle sores to your coach to plan around that? Or do you just get on with it?

***P20***	39:43
Yeah, I remember like this year because normally he doesn't put anything in my training plan and he'll say to me whenever you feel ready, let me know and I'll start putting some things back in and you know I'll just send him a message saying just to let you know, there's no way I'm sitting on a bike anytime soon.

***Interviewer***	40:09
Yeah, I think that's all my questions around bike setup and saddle sores. Again, is there anything you think I've missed asking about or you want to say?

***P20***	40:17
No, that's all really.

***Interviewer***	40:20
And then final question, are there any areas you'd like researching regard to female cycling, so thinking about the menstrual cycle, female specific training, bike setup, saddle choice, injuries?

***P20***	40:38
I mean, I am very interested in how, what's going on with the menstrual cycle and how that's affecting how I feel and what I can do but now it's like perimenopause and but I know that it's well from what I gather it's such a complicated thing and it's not one size fits everybody so learning about that is definitely a journey that I'm on.

***Interviewer***	41:11
Is there anything else?

***P20***	41:13
No.

***Interviewer***	41:14
Would you like to add anything before I stop the recording?

***P20***	41:18
No, that's all.
